# Fungal Endophytes: A Potential Source of Antibacterial Compounds

**DOI:** 10.3390/jof8020164

**Published:** 2022-02-08

**Authors:** Sunil K. Deshmukh, Laurent Dufossé, Hemraj Chhipa, Sanjai Saxena, Girish B. Mahajan, Manish Kumar Gupta

**Affiliations:** 1 TERI-Deakin Nano Biotechnology Centre, The Energy and Resources Institute, Darbari Seth Block, IHC Complex, Lodhi Road, New Delhi 110003, Delhi, India; 2 Agpharm Bioinnovations LLP, Incubatee: Science and Technology Entrepreneurs Park (STEP), Thapar Institute of Engineering and Technology, Patiala 147004, Punjab, India; ssaxena@thapar.edu; 3 Chimie et Biotechnologie des Produits Naturels (CHEMBIOPRO Lab) & ESIROI Agroalimentaire, Université de la Réunion, 15 Avenue René Cassin, 97744 Saint-Denis, France; 4 College of Horticulture and Forestry, Agriculture University Kota, Jhalawar 322360, Rajasthan, India; hrchhipa8@gmail.com; 5 Department of Biotechnology, Thapar Institute of Engineering and Technology, Patiala 147004, Punjab, India; 6 HiMedia Laboratories Pvt. Ltd., Mumbai 400086, Maharashtra, India; girishbm2000@gmail.com; 7 SGT College of Pharmacy, SGT University, Gurugram 122505, Haryana, India; mkgupta5@gmail.com

**Keywords:** endophytic fungi, antibacterial compound, natural product, drug resistance, medicinal plant, AMR

## Abstract

Antibiotic resistance is becoming a burning issue due to the frequent use of antibiotics for curing common bacterial infections, indicating that we are running out of effective antibiotics. This has been more obvious during recent corona pandemics. Similarly, enhancement of antimicrobial resistance (AMR) is strengthening the pathogenicity and virulence of infectious microbes. Endophytes have shown expression of various new many bioactive compounds with significant biological activities. Specifically, in endophytic fungi, bioactive metabolites with unique skeletons have been identified which could be helpful in the prevention of increasing antimicrobial resistance. The major classes of metabolites reported include anthraquinone, sesquiterpenoid, chromone, xanthone, phenols, quinones, quinolone, piperazine, coumarins and cyclic peptides. In the present review, we reported 451 bioactive metabolites isolated from various groups of endophytic fungi from January 2015 to April 2021 along with their antibacterial profiling, chemical structures and mode of action. In addition, we also discussed various methods including epigenetic modifications, co-culture, and OSMAC to induce silent gene clusters for the production of noble bioactive compounds in endophytic fungi.

## 1. Introduction

Over the decades since the discovery of the first antibiotics, resistance to those has been a curse that is being dragged along with every discovery of new antibiotics. This has kept all scientists, professionals, and clinical specialists working on antibiotics on their toes. The quest for new antibiotics scaffolds and repurposing of existing molecules has been persistent for the past nine decades. Getting a new and right scaffold is a herculean task, especially with the least ability to induce mutations in the target bacteria. As examined in some of the earlier reviews [1,2] there are several ways of getting new scaffolds and classes of antimicrobial bioactive compounds. In the domain of natural products, one of the most demonstrated ways is studying less explored species and genera of microbes [3,4,5]. Investigating unexplored ecological units on the globe synergizes with the concept of investigating the least or not explored species of microbes.

In the current review, we present the latest ways of exploring the credentials of such microbial sources, especially endophytic fungi, as a main stream of novel antimicrobial scaffolds. Bioactive compounds are mainly responsible for the activity profiles displayed by endophytic fungi. These metabolites belong to a wide range of scaffolds such as alkaloids, benzopyranones, chinones, peptides, phenols, quinones, flavonoids, steroids, terpenoids, tetralones, xanthones, and others. Moreover, they, in the pure form, have demonstrated abundant biological activities, including antibacterial, antifungal, anticancer, antiviral, antioxidant, immunosuppressant, anti-inflammatory, and antiparasitic properties [6,7,8,9,10,11,12,13,14,15]. Even though there are a few specialized reviews on the bioactive compounds from fungi, actinomycetes and other microbes [16,17], the amount of work done in the area is quite versatile, tenacious and significant. There is a need to comprehend these topics periodically to have its effective output for future research keeping in mind the probability of success of any newly discovered bioactive compound in clinical studies has been 0.01 to 1 % based on therapeutic area and type of scaffold. This demands that the base of such scaffolds in the ladder of clinical development should be wider. This width can be increased by exploring such less-tapped resources, the endophytic fungi.

In our previous review, we have covered antibacterials reported from endophytic fungi up to 2014 [1]. This review describes some bioactive molecules isolated from 2015 onwards to early 2021 from various endophytic fungi from terrestrial plants and designated as antibacterials. The antibacterial activity against various pathogenic organisms is listed in Table 1.

## 2. Antibacterials from Various Class of Endophytic Fungi

### 2.1. Ascomycetes

Ascomycetes are the fungi characterized by the formation of ascospores and some of the genera belonging to this class are known to produce chemically diverse metabolites. The important genera include *Diaporthe*, *Xylaria*, *Chaetomium*, *Talaromyces*, and *Paraphaeosphaeria* and are known to produce terpenoids, cytochalasins, mellein, alkaloids, polyketides, and aromatic compounds. Here we report the antibacterial from ascomycetes.

#### 2.1.1. *Diaporthe* (Asexual State: *Phomopsis*)

**Figure 1:**
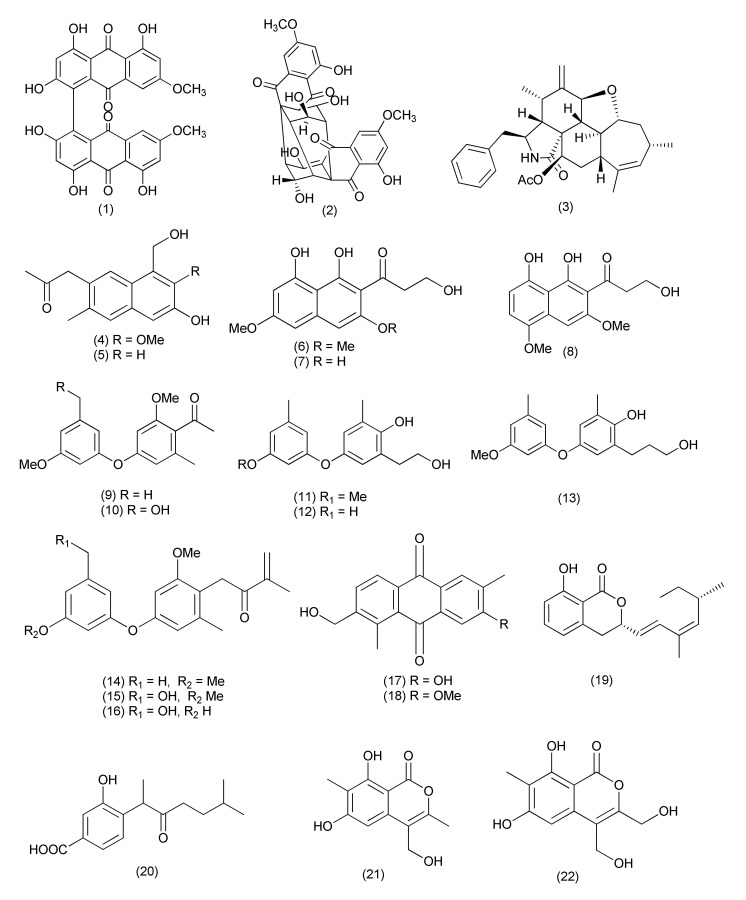
Structures of metabolites **1**–**22** isolated from Ascomycetes.

The genus *Diaporthe* (asexual state: *Phomopsis*) has been thoroughly investigated for secondary metabolites that have various pathogenic, endophytic and saprobic species of temperate and tropical habitats. Two natural bisanthraquinone, (+)-1,1′-bislunatin (bis) (**1**) and (+)-2,2′-epicytoskyrin A (epi) (**2**, Figure 1), were extracted from endophytic fungi, *Diaporthe* sp. GNBP-10 is associated with plant *Uncaria gambir*. Compounds (bis)-(**1**) and (epi)-(**2**) showed promising anti-tubercular activity, against *Mycobacterium tuberculosis* strains H37Rv (Mtb H37Rv) with MIC values of 0.422 and 0.844 μM, respectively. Both compounds have the ability to combat nutrient-starvation and biofilms of the Mtb model with relatively moderate activity in bacterial reduction with between 1–2 fold log reduction. Both compounds could reduce the number of Mtb infected into macrophages with 2-fold log reduction. The in-silico results via a docking study show that both compounds have a good affinity with pantothenate kinase (PanK) enzyme with a Glide score of −8.427 kcal/mol and −7.481 kcal/mol for the epi and bis compounds, respectively [18].

An endophytic fungus, *Diaporthe* sp. GDG-118, associated with *Sophora tonkinensis* collected from Hechi City (China) yielded a new compound 21-acetoxycytochalasin J3 (**3,** Figure 1) and inhibited the pathogens *Bacillus anthraci* and *E. coli* at 12.5 μg/mL concentration (6 mm sterile filter paper discs were impregnated with 20 µL (50 µg) of each compound) [19].

Two novel naphthalene derivatives, 1-(3-hydroxy-1-(hydroxymethyl)-2-methoxy-6-methylnaphthalen-7-yl) propan-2-one (**4**) and 1-(3-hydroxy-1-(hydroxymethyl)-6-methyl-naphthalen-7-yl)propan-2-one (**5**, Figure 1), were obtained from the *Phomopsis fukushii*. Compounds **4** and **5** displayed poor anti-methicillin-resistant *Staphylococcus aureus* (anti-MRSA) activity, with zones of inhibition of 10.2 and 11.3 mm, respectively (6 mm sterile filter paper discs were impregnated with 20 µL (50 µg) of each compound) [20].

Earlier *Phomopsis fukushii (Diaporthe fukushii)* isolated from the rhizome of *Paris polyphylla var. yunnanensis* was the source of three new compounds namely 3-hydroxy-1-(1,8- dihydroxy-3,6-dimethoxynaphthalen-2-yl)propan-1-one (**6**), 3-hydroxy-1-(1,3,8-trihydroxy-6-methoxynaphthalen-2-yl)propan-1-one (**7**) and 3-hydroxy-1-(1,8-dihydroxy3,5-dimethoxy naphthalen-2-yl) propan-1-one (**8**, Figure 1). Compounds **6**–**8** exhibited anti-MRSA-ZR11 activity, with MIC values of 8, 4, and 4 µg/mL, respectively [21]. Later two new di-Ph ethers, 1-[2-methoxy-4-(3-methoxy-5-methylphenoxy)-6-methylphenyl]-ethanone (**9**) and 1-[4-(3-(hydroxymethyl)-5-methoxyphenoxy)-2-methoxy-6-methylphenyl]-ethanone (**10**, Figure 1), were also purified from the same fungus. Compounds **9–10** exhibited anti-MRSA activity with good inhibition (zones of 13.8 and 14.6 mm, respectively) [22].

Three new di-Ph ethers, 4-(3-methoxy-5-methylphenoxy)-2-(2-hydroxyethyl)-6-methylphenol (**11**), 4-(3-hydroxy-5-methylphenoxy)-2-(2-hydroxyethyl)-6-methylphenol (**12**) and 4-(3-methoxy-5-methylphenoxy)-2-(3-hydroxypropyl)-6-methylphenol (**13**, Figure 1) were purified from *Phomopsis fukushii* associated with the rhizome of *Paris polyphylla* var. *yunnanensis*. Compounds **11–13**, exhibited potent anti-MRSA activity, with 20.2, 17.9 and 15.2 mm inhibition zones, respectively, when tested at 50 µg concentration in 6 mm discs [23].

*Phomopsis fukushii* isolated from the rhizome of *Paris polyphylla* var. *yunnanensis* yielded three new isopentylated diphenyl ethers, 1-(4-(3-methoxy-5-methylphenoxy)-2-methoxy-6-methylphenyl)-3-methylbut-3-en-2-one (**14**), 1-(4-(3-(hydroxymethyl)-5-methoxyphenoxy)-2-methoxy-6-methylphenyl)-3-methylbut-3-en-2-one (**15**) and 1-(4-(3-hydroxy-5-(hydroxymethyl) phenoxy)-2-methoxy-6-methylphenyl)-3-methylbut-3-en-2-one (**16**, Figure 1). Compounds **14–16** displayed anti-MRSA activity with 21.8, 16.8 and 15.6 mm inhibition zones, respectively (50 µg/6 mm disc) [24].

Two new anthraquinones, 3-hydroxy-6-hydroxymethyl-2,5-dimethylanthraquinone (**17**) and 6-hydroxymethyl-3-methoxy-2,5-dimethylanthraquinone (**18**, Figure 1), were purified from the endophytic fungus *Phomopsis* sp. and displayed good anti-MRSA activity with inhibition zone diameters (IZDs) of 14.2 and 14.8 mm, respectively [25].

A new dihydroisocoumarin derivative diaporone A (**19**, Figure 1), was purified from *Diaporthe sp*. an endophyte of *Pteroceltis tatarinowii*. Compound **19** showed MIC at 66.7 μM against *Bacillus subtilis* [26].

A pair of new phenolic bisabolane-type sesquiterpenoid enantiomers (±)-phomoterpenes A and B [(±)-1] (**20**) along with two new isocoumarins, phomoisocoumarins C-D (**21–22**, Figure 1) were purified from an endophytic fungus *Phomopsis prunorum* (F4-3). Compounds (+)-1 (**20** and **22**) exhibited average antimicrobial activity against *Pseudomonas syringae pv. lachrymans* with MIC values of 15.6 μg/mL, and compounds (−)-1 (**20** and **21**) displayed poor activity with MICs of 31.2 μg/mL each. Compounds (−)-1, (+)-1, (**20**, **21**, **22**) showed antibacterial activity against *Xanthomonas citri pv. phaseoli* var. *fuscans* with MIC values of 31.2, 62.4, 31.2, and 31.2 μg/mL, respectively [27].

**Figure 2:**
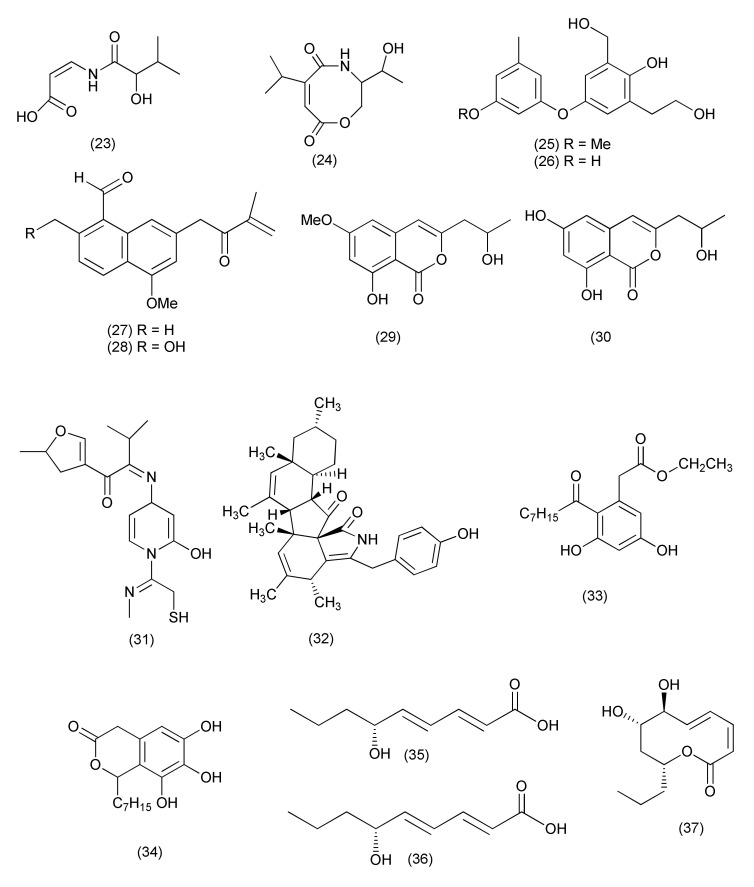
Structures of metabolites **23**–**37** isolated from Ascomycetes.

The fungus *Diporthe vochysiae* LGMF1583 isolated from *Vochysia divergens* yielded two new carboxamides, vochysiamides A (**23**), and B (**24**, Figure 2). Compound **24** inhibited *Klebsiella pneumoniae* carbapenemase-producing (KPC), MSSA, and MRSA with MIC of 0.08, 1.0, and 1.0 µg/mL, respectively, and compound **23** was active against KPC with a MIC of 1.0 μg/mL. KPC is of public health concern due to the presence of antimicrobial resistance carbapenemases [28].

An endophyte *Phomopsis asparagi* obtained from the rhizome of *Paris polyphylla* var. *yunnanensis* was the source of two new di-Ph ethers, 4-(3-methoxy-5-methylphenoxy)-2-(2-hydroxyethyl)- 6-(hydroxymethyl)phenol (**25**), and 4-(3-hydroxy-5-methylphenoxy)-2-(2-hydroxyethyl)-6-(hydroxymethyl)phenol (**26**, Figure 2). Compounds **25** and **26** exhibited potent anti-MRSA activity with 10.8 and 11.4 mm inhibition zones, respectively [29].

Two new naphthalene derivatives, 5-methoxy-2-methyl-7-(3-methyl-2-oxobut-3-enyl)-1-naphthaldehyde (**27**) and 2-(hydroxymethyl)-5-methoxy-7-(3-methyl-2-oxobut-3-enyl)-1-naphthaldehyde (**28**, Figure 2), were characterized from *Phomopsis* sp., an endophyte of *Paris polyphylla* var. *yunnanensis*. Compounds **27** and **28** displayed potent antibacterial activity with 14.5 and 15.2 mm zones of inhibition, respectively, against MRSA [30].

The endophytic fungus *Diaporthe terebinthifolii* LGMF907 associated with the plant *Schinus terebinthifolius* yielded diaporthin (**29**) and orthosporin (**30**, Figure 2). Compound **29** displayed antimicrobial activity against various pathogens like *E. coli*, *Micrococcus luteus,* MRSA, and *S. aureus* with 1.73, 2.47, 9.50, and 9.0 mm zones of inhibition, respectively at 100 μg/disk concentration. Compound **30** inhibited *E. coli*, *M. luteus*, MRSA, and *S. aureus* with 1.03, 1.53, 9.0 and 9.33 mm zones of inhibition, respectively, when tested at 100 μg/disk [31].

A pyrimidine iminomethylfuran derivative, (2*Z*)-2-(1,4-dihydro-2-hydroxy-1-((*E*)-2-mercapto-1-(methylimino)ethyl)pyrimidine-4-ylimino)-1-(4,5-dihydro-5-methylfuran-3-yl)-3-methylbutane-1-one (**31**, Figure 2) was extracted from *Phomopsis*/*Diaporthe* sp. GJJM 16 is associated with *Vitex negundo* and inhibited *S. aureus*, and *P. aeroginosa* with MICs of 1.25 μg/mL each [32].

*Phomopsis* sp. PSU-H188 associated with *Hevea brasiliensis*, yielded the known compounds diaporthalasin (**32**), cytosporones B (**33**) and cytosporones D (**34**, Figure 2). Compound **32**, displayed antibacterial activity against *S. aureus* and MRSA with equal MIC values of 4 μg/mL, but compound **33** inhibited *S. aureus* and MRSA with MIC values of 32 and 16 μg/mL, respectively. Compound **34** also inhibited *S. aureus* and MRSA with MIC values at higher concentrations of 64 and 32 μg/mL, respectively [33].

**Figure 3:**
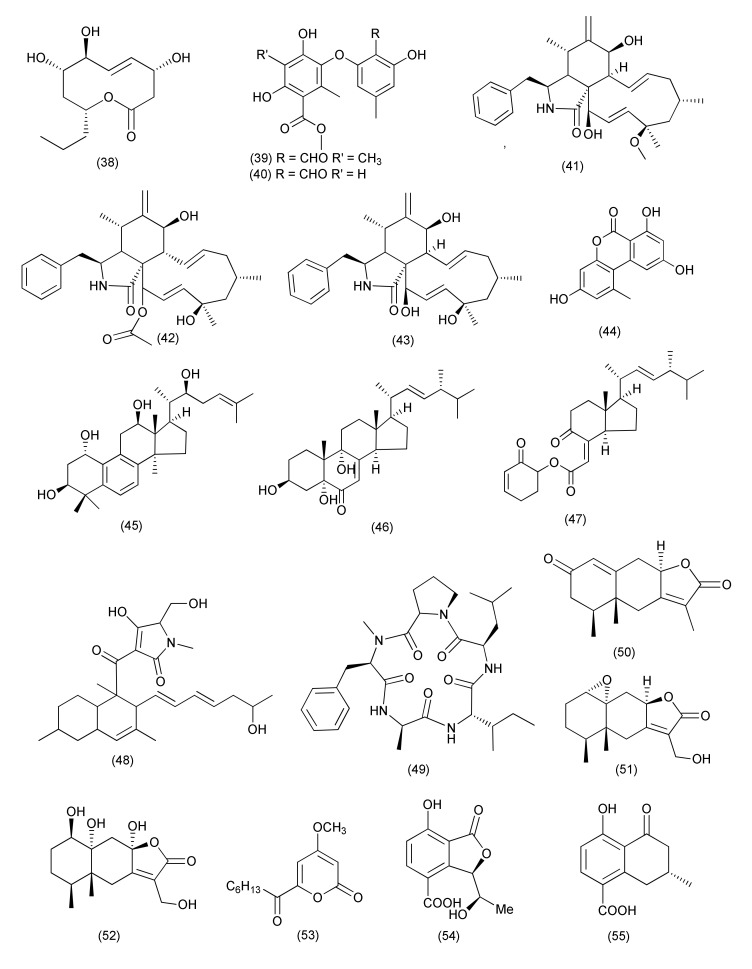
Structures of metabolites **38**–**55** isolated from Ascomycetes.

An endophyte, *Diaporthe terebinthifolii* GG3F6, associated with *Glycyrrhiza glabra* yielded two new hydroxylated unsaturated fatty acids namely diapolic acid A–B (**35–36**) and the known molecules xylarolide (**37**, Figure 2) and phomolide G (**38**, Figure 3). Compounds **35–38** inhibited *Yersinia enterocolitica* with an IC_50_ values of 78.4, 73.4, 72.1 and 69.2 μM, respectively [34].

The compounds phomosine A (**39**), and phomosine C (**40**, Figure 3), were obtained from *Diaporthe* sp. F2934 from *Siparuna gesnerioides*. Compound **39** was found to be active against *Bordetella bronchiseptica*, *Enterococcus faecalis*, *Enterococcus cloacae*, *S. aureus*, and *Streptococcus oralis* with 10, 10, 10, 12 and 9 mm inhibition zones at 4 µg/mL concentration, respectively. Compound **40** inhibited *S. aureus*, *M. luteus*, *S. oralis*, *E. faecalis*, *E. cloacae*, and *B. bronchiseptica*, with 9, 6, 8, 8, 8 and 9 mm inhibition zones at 4 µg/mL concentration, respectively [35].

Known cytochalasins 18-methoxycytochalasin J (**41**), cytochalasins H (**42**), J (**43**) and alternariol (**44**, Figure 3) were extracted from *Phomopsis* sp., residing inside *Garcinia kola* nuts. Compounds **41–44** were found to be active against *Shigella flexneri* (MIC, 128 μg/mL each). Compounds **41** and **42** showed activity against *S. aureus* with MIC values of 128 and 256 μg/mL, respectively [36].

The fungal culture *Diaporthe* sp. LG23, an endophyte of *Mahonia fortune*, yielded some new lanostanoids, 19-nor-lanosta-5(10),6,8,24-tetraene-1α,3β,12β,22S-tetraol (**45**), 3β,5α,9α-trihydroxy-(22E,24R)-ergosta-7,22-dien-6-one (**46**), and chaxine C (**47**, Figure 3). Compound **45** was found to be active against *S. aureus*, *E. coli*, *B. subtilis*, *Pseudomonas aeruginosa*, and *Streptococcus pyogenes*, with MIC values of 5.0, 5.0, 2.0, 2.0 and 0.1 µg/mL, respectively. Compounds **46** and **47** were active against *B. subtilis* with MIC values of 5.0 µg/mL each [37].

The known compound, pyrrolocin A (**48**, Figure 3), was purified from *Diaporthales* sp. E6927E isolated from *Ficus sphenophyllum*. Pyrrolocin A (**48**) displayed inhibition against *S. aureus* and *E. faecalis* with MICs of 4 and 5 µg/mL, respectively [38].

#### 2.1.2. *Xylaria*

The genus *Xylaria* comprises various endophytic species associated with both vascular and nonvascular plants. For example, ellisiiamide A (**49**, Figure 3) was isolated from *Xylaria ellisii* from *Vaccinium angustifolium* and was chemically characterized using 1D and 2D NMR, HRMS/MS data. It showed modest inhibitory activity against *E. coli* (MIC, 100 μg/mL) [39].

Xylareremophil (**50**), a new eremophilane sesquiterpene, along with the already reported eremophilanes mairetolides B (**51**) and G (**52**, Figure 3) were extracted from *Xylaria* sp. GDG-102 residing inside *S. tonkinensis*. Compound **50** displayed moderate activity against *Proteus vulgaris* and *Micrococcus luteus* (MIC, of 25 μg/mL each). Compound **51** was found to be active against *M. luteus*, with a MIC value of 50 μg/mL. Compound **52** inhibited *P. vulgaris* with a MIC value of 25 μg/mL and *M. luteus* with a MIC value of 50 μg/mL. Compounds **50–52** also displayed inhibition of *B. subtilis* and *Micrococcus lysodeikticus* with MIC values of 100 μg/mL, respectively [40].

A new compound, 6-heptanoyl-4-methoxy-2H-pyran-2-one (**53**, Figure 3), was purified from *Xylaria* sp. (GDG-102) an endophyte of *S. tonkinensis* and displayed antibacterial activity against *E. coli* as well as *S. aureus* (MIC, 50 μg/mL) [41].

**Figure 4:**
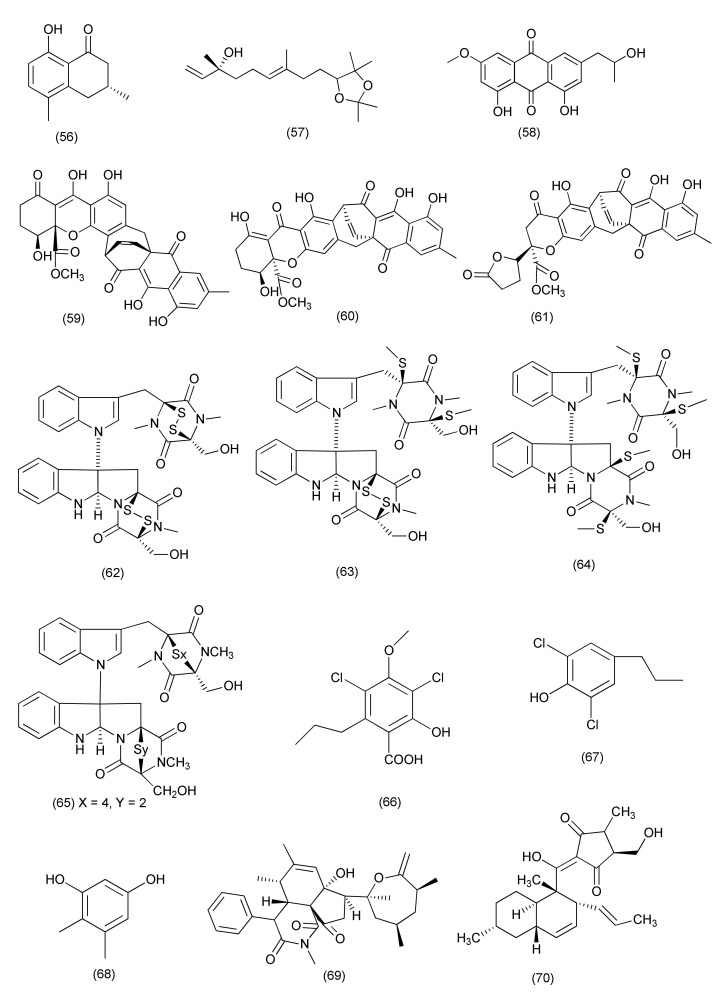
Structures of metabolites **56**–**70** isolated from Ascomycetes.

The phthalide derivative xylarphthalide A (**54**) and known compounds (−)-5-carboxylmellein (**55**, Figure 3) and (−)-5-methylmellein (**56**, Figure 4) were extracted from *Xylaria* sp. (GDG-102) associated with *S. tonkinensis*. Compound **54** inhibited *Bacillus anthracis*, *B. megaterium*, *B. subtilis*, *S. aureus*, *E. coli*, *Shigella dysenteriae* and *Salmonella paratyphi*, with the MICs of 50, 25, 12.5, 25, 12.5, 25 and 25 μg/mL, respectively. Compound **55** showed antibacterial activity with MIC of values of 25, 25, 12.5, 25, 25, 25 and 25 μg/mL against *B. anthracis*, *B. megaterium*, *B. subtilis*, *S. aureus*, *E. coli*, *S. dysenteriae* and *S. paratyphi*, respectively. Compound **56** displayed antibacterial activity with MIC values of 25, 12.5, 12.5, 25, 25, and 50 μg/mL against *B. megaterium*, *B. subtilis*, *S. aureus*, *E. coli*, *S. dysenteriae* and *S. paratyphi*, respectively [42].

A novel compound 3,7-dimethyl-9-(-2,2,5,5-tetramethyl-1,3-dioxolan-4-yl)nona-1,6-dien-3-ol (**57**), and previously reported compound nalgiovensin (**58**, Figure 4) were purified from *Xylaria* sp., associated with *Taxus mairei*. Compound **57** exhibited strong inhibition against *B. subtilis* (48.1%), *B. pumilus* (31.6%) and *S. aureus* (47.1%). Compound **58** exhibited broad inhibition against *S. aureus* (42.1%), *B. subtilis* (36.8%), *B. pumilus* (47.1%) and *E. coli* (41.2%) [43].

#### 2.1.3. *Chaetomium*

The genus *Chaetomium* has been included among the genera producing various bioactive compounds and more than 200 secondary metabolites belonging to diverse structural types such as anthraquinones, azaphilones, chaetoglobosins, chromones, depsidones, epipolythiodioxopiperazines, terpenoids, and steroids and xanthones have beenrecorded, making it a rich source of novel bioactive metabolites. Most of these fungal metabolites exhibited antitumor, cytotoxic, antimalarial, enzyme inhibitory, antibiotic, and other activities [44]. Here we report the antibacterial compounds isolated from the genus *Chaetomium*.

A new xanthoquinodin B9 (**59**), along with previously reported two xanthoquinodins, xanthoquinodin A1 (**60**) and xanthoquinodin A3 (**61**), and three epipolythio- dioxopiperazines, chetomin (**62**), chaetocochin C (**63**) and dethiotetra(methylthio)chetomin (**64**, Figure 4), were obtained from *C. globosum* 7s-1, associated with *Rhapis cochinchinensis*. Xanthoquinodins **59–61** displayed potent antibacterial activity, with MIC values of 0.87, 0.44 and 0.22 μM against *B. cereus*, respectively. Compounds **59–61** were also found active against *S. aureus* and MRSA (MICs in the range of 0.87 to 1.75 μM). Epipolythiodioxopiperazines **62–64** exhibited potent activity against *B. cereus*, *S. aureus*, and MRSA (MICs in the range of 0.02 pM to 10.81 mM). Compound **62** showed the highest activity towards *B. cereus*, *S. aureus* and MRSA (MICs of 0.35 μM, 10.74 and 0.02 pM). Compounds **59–64** showed poor activity against *E. coli*, *P. aeruginosa*, and *Salmonella typhimurium* (MICs of 45.06 to >223.72 μM). Epipolythiodioxopiperazines **62–64** showed activity against *Mycobacterium tuberculosis* with MICs of 0.55, 4.06 and 8.11 μM, respectively [45].

Known compounds chaetocochin C (**63**), chetomin A (**65**) and chetomin (**62**, Figure 4) were extracted from *Chaetomium* sp. SYP-F7950 residing inside *Panax notoginseng*. Compounds **62**, **63** and **65** displayed potent activity against *B. subtilis*, *S. aureus*, and *Enterococcus faecium*, with MIC values ranging from 0.12 to 19.3 μg/mL. The length of *B. subtilis* was increased up to 1.8-fold after treatment with compounds **62**, **63** and **65**. These compounds also showed good interactions with the filamentous temperature-sensitive protein Z (FtsZ) of *B. subtilis* in an in silico molecular docking study. These results revealed that inhibition of pathogenic *B. subtilis* could be achieved by combination with FtsZ and inhibition of cell division [46].

Compounds differanisole A (**66**), 2,6-dichloro-4-propylphenol (**67**) and 4,5-dimethylresorcinol (**68**, Figure 4), were purified from *Chaetomium* sp. HQ-1, isolated from *Astragalus chinensis*. Compounds **66–68** displayed average activity against *Listeria monocytogenes*, *S. aureus*, and MRSA (MICs ranging from 16 to 128 μg/mL). Compound **66** showed a MIC of 16 μg/mL for *L. monocytogenes* and a MIC of 128 μg/mL for *S. aureus* and MRSA. Compounds **67** and **68** could suppress the growth of *L. monocytogenes* with MICs of 64 and 32 μg/mL, respectively [47].

A novel cytochalasan, chamiside A (**69**, Figure 4), was obtained from *Chaetomium nigricolor* F5, an endophytic fungus associated with *Mahonia fortune* collected from Qingdao (China) and showed inhibition of *S. aureus* with a MIC of 25 μg/mL [48].

A known compound, equisetin (**70**, Figure 4), was purified from *C. globosum* of *Salvia miltiorrhiza*. Compound **70** displayed activity against multidrug-resistant *E. faecalis*, *E. faecium*, *S. aureus*, and *S. epidermidis* with MIC values of 3.13, 6.25, 3.13, and 6.25 μg/mL, respectively [49].

**Figure 5:**
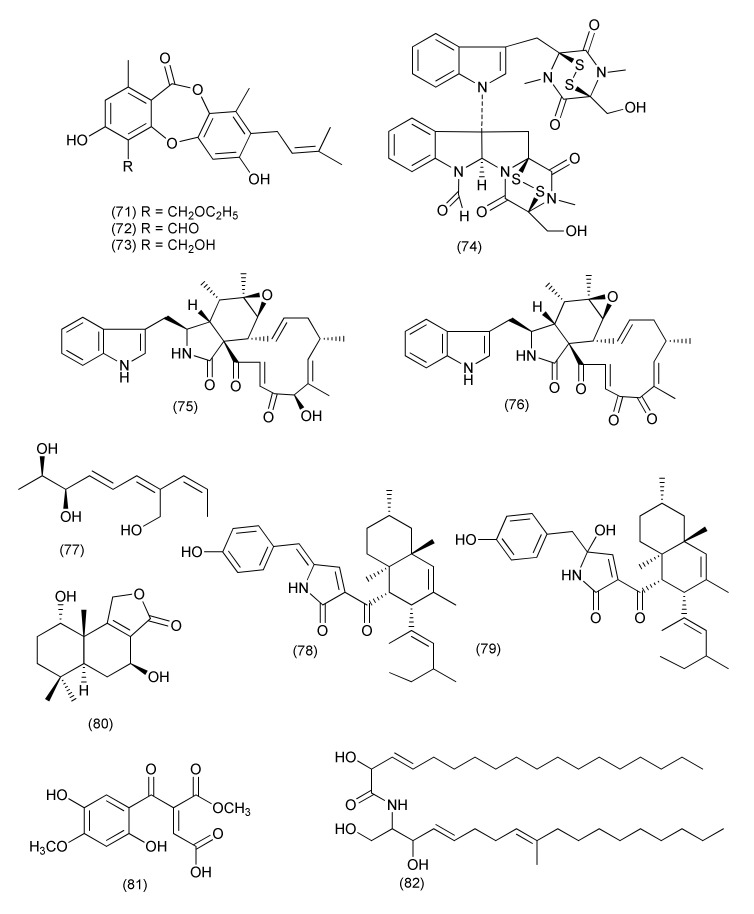
Structures of metabolites **71**–**82** isolated from Ascomycetes.

*Chaetomium* sp. Eef-10, from *Eucalyptus exserta* yielded a new depsidone mollicellin O (**71**), along with the known compounds mollicellin H (**72**) and mollicellin I (**73**, Figure 5). Mollicellin H (**72**) displayed potent activity against *S. aureus* and *S. aureus* N50, with IC_50_ values of 5.14 and 6.21 μg/mL, respectively. Mollicellin O (**71**) exhibited antibacterial activities against *S. aureus* and *S. aureus* N50, with IC_50_ values of 79.44 and 76.35 μg/mL, respectively, while mollicellin I (**73**) exhibited activity against *S. aureus* and *S. aureus* N50 with IC_50_ values of 70.14 and 63.15 μg/mL, respectively [50].

A new compound, 6-formamidochetomin (**74**, Figure 5) was isolated from *Chaetomium* sp. M336 an endophyte of *Huperzia serrata*. Compound **74** inhibited *E. coli*, *S. aureus*, *S. typhimurium* and *E. faecalis* with MIC values of 0.78 μg/mL [51].

Two known cytochalasans, chaetoglobosin A (**75**) and C (**76**, Figure 5), were purified from *Chaetomium globosum,* an endophyte of *Nymphaea nouchali*. Compound **75** inhibited *B. subtilis*, *S. aureus*, and MRSA with MIC values of 16, 32 and 32 μg/mL, respectively, and the MIC values for compound **76** were >64 μg/mL for all the microorganisms tested [52].

#### 2.1.4. *Talaromyces*

An endophytic fungus *Talaromyces pinophilus* XL-1193 residing inside the plant *Salvia miltiorrhiza* yielded a new polyene, pinophol A (**77**, Figure 5). Pinophol A (**77**) exhibited low activity against *Bacterium paratyphosum* B with a MIC value of 50 μg/mL [53].

The compounds talaroconvolutin A (**78**) and talaroconvolutin B (**79**, Figure 5), were discovered in *Talaromyces purpureogenus* XL-25, an endophyte associated with *Panax notoginseng*. Compound **78** showed pronounced activity against *B. subtilis* (MIC, 1.56 μM). Compound **79** had a certain inhibitory activity against *Micrococcus lysodeikticus* (MIC = 0.73 μM) and *Vibrio parahaemolyticus* (MIC = 0.18 μM) [54].

A drimane sesquiterpenoid (1*S*,5*S*,7*S*,10*S*)-dihydroxyconfertifolin (**80**, Figure 5) was purified from *Talaromyces purpureogenus* residing inside the plant *Panax notoginseng*. Compound **80** inhibited *E. coli* with a MIC value of 25 μM/L [55].

**Figure 6:**
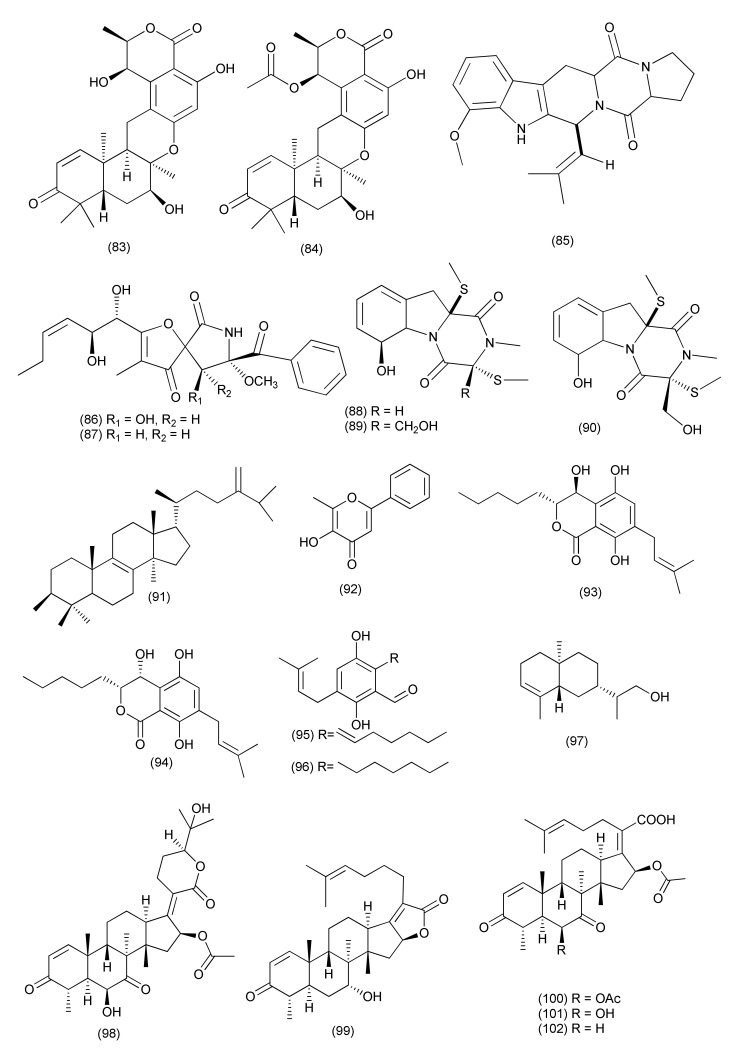
Structures of metabolites **83**–**102** isolated from Ascomycetes.

A novel polyketide, talafun (**81**), and a new compound, N-(2′-hydroxy-3′-octadecenoyl)-9-methyl-4,8-sphingadienin (**82**, Figure 5), were purified from *Talaromyces funiculosus* -Salicorn 58 together with some previously reported compounds, chrodrimanin A (**83)**, and chrodrimanin B (**84**, Figure 6). Compound **81** exhibited potent activity against *E. coli* (MIC, 18 μM) but poor activity toward *S. aureus* (MIC, 93 μM). Compound **82** was found to be active against *Mycobacterium smegmatis*, *S. aureus*, *Micrococcus tetragenus*, and *E. coli*, with MIC values of 85, 90, 24, and 68, 93 μM, respectively. Compound **83** inhibited *S. aureus*, *M. tetragenus*, *Mycobacterium phlei*, and *E. coli* (MICs of 67, 28, 47, and 26 μM). However, compound **84** showed only moderate activity against *E. coli* with a MIC of 43 μM [56].

Alkaloids **85–90** (Figure 6), were extracted from *Talaromyces* sp. LGT-2, from *Tripterygium wilfordii*. Compounds **85–90** inhibited *E. coli*, *P. aeruginosa*, *S. aureus*, *Bacillus licheniformis*, and *Streptococcus pneumoniae*, with MIC values in the range of 0.125 to 1.0 50 μg/mL [57].

#### 2.1.5. Minor Taxa of the *Ascomycetes*

The known compound euphorbol (**91**, Figure 6) was isolated from *Rhytidhysteron* sp. BZM-9, an endophyte isolated from the leaves of *Leptospermum brachyandrum*. Compound **91** displayed weak antibacterial activity against MRSA, with a MIC value of 62.5 μg/mL (positive control vancomycin MIC 1.25 μg/mL) [58].

A new natural product, stagonosporopsin C (**92**, Figure 6) was purified from an endophytic fungus, *Stagonosporopsis oculihominis*, isolated from *Dendrobium huoshanense*. Stagonosporopsin C (**92**) exhibited moderate inhibitory activity against *S. aureus* sub sp. *aureus* ATCC29213 with a MIC_50_ value of 41.3 μM (positive control penicillin G, MIC_50_ value 1.963 μM) [59].

Two new compounds eutyscoparols H-I (**93**, **94**) together with the related known ones tetrahydroauroglaucin (**95**) and flavoglaucin (**96**, Figure 6), were isolated from the endophytic fungus *Eutypella scoparia* SCBG-8. Compounds **93–96** displayed growth inhibition against *S. aureus* and MRSA, with MIC values ranging from 1.25 to 6.25 μg/mL [60].

A new sesquiterpene eutyscoparin G (**97**, Figure 6) was purified from an endophytic fungus *Eutypella scoparia* SCBG-8 isolated from leaves of *Leptospermum brachyandrum* from the South China Botanical Garden (SCBG, Chinese Academy of Sciences, Guangzhou, China). Compound **97** exhibited antibacterial activity against *S. aureus* and MRSA with MIC values of 6.3 μg/mL [61].

**Figure 7:**
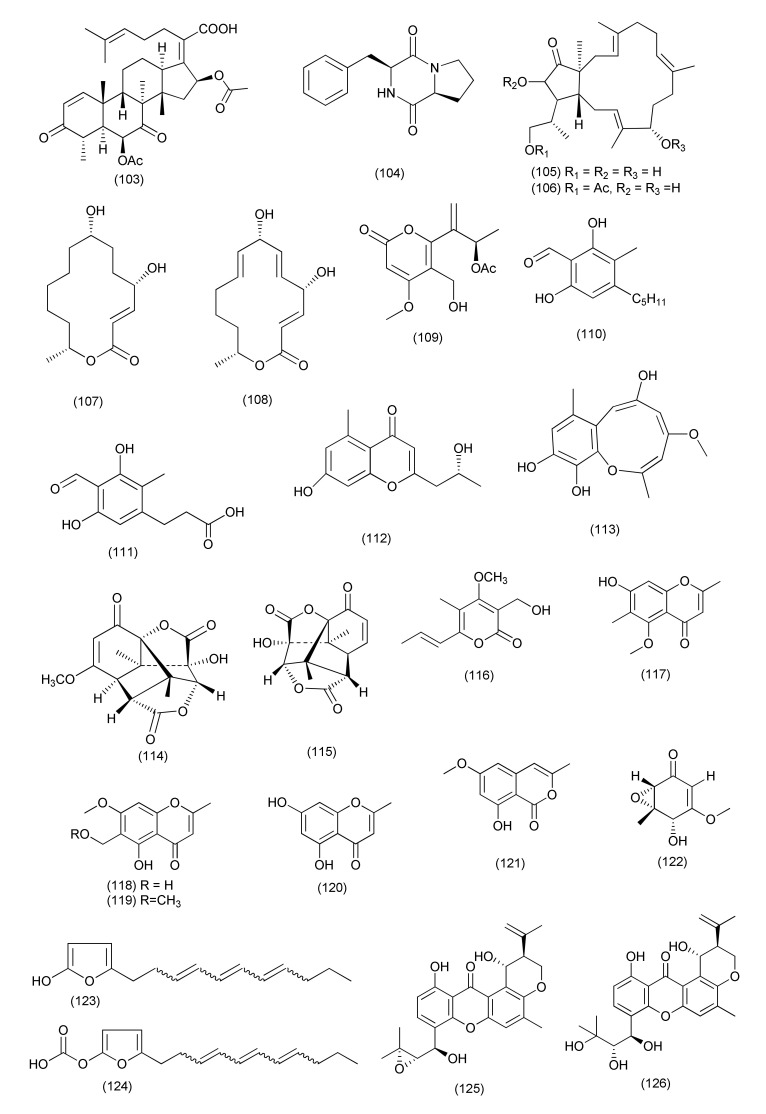
Structures of metabolites **103**–**126** isolated from Ascomycetes.

Two new helvolic acid derivatives named sarocladilactone A (**98**), sarocladilactone B (**99**), along with the previously reported compounds helvolic acid (**100**), helvolinic acid (**101**), 6-desacetoxyhelvolic acid (**102**, Figure 6), and 1,2-dihydrohelvolic acid (**103**, Figure 7), were isolated from *Sarocladium oryzae* DX-THL3, associated with leaves of *Oryza rufipogon* Griff. Compounds **98–103** showed antibacterial activity against *S. aureus* with MIC values of 64, 4, 8, 1, 4 and 16 μg/mL, respectively (positive control tobramycin MIC 1 μg/mL), while compound **101** also showed antibacterial activity against *B. subtilis* with a MIC value of 64 μg/mL (positive control tobramycin, MIC 64 μg/mL). Compounds **98**, **101**, **103**, showed some potent antibacterial activity against *E. coli* with MIC 64 μg/mL [62].

The diketopiperazine cyclo(L-Pro-L-Phe) (**104**, Figure 7), was purified from *Paraphaeosphaeria sporulosa*, associated with *Fragaria x ananassa*. Compound **104** displayed activity against *Salmonella strains*, S1 and S2, with IC_50_ values of 7.2 and 7.9 μg/mL and MICs of 71.3 and 78.6 μg/mL, respectively [63].

A fungal culture of *Aplosporella javeedii* isolated from *Orychophragmus violaceus* was the source of terpestacin (**105**) fusaproliferin (**106)**, 6,7,9,10-tetrahydromutolide (**107**) and mutolide (**108**, Figure 7). Compounds **105**, **106**, **108** showed poor activities against *M. tuberculosis* H37Rv and compound **107** against *S. aureus*, respectively, with MICs of 100 μM [64].

A new chlamydosporol derivative pleospyrone E (**109**, Figure 7), was extracted from *Pleosporales* sp. Sigrf05, residing inside the tuberous roots of *Siraitia grosvenorii*. Compound **109** exhibited weak inhibition against *Agrobacterium tumefaciens*, *B. subtilis*, *R. solanacearum*, and *X. vesicatoria* with the same MIC value of 100.0 µM [65].

New polyketides aplojaveediins A and F (**110, 111**, Figure 7) were purified from the *Aplosporella javeedii* associated with the *Orychophragmus violaceus*. Compound **110** exhibited average activity against the sensitive *Staphylococcus aureus* strain ATCC 29213, the methicillin-resistant and vancomycin-intermediate sensitive (MRSA/VISA) *S. aureus* strain ATCC 700699 and *B. subtilis* (ATCC 169) with MICs of 50, 50 and 25 μM, respectively. Compound **111** also exhibited moderate inhibition against *S. aureus* ATCC 29213 and ATCC 700699 with MICs of 25 and 50 μM, respectively [66].

A new chromone, lawsozaheer (**112**, Figure 7), was isolated from *Paecilomyces variotii* from *Lawsonia alba*. Compound **112** showed activity against *S. aureus* (NCTC 6571) with 84.26% inhibition at 150 μg/mL [67].

A known polyketide, setosol (**113**, Figure 7), was extracted from an endophytic fungus *Preussia isomera* in *Panax notoginseng* from Wenshan, by using an OSMAC strategy. Compound **113** displayed potent activity against multidrug-resistant *E. faecium*, methicinllin-resistant *S. aureus* and multidrug-resistant *E. faecalis* with MIC values of 25 μg/mL [68].

A pair of enantiomeric norsesquiterpenoids, (+)- (**114**) and (−)-preuisolactone A (**115**, Figure 7) featuring an unprecedented tricyclo[4.4.01,6.02,8]decane carbon scaffold were isolated from *Preussia isomera*. XL-1326, obtained from the stems of *Panax notoginseng*. Compounds (+)-I and (−)-II are 2 rare naturally occurring sesquiterpenoidal enantiomers. Compounds **114** and **115** exhibited potent antibacterial activity against *Micrococcus luteus* and *B. megaterium* with MIC values of 10.2 and 163.4 μM, respectively [69].

A new α-pyrone derivative, udagawanone A (**116**, Figure 7) was isolated from *Neurospora udagawae* associated with *Quercus macranthera*, and displayed moderate inhibition against *S. aureus* (MIC = 66 μg/mL) [70].

Five chromone derivatives, including 2,6-dimethyl-5-methoxy-7-hydroxychromone (**117**), 6-hydroxymethyleugenin (**118**), 6-methoxymethyleugenin (**119**), and isoeugenitol (**120**), and isocoumarin congeners, 8-hydroxy-6-methoxy-3-methylisocoumarin (**121**, Figure 7) and diaporthin (**29**), were purified from *Xylomelasma* sp. Samif07, an endophyte of *Salvia miltiorrhiza*. Compound **120** showed good activity against *M. tuberculosis* (MIC 10.31 μg/mL). Compounds **29**, **117**–**121** displayed inhibitory activities against *B. subtilis*, *Staphylococcus haemolyticus*, *A. tumefaciens*, *Erwinia carotovora*, and *X. vesicatoria* (with MICs ranging from 25 ~ 100 μg/mL). Compounds **117** and **29** showed inhibition against only *E. carotovora* (MIC, 100 μg/mL), and *B. subtilis* (MIC, 50 μg/mL), respectively. Compounds **118**, **119**, **29** were found active against *S. haemolyticus* and *E. carotovora* (MIC of 75 μg/mL), whereas compound **121** exhibited stronger inhibition against *B. subtilis*, *A. tumefaciens*, and *X. vesicatoria*, with MICs of 25, 75, and 25 μg/mL, respectively [71].

The compound (4*S*,5*S*,6*S*)-5,6-epoxy-4-hydroxy-3-methoxy-5-methylcyclohex-2-en-1-one (**122**, Figure 7) was purified from *Amphirosellinia nigrospora* JS-1675, an endophytic fungus isolated from the stem tissue of *Pteris cretica*. Compound **122** showed high to moderate in vitro antibacterial activity, with MIC values ranging between 31.2 and 500 µg mL^−1^ against *Pectobacterium carotovorum* subsp. *Carotovorum*, *Agrobacterium konjaci*, *Burkholderia glumae*, *Clavibacter michiganensis* subsp. *michiganensis*, *A. tumefaciens*, *Pectobacterium chrysanthemi*, *R. solanacearum*, *Acidovorax avenae* subsp. *cattlyae*, *Xanthomonas arboricola* pv. *pruni*, *X. euvesicatoria*, *X. axonopodis* pv. *Citri*, *X. oryzae* pv. *oryzae* [72].

Two new alkylated furan derivatives, 5-(undeca-3′,5′,7′-trien-1′-yl)furan-2-ol (**123**) and 5-(undeca-3′,5′,7′-trien-1′-yl)furan-2-carbonate (**124**, Figure 7), were isolated from *Emericella* sp. XL029, an endophyte of *Panax notoginseng*. Compounds **123**, **124** inhibited *B. subtilis*, *B. cereus*, *S. aureus*, *B. paratyphosum B*, *S. typhi*, *P. aeruginosa*, *E. coli*, and *E. aerogenes* with MIC values ranging from 6.3 to 50 μg/mL [73].

**Figure 8:**
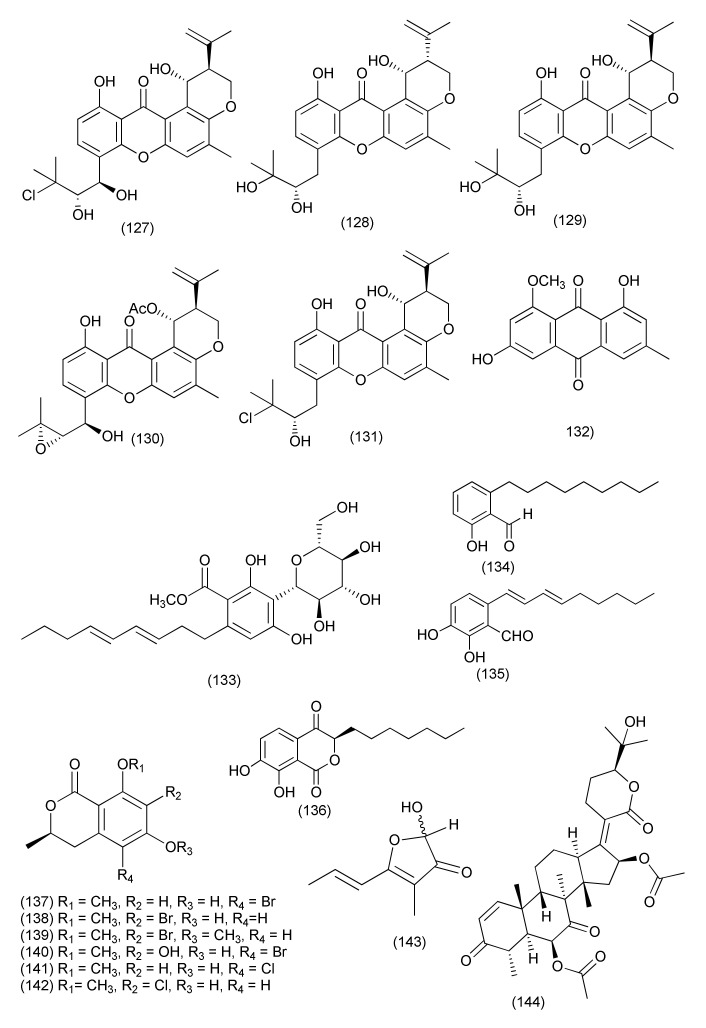
Structures of metabolites **127**–**144** isolated from Ascomycetes.

Four new compounds, 14-hydroxytajixanthone (**125**), 14-hydroxyltajixanthone hydrate (**126**, Figure 7), 14-hydroxy-15-chlorotajixanthone hydrate (**127**) and epitajixanthone hydrate (**128**), along with known compounds tajixanthone hydrate (**129**), 14-methoxyltajixanthone-25-acetate (**130**), and 15-chlorotajixanthone hydrate (**131**), questin (**132**) and carnemycin B (**133**, Figure 8), were purified from *Emericella* sp. XL029 residing inside the leaves of *Panax notoginseng*. Compounds **125**–**127**, **130**, **132**, **133** exhibited potent activity against *M. luteus*, *S. aureus*, *B. megaterium*, *B. anthracis*, and *B. paratyphosum* B (MIC values ranging from 12.5 and 25 μg/mL). Compound **128** exhibited potent activity against *M. luteus*, *S. aureus*, *B. megaterium*, and *B. paratyphosum* B (MIC 25 μg/mL each), while compounds **129, 131** inhibited *S. aureus*, *B. megaterium*, and *B. paratyphosum* B (MIC 25 and 12.5 μg/mL). Compounds **125**, **128**, **133** displayed average activity against drug-resistant *S. aureus* (MICs 50 μg/mL each). All isolated compounds **125**–**133** displayed moderate activity against *P. aeruginosa*, *E. coli*, and *E. aerogenes* (MIC 50 μg/mL) [74].

An endophytic fungus *Byssochlamys spectabilis* from the plant *Edgeworthia chrysantha* yielded bysspectin C (**134**, Figure 8) which was active against *E. coli* and *S. aureus* with MIC values of 32 and 64 µg/mL, respectively [75].

Two new compounds, sydowianumols A (**135**), and B (**136**, Figure 8), were isolated from *Poculum pseudosydowianum* (TNS-F-57853), an endophytic fungus associated with the petiole of *Quercus crispula* var. *crispula* in Yoshiwa. Compounds **135** and **136** exhibited anti-MRSA activity, with MIC_90_ values of 12.5 μg/mL [76].

Six previously undescribed halogenated dihydroisocoumarins, palmaerones A–C, (**137**–**139**) and E–G (**140**–**142**, Figure 8) were purified from *Lachnum palmae*, an endophytic fungus from *Przewalskia tangutica* by exposure to a histone deacetylase inhibitor SAHA. Compounds **137**, **138**, **140**–**142** were active against *B. subtilis*, with MIC values of 35, 30, 10, 50, and 55 μg/mL, respectively, while compounds **137–140**, were found active against *S. aureus* with MIC values of 65, 55, 60, and 55 μg/mL, respectively [77].

The polyketide nemanifuranone A (**143**), a nordammarane triterpenoid, was isolated from *Nemania serpens*, an endophyte of *Vitis vinifera*. Additionally, a known metabolite **144**, also a nordammarane triterpenoid (Figure 8) was isolated from the mycelium. Nemanifuranone A (**143**) showed modest activity against *E. coli*, with a MIC of 200 μg/mL, and significant inhibition (>75% inhibition) against *S. aureus*, *B. subtilis* and *M. luteus* at a concentration of 100–200 μg/mL. However, **144** showed significant inhibition (>75% inhibition) of *M. luteus* at a concentration of 100 μg/mL [78].

**Figure 9:**
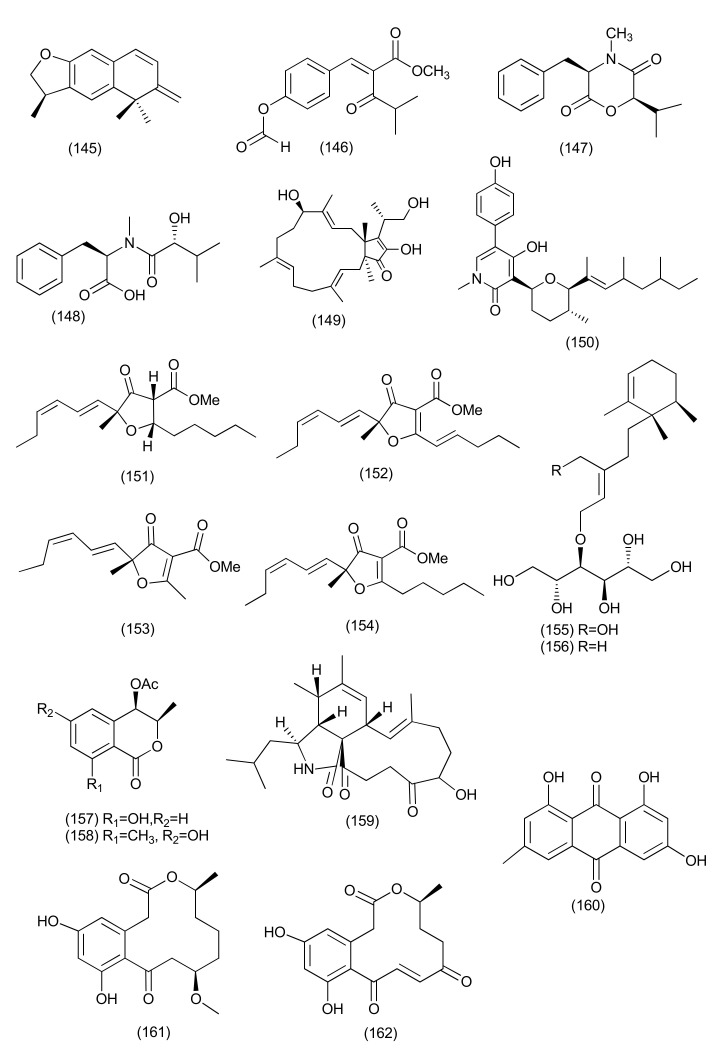
Structures of metabolites **145**–**158** and **159**–**162** isolated from Ascomycetes and Anamorphic Ascomycetes, respectively.

A sesquiterpene, variabilone (**145**, Figure 9), with a new skeleton, was isolated from the endophytic fungus *Paraconiothyrium variabile* isolated from *Cephalotaxus harringtonia*. Compound **145** behaved as a potent growth inhibitor of *B. subtilis* at an IC_50_ of 2.13 μg/mL after 24 h [79].

A new 4-hydroxycinnamic acid derivative compound, methyl 2-{(*E*)-2-[4-(formyloxy)phenyl]ethenyl}-4-methyl-3-oxopentanoate (**146**), along with the known compounds (3*R*,6*R*)-4-methyl-6-(1-methylethyl)-3-phenylmethylperhydro-1,4-oxazine-2,5-dione (**147**), (3*R*,6*R*)-N-methyl-N-(1-hydroxy-2-methylpropyl)-phenylalanine (**148**), siccanol (**149**), sambutoxin (**150**, Figure 9) and fusaproliferin (**106**), were extracted from *Pyronema* sp. an endophyte of the *Taxus mairei*. Compounds **106**, **146**–**150** also exhibited potential inhibitory activity, with IC_50_s of 64, 59, 57, 84, 43 and 32 μM against *Mycobacterium marinum*, respectively [80].

Three new natural furanones, pulvinulin A (**151**), graminin C (**152**), and *cis*-gregatin B (**153**), together with the known fungal metabolite, graminin B (**154**, Figure 9), were isolated from *Pulvinula* sp. 11120, an endophyte of the leaves of *Cupressus arizonica*. Compounds **151–154** displayed antibacterial against *E. coli* with 12, 18, 16, and 14 mm zones of inhibition [81].

Stelliosphaerols A (**155**) and B (**156**, Figure 9), new sesquiterpene−polyol conjugates were purified from a *Stelliosphaera formicum* endophytic fungus associated with the plant *Duroia hirsuta*. Compounds **155** and **156** inhibited *S. aureus* with MIC values of 250 μg/mL [82].

Two novel polyketides, *cis*-4-acetoxyoxymellein (**157**) and 8-deoxy-6-hydroxy-*cis*-4-acetoxyoxymellein (**158**, Figure 9) were extracted from an unidentified ascomycete, associated with *Melilotus dentatus*. Compound **157** was found to be active against *E. coli* and *B. megaterium* with 10 and 10 (partial inhibition) zones of inhibition at 0.05 mg concentration. Compound **158** displayed antibacterial activity against *E. coli* and *B. megaterium* with 9 and 9 (partial inhibition) zones of inhibition at a concentration of 0.05 mg [83].

### 2.2. Anamorphic Ascomycetes

Anamorphic Ascomycetes are the fungi that are the asexual form of ascomycetes. The first antibiotic penicillin-producing fungi belonged to this group. Fungi belonging to this group are prolific producers of bioactives metabolites. After the discovery of penicillin, this group is extensively screened for bioactives. Some important genera in this group are *Penicillium*, *Aspergillus*, *Fusarium*, *Pestalotiopsis*, *Phoma* and *Colletotrichum.* Here we report the antibacterials compounds from this group of fungi.

#### 2.2.1. *Aspergillus*

*Aspergillus* is one of the important fungal genera and some of the antibacterials from this genus such as aspochalasin P (**159**), alatinone (**160**), β-11-methoxycurvularine (**161**), and 12-keto-10,11-dehydrocurvularine (**162**, Figure 9) were purified from *Aspergillus* sp. FT1307 associated with plant *Heliotropium* sp. Compounds **159–162** showed weak activity against *Staphylococcus aureus* ATCC12600, *Bacillus subtilis* ATCC6633 and MRSA ATCC43300 with MICs in the range of 40 to 80 μg/mL [84].

**Figure 10:**
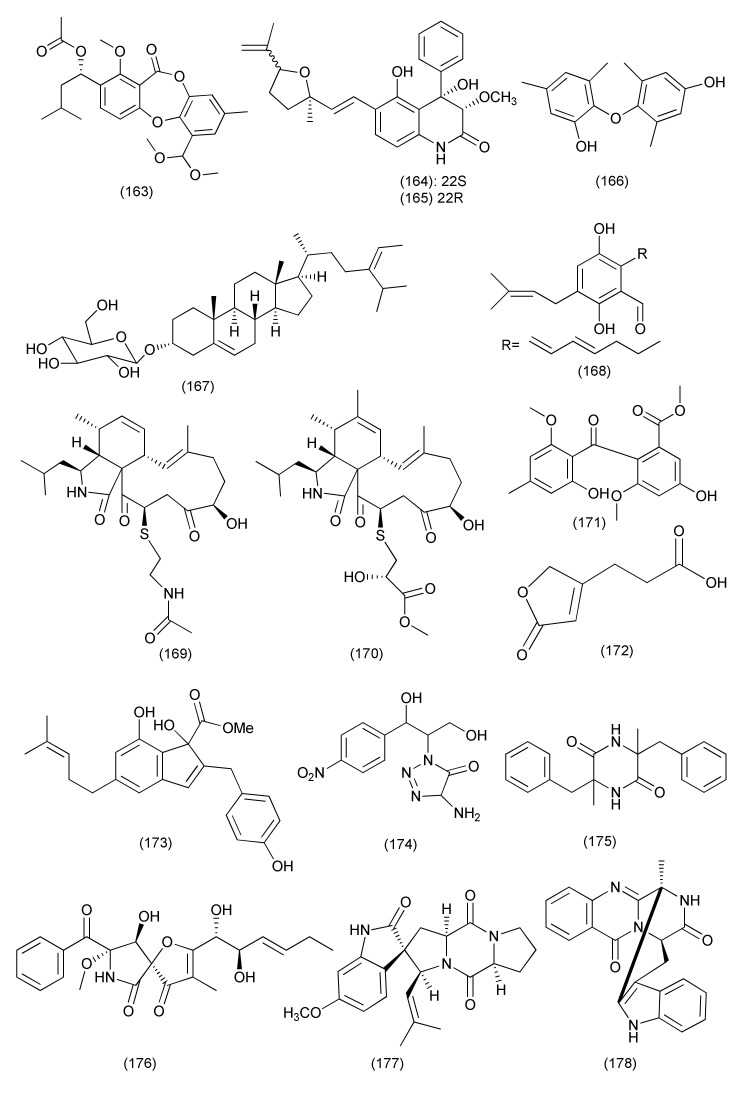
Structures of metabolites **163**–**178** isolated from Anamorphic Ascomycetes.

A new polyketide, aspergillone A (**163**, Figure 10), was isolated from *Aspergillus cristatus* associated with *Pinellia ternata*. Aspergilline A (**163**) is the first example of a bicyclo[2.2.2] diazaoctane indole alkaloid where the diketopiperazine structure is constructed from tryptophan and alanine. Aspergillone A (**163**) exhibited average antibacterial activities against *B. subtilis* and *S. aureus*, with MIC_50_ values of 8.5 and 32.2 μg/mL, respectively [85].

A new quinolone derivative, (22*S*)-aniduquinolone A (**164**) and its known isomer (22*R*)-aniduquinolone A (**165**, Figure 10) were purified from the endophytic fungus *Aspergillus versicolor* strain Eich.5.2.2 from the petals of flowers of *Eichhornia crassipes*. The epimers **164/165** together exhibited significant antibacterial activity against *S. aureus*, with a MIC of 0.4 μg/mL [86].

A new diaryl ether derivative aspergillether B (**166**, Figure 10) was separated from *Aspergillus versicolor* residing inside the roots of *Pulicaria crispa*. Compound **166** exhibited significant antibacterial capacity towards *S. aureus, Bacillus cereus*, and *E. coli* with MICs values of 4.3, 3.7, and 3.9 μg/mL, respectively [87].

The known compound 3-O-β-D-glucopyranosyl stigmasta-5(6),24(28)-diene (**167**, Figure 10) was extracted from an endophytic fungus *Aspergillus ochraceus* SX-C7 eus SX-C7 from *Setaginella stauntoniana* and displayed inhibitory activity against *B. subtilis* with a MIC value of 2 μg/mL [88].

A prenylated benzaldehyde derivative, dihydroauroglaucin (**168**, Figure 10), was isolated from *Aspergillus amstelodami* (MK215708) an endophytic fungi of *Ammi majus*, a plant indigenous to Egypt. Compound **168** showed activity against *E. coli*, *Streptococcus mutans* and *S. aureus*, with MICs of 1.95, 1.95 and 3.9 μg/mL, respectively. The highest antibiofilm activity at concentrataion 7.81 μg/mL against *S. aureus* and *E. coli* biofilms, at 15.63 μg/mL concentration against *S. mutans* and moderate activity (MBIC = 31.25 μg/mL) against *P. aeruginosa* biofilm was measured [89].

Two cysteine residue-containing merocytochalasans, cyschalasins A (**169**) and B (**170**, Figure 10) were isolated from *Aspergillus micronesiensis* associated with the root of *Phyllanthus glaucus*. Compounds **169** and **170** displayed anti-MRSA activity with MIC_50_ values of 17.5 and 10.6 μg/mL and MIC_90_ values of 28.4 and 14.7 μg/mL, respectively [90].

Methylsulochrin (**171**, Figure 10) is a diphenyl ether derivative isolated from *A. niger* associated with the stems of *Acanthus montanus*. It inhibits *Enterobacter cloacae*, *Enterobacter aerogenes* and *S. aureus* with MIC values of 7.8, 7.8 and 15.6 μg/mL, respectively [91].

A new furan derivative named 3-(5-oxo-2,5-dihydrofuran-3-yl) propanoic acid (**172**, Figure 10) was purified from *Aspergillus tubingensis*, an endophyte from the stems of *Decaisnea insignis*. Compound **172** inhibited *Streptococcus lactis* with MIC value of 32 μg/mL [92].

A new compound, methyl 2-(4-hydroxybenzyl)-1,7-dihydroxy-6-(3-methylbut-2-enyl)-1*H*-indene-1-carboxylate (**173**, Figure 10) was extracted from *Aspergillus flavipes* Y-62, associated with the plant *Suaeda glauca*. Compound **173** showed poor activity against MRSA, with an MIC value of 128 μg/mL, and against *K. pneumoniae* and *P. aeruginosa* with equal MIC values of 32 μg/mL [93].

The alkaloids 4-amino-1-(1,3-dihydroxy-1-(4-nitrophenyl)propan-2-yl)-1*H*-1,2,3-triazole-5(4H)one (**174**) and 3,6-dibenzyl-3,6-dimethylpiperazine-2,5-dione (**175**, Figure 10) were obtained from *Aspergillus* sp. isolate of *Zingiber cassumunar* rhizome. Compounds **174** and **175** exhibited inhibitory activity against *X. oryzae* and *E. coli*, with a 16–30 mm zone of inhibition [5].

*Aspergillus fumigatus,* an endophyte associated with *Edgeworthia chrysantha,* was the source of pseurotin A (**176**) and spirotryprostatin A (**177**, Figure 10). Compounds **176**, **177** displayed good antibacterial activity against *S. aureus* (MIC 0.39 µg/mL each). Compound **177** also showed potent antibacterial activity against *E. coli* (MIC of 0.39 µg/mL) [94].

**Figure 11:**
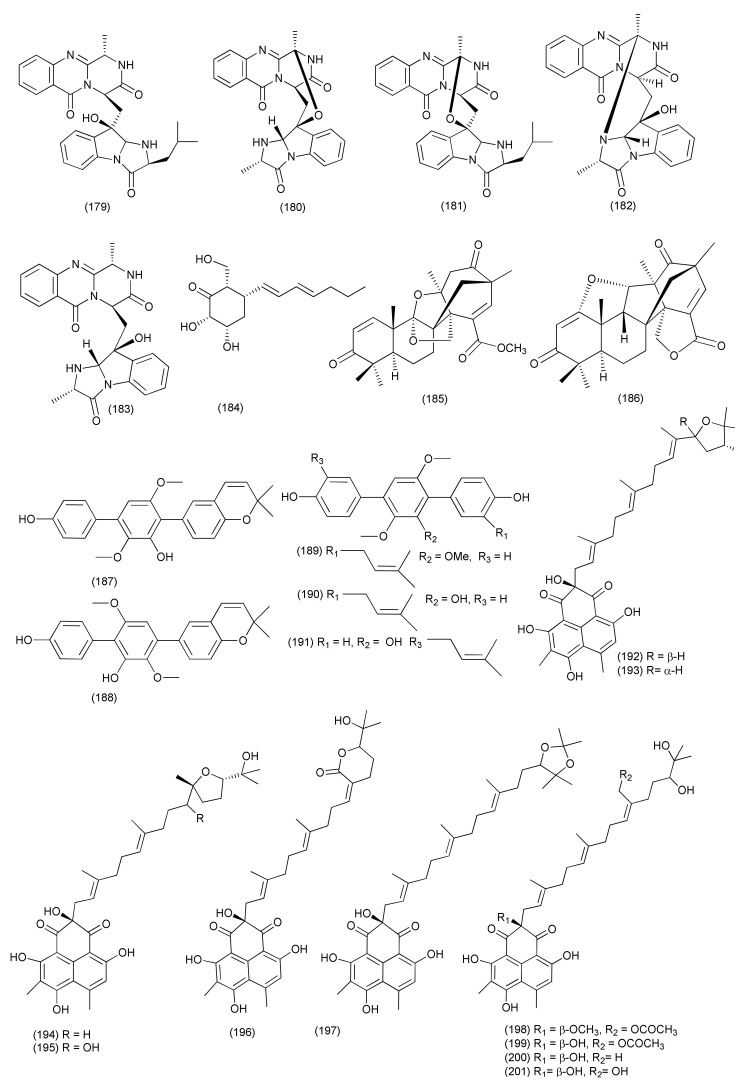
Structures of metabolites **179**–**201** isolated from Anamorphic Ascomycetes.

Six compounds, fumiquinazoline J (**178**, Figure 10), fumiquinazoline I (**179**), fumiquinazoline C (**180**), fumiquinazoline H (**181**), fumiquinazoline D (**182**), and fumiquinazoline B (**183**, Figure 11) were extracted from *Aspergillus* sp., residing inside the plant *Astragalus membranaceus*. Compounds **178**, **180**–**182** displayed potent activity against *B. subtilis*, *E. coli*, *P. aeruginosa* and *S. aureus* (MICs in the range of 0.5–8 μg/mL). Compounds **179**, **183** displayed moderate activity against *B. subtilis*, *E. coli*, *P. aeruginosa* and *S. aureus* with MICs of 4–16 μg/mL [95].

An antibacterial polyketide named (-) palitantin (**184**, Figure 11) was isolated from *Aspergillus fumigatiaffnis*, an endophyte of the medicinal plant *Tribulus terrestris*, which displayed antibacterial activity against *E. faecalis* UW 2689 and *S. pneumoniae* with MIC values of 64 μg/mL each [96].

A novel terpene-polyketide hybrid, i.e., a meroterpenoid, aspermerodione (**185**), and a new heptacyclic analog and iconin C (**186**, Figure 11) were purified from *Aspergillus* sp. TJ23 residing inside the plant *Hypericum perforatum*. Compound **185** showed antibacterial activity against MRSA (MIC of 32 μg/mL), whereas compound **186** showed poor anti- MRSA activity (>100 μg/mL). Aspemerodione (**186**) worked synergistically with the antibiotics oxacillin and piperacillin against MRSA and was found to be a potential inhibitor of PBP2a [97].

*Aspergillus* sp. YXf3, an endophyte residing inside the leaves of *Ginkgo biloba*, yielded some novel *p*-terphenyls named prenylterphenyllin D (**187**), prenylterphenyllin E (**188**), and 2′-O-methylprenylterphenyllin (**189**), along with the known compounds prenylterphenyllin (**190**) and prenylterphenyllin B (**191**, Figure 11). Compounds **187–191** displayed antibacterial activity against *X. oryzae* pv. *oryzicola* and *E. amylovora* with the same MIC values of 20 μg/mL, while compound **191** exhibited activity against *E. amylovora* with a MIC value of 10 μg/mL [98].

Nine new phenalenone derivatives, aspergillussanone D (**192**), aspergillussanone E (**193**), F (**194**) G (**195**) H (**196**), I (**197**), J (**198**), K (**199**), along with two known analogues, the aspergillussanones L (**200** and **201**, Figure 11) were extracted from *Aspergillus* sp. residing inside the plant *Pinellia ternate*. Compound **200** exhibited good antimicrobial activity against *P. aeruginosa*, *S. aureus*, and *B. subtilis* (MIC_50_ values of 1.87, 2.77, and 4.80 μg/mL). Compound **192** exhibited the antibacterial activity against *P. aeruginosa*, and *S. aureus*, (MIC_50_ of 38.47 and 29.91 μg/mL). Compound **193** was found to be selectively active against *E. coli* (MIC_50_ of 7.83 μg/mL). Compound **194** exhibited antimicrobial activity against *P. aeruginosa*, and *S. aureus*, (MIC_50_ values of 26.56, 3.93 and 16.48 μg/mL). Compound **195** inhibited *P. aeruginosa*, and *S. aureus*, (MIC_50_ values of 24.46 and 34.66 μg/mL). Compound **196** inhibited *P. aeruginosa*, and *E. coli*, (MIC_50_ values of 8.59 and 5.87 μg/mL). Compound **197** selectively inhibited *P. aeruginosa*, (MIC_50_ of 12.0 μg/mL). Compound **198** exhibited activity against *P. aeruginosa*, *E. coli* and *S. aureus* with MIC_50_ values of 28.50, 5.34 and 29.87 μg/mL, respectively. Compound **199** exhibited antibacterial activity against *P. aeruginosa*, and *S. aureus*, (MIC_50_ values of 6.55 and 21.02 μg/mL). Compound **201** inhibited *P. aeruginosa*, and *E. coli*, with MIC_50_ values of 19.07 and 1.88 μg/mL, respectively [99].

**Figure 12:**
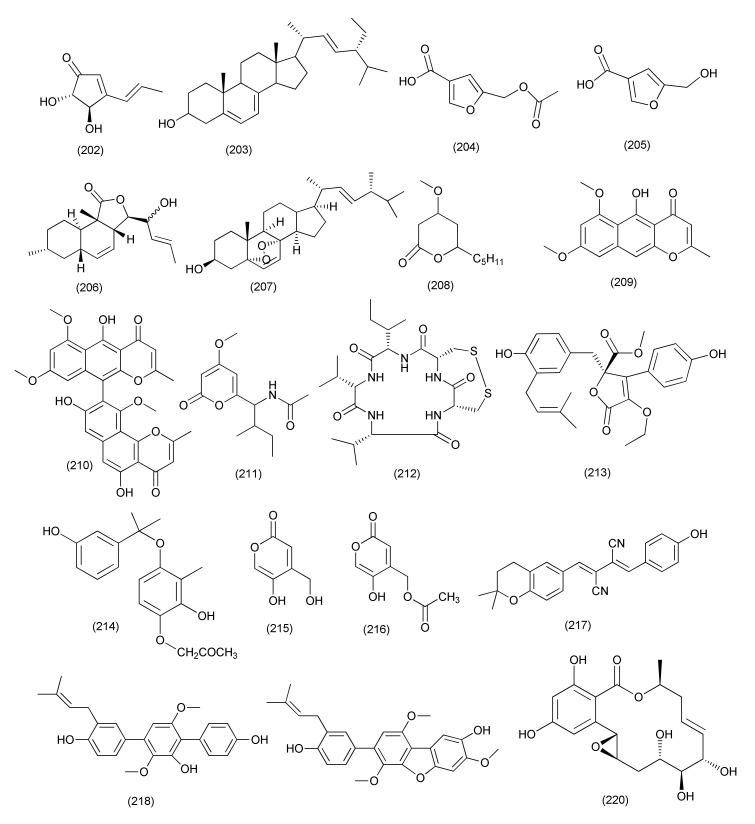
Structures of metabolites **202**–**220** isolated from Anamorphic Ascomycetes.

The compound terrein (**202**, Figure 12), a polyketide, was extracted from *Aspergillus terreus* JAS-2 associated with *Achyranthus aspera*. Terrein (**202**) exhibited antibacterial activity with an IC_50_ value of 20 μg/mL against *E. faecalis*, and more than 20 μg/mL against *Aeromonas hydrophila* and *S. aureus*, as the compound showed only 48% and 38.3% inhibition [100].

A known compound (22*E*,24*R*)-stigmasta-5,7,22-trien-3-β-ol (**203**, Figure 12), was purified from the *Aspergillus terreus* isolate of *Carthamus lanatus*. Compound **203** displayed potent anti-MRSA activity, with IC_50_ values of 2.29 µM compared to ciprofloxacin (IC_50_ 0.21 µM) [101].

A new furan derivative named 5-acetoxymethylfuran-3-carboxylic acid (**204**), along with the furan compound 5-hydroxymethylfuran-3-carboxylic acid (**205**, Figure 12), were obtained from *Aspergillus flavus*, isolated from *Cephalotaxus fortunei*. The compounds **204–205** inhibited *S. aureus* with MIC values of 15.6 and 31.3 μg/mL, respectively [102].

A new compound, allahabadolactone B (**206**), and the known compound ergosterol peroxide (**207**, Figure 12) were purified from *Aspergillus allahabadii* BCC45335 residing inside the roots of *Cinnamomum subavenium*. Compounds **206**–**207** displayed antimicrobial activity against *B. cereus* with IC_50_ values of 12.50 and 3.13 µg/mL, respectively [103].

A new pyrone named 6-isovaleryl-4-methoxy-pyran-2-one (**208**), along with three known pyrone compounds, rubrofusarin B (**209**), asperpyrone A (**210**) and campyrone A (**211**, Figure 12), was purified from *Aspergillus tubingensis* isolated from the roots of *Lycium ruthenicum*. Compound **209** possessed potent activity against *E. coli* with a MIC of 1.95 μg/mL while the compounds **208**, **210**, **211** showed poor activity against *E. coli*, *P. aeruginosa*, *S. aureus* and *Streptococcus lactis* [104].

A new cyclic pentapeptide, malformin E (**212**, Figure 12), was extracted from *Aspergillus tamarii* FR02 associated with *Ficus carica*. Compound **212** displayed potent activity against *B. subtilis*, *S. aureus*, *P. aeruginosa*, and *E. coli* with MIC values of 0.91, 0.45, 1.82, and 0.91 μM, respectively [105].

A new butyrolactone, aspernolide F (**213**), together with a known stigmasterol derivative, (22*E*,24*R*)-stigmasta-5,7,22-trien-3-β-ol (**203**, Figure 12), were purified from *Aspergillus terreus*, an endophyte of *Carthamus lanatus*. Compound **203** displayed a potent anti-MRSA activity, with an IC_50_ value of 0.96μg/mL while compound **213** displayed poor anti-MRSA activity (IC_50_ 6.39μg/mL) [106].

The metabolites 1-(3,8-dihydroxy-4,6,6-trimethyl-6*H*-benzochromen-2-yloxy)propane-2-one (**214**), 5-hydroxy-4-(hydroxymethyl)-2*H*-pyran-2-one (**215**) and 5-hydroxy-2-oxo-2*H*-pyran-4-yl)methyl acetate (**216**, Figure 12) were purified from *Aspergillus* sp. (SbD5) associated with the plant *Andrographis paniculata*. Compounds **214**–**216** displayed poor to average activity against *S. aureus*, *E. coli*, *S. dysenteriae* and *Salmonella typhi* with an inhibition zone diameter ranging from 8.1 to 12.1 mm at a concentration 500 μg/mL [107].

The compounds xanthoascin (**217**), prenylterphenyllin B (**218**) and prenylcandidusin (**219**, Figure 12), were extracted from *Aspergillus* sp. IFB-YXS, associated with the leaves of *Ginkgo biloba*. Compound **217** displayed antibacterial activity against *X. oryzae* pv. *oryzicola*, *E. amylovora*, *P. syringae* pv. *lachrymans* and *C. michiganense* subsp. *sepedonicus* with MICs of 20, 10, 5.0 and 0.31 µg/mL, respectively. Compound **218** exhibited antibiotic activities with MICs of 20 µg/mL each towards *X. oryzae* pv. *oryzicola*, *E. amylovora*, *P. syringae* pv. *lachrymans*, respectively. Compound **219** was found to be effective against *X. oryzae* pv. *oryzae* and *X. oryzae* pv. *oryzicola* (MIC of 10 and 20 µg/mL). It was observed that compound **217** can change the permeability and cause nucleic acid leakage of the cytomembrane of the phytopathogen [108].

#### 2.2.2. *Penicillium*

**Figure 13:**
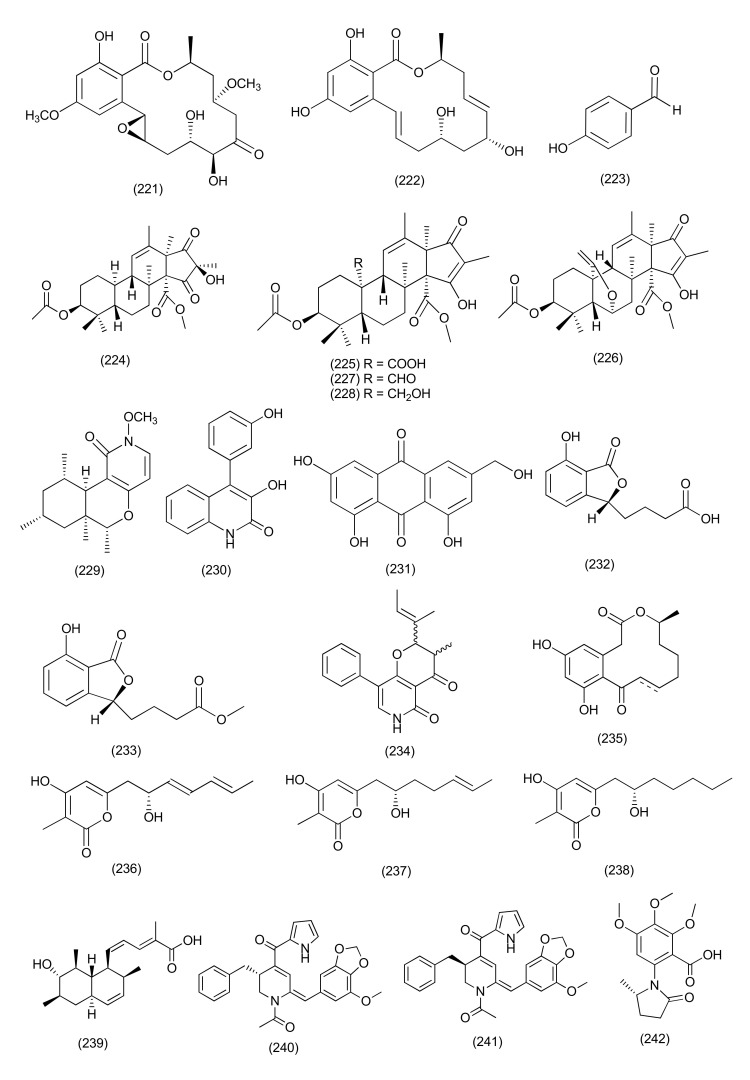
Structures of metabolites **221**–**242** isolated from Anamorphic Ascomycetes.

New β-resorcylic acid lactones, including 4-O-desmethyl-aigialomycin B (**220**, Figure 12), and penochrochlactones C (**221**), and D (**222**, Figure 13), were purified from *Penicillium ochrochloron* SWUKD4.1850 from the medicinal plant *Kadsura angustifolia*. Compounds **220**–**222** exhibited moderate activities against *S. aureus*, *B. subtilis*, *E. coli*, and *P. aeruginosa* with MIC values between 9.7 and 32.0 μg/mL [109].

The compound *p*-hydroxybenzaldehyde (**223**, Figure 13), was isolated from *Penicillium brefeldianum*, an endophyte residing inside the root bark of *Syzygium zeylanicum*. Compound **223** was found to be active against *S. typhi*, *E. coli*, and *B. subtilis* with MIC values of 64 g/mL. *p*-Hydroxybenzaldehyde was also reported from *Syzygium zeylanicum* [110].

An endophytic fungus, *Penicillium vulpinum* GDGJ-91, from the roots of *Sophorae tonkinensis*, yielded the new compound 10-demethylated andrastone A (**224**), and four known analogs, 15-deacetylcitreohybridone E (**225**), citreohybridonol (**226**) and andrastins A (**227**) and B (**228**, Figure 13). Compounds **224** and **227** displayed good activity against *Bacillus megaterium* (MIC value of 6.25 μg/mL), and compounds **225**, **226**, **228** showed average activity against *Bacillus megaterium* (MIC of 25, 12.5 and 25 μg/mL). Compound **226** showed potent antibacterial activity against *B. paratyphosus* B at 6.25 μg/mL, while the other compounds showed average activities against *B. paratyphosus* B at 12.5 or 25 μg/mL and compound **226** also exhibited moderate activities against *E. coli* and *S. aureus* with MIC values of 25 μg/mL [111].

A novel N-methoxy-1-pyridone alkaloid, chromenopyridin A (**229**), and the already reported compound viridicatol (**230**, Figure 13) were purified from *Penicillium nothofagi* P-6, residing inside the bark of *Abies beshanzuensis*. Compounds **229** and **230** exhibited antibacterial activity against *S. aureus,* with MIC values of 62.5 and 15.6 μg/mL, respectively [112].

ω-Hydroxyemodin (**231**, Figure 13) a polyhydroxy anthraquinone, was extracted from *Penicillium restrictum* (strain G85) from *Silybum marianum*. Compound **231** showed inhibition against MRSA as a quorum sensing inhibitor in both in vitro and in vivo systems [113].

Two new phthalide derivatives, (−)-3-carboxypropyl-7-hydroxyphthalide (**232**) and (−)-3-carboxypropyl-7-hydroxyphthalide methyl ester (**233**, Figure 13), were isolated from *Penicillium vulpinum* residing inside the plant *S. tonkinensis*. Compound **232** exhibited a medium inhibition against *Shigella dysenteriae*, *Enterobacter areogenes*, *B. subtilis*, *B. megaterium*, and *Micrococcus lysodeikticus* with MIC value between 12.5–50 μg/mL. Compound **233** showed average activity against *E. areogenes* with MIC value of 12.5 μg/mL, and showed poor activity against *B. subtilis, B. megaterium* and *M. lysodeikticus* with MIC values of 100 μg/mL [114].

Citridone E (**234**), a new phenylpyridone derivative, and the previously reported compound (–)-dehydrocurvularin (**235**, Figure 13) were purified from *Penicillium sumatrense* GZWMJZ-313 associated with the plant *Garcinia multiflora*. Compounds **234** and **235** showed antibacterial activity against *S. aureus*, *P. aeruginosa*, *Clostridium perfringens*, and *E. coli* (with MICs ranging from 32 to 64 μg/mL) [115].

Three new 3,4,6-trisubstituted α-pyrone derivatives, namely 6-(2′*R*-hydroxy-3′*E*,5′*E*-diene-1′-heptyl)-4-hydroxy-3-methyl-2*H*-pyran-2-one (**236**), 6-(2′*S*-hydroxy-5′*E*-ene-1′-heptyl)-4-hydroxy-3-methyl-2*H*-pyran2-one (**237**), and 6-(2′*S*-hydroxy-1′-heptyl)-4-hydroxy-3-methyl-2*H*-pyran-2-one (**238**), along with the previously reported compound trichodermic acid (**239**, Figure 13), were purified from *Penicillium ochrochloron* associated with *Taxus media*. Compounds **236**–**239** displayed antimicrobial activity with MIC values ranging from 25 to 50 μg/mL against *B. subtilis*, *B. megaterium*, *E. coli*, *Enterobacter aerogenes*, *Micrococcus luteus*, *Proteusbacillm vulgaris*, *P. aeruginosa*, *S. aureus*, *Salmonella enterica*, and *Salmonella typhi* [116].

**Figure 14:**
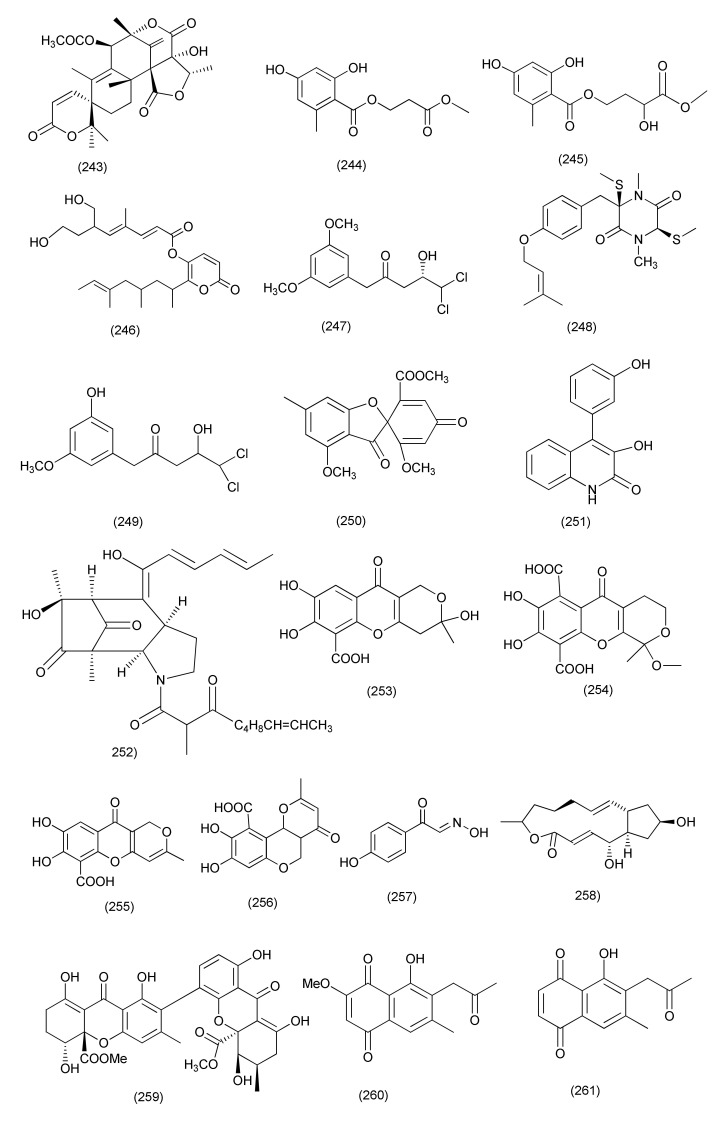
Structures of metabolites **243**–**261** isolated from Anamorphic Ascomycetes.

Three new compounds, brasiliamide J-a (**240**), brasiliamide J-b (**241**) and peniciolidone (**242**, Figure 13), as well as the known compound austin (**243**, Figure 14), were isolated from *Penicillium janthinellum* SYPF 7899 associated with the plant *Panax notoginseng*. Compound **240** exhibited potent activity against *B. subtilis* and *S. aureus* (MICs of 15 and 18 μg/mL). Compounds **241** and **243** showed average inhibitory activities against *B. subtilis* (MIC 35 μg/mL and 50 μg/mL, respectively) and *S. aureus* (MIC 39 μg/mL and 60 μg/mL, respectively). In addition, compound **240** also affected the length of *B. subtillius*. Similarly, coccoid cells of *S. aureus* also swelled 2-fold after treatment with compound **240**. Compounds **240, 241, 242** showed high binding energies, strong H-bond interactions and hydrophobic interactions with filamentous temperature-sensitive protein Z (FtsZ) [117].

The new compounds penicimenolidyu A (**244**), and penicimenolidyu B (**245**) and the known compound rasfonin (**246**, Figure 14) were purified from *Penicillium cataractarum* SYPF 7131 obtained from the plant *Ginkgo biloba*. Compound **246** exhibited good antibacterial activity against *S. aureus*, with a MIC value of 10 μg/mL. Compounds **245** and **246** showed moderate inhibitory activity against *S. aureus* (MIC 65 μg/mL and 59 μg/mL). The docking results revealed that compounds **244–246** possess high binding energies, strong H-bond interactions and hydrophobic interactions with FtsZ from *S. aureus*, validating the observed antimicrobial activity [118].

A rare dichloroaromatic polyketide, 3′-methoxycitreovirone (**247**) along with known metabolites *cis*-bis-(methylthio)-silvatin (**248**), citreovirone (**249**), trypacidin A (**250**, Figure 14) and helvolic acid (**100**), were obtained from endophytic *Penicillium* sp. of *Pinellia ternate*. Compound **100** displayed potent antibacterial activity against *S. aureus* and *P. aeruginosa* (MIC = 5.8 and 4.6 μg/mL) as well as mild activity against *B. subtilis* and *E. coli* (MIC = 42.2 and 75.0 μg/mL). Compounds **247** and **249** were found to have moderate antibacterial activity against *E. coli* and *S. aureus* (MIC = 62.6 and 76.6 μg/mL). Compounds **248** and **250** exhibited poor antibacterial activity against *S. aureus* with MIC values of 43.4 and 76.0 μg/mL and **250** also displayed effect against *B. subtilis* (MIC = 54.1 μg/mL) [119].

A known quinolinone alkaloids viridicatol (**251**, Figure 14) was obtained from *Penicillium* sp. R22 was associated with *Nerium indicum* and displayed potent antibacterial activity against *S. aureus* with MIC value of 15.6 μg/mL [120]. The novel compound penicitroamide (**252**, Figure 14), was purified from *Penicillium* sp. (NO. 24) isolated from the leaves of *Tapiscia sinensis*. Compound **252** displayed potent antibacterial activity against plant pathogens, *Erwinia carotovora* sub sp. *carotovora* (Jones) Bersey, et al. with MIC_50_ at 45 μg/mL [121].

Penialidins A-C (**253**–**255**), citromycetin (**256**), *p*-hydroxyphenylglyoxalaldoxime (**257**) and brefelfin A (**258**, Figure 14) were purified from the *Penicillium* sp. CAM64 a fungus associated with the plant *Garcinia nobilis*. Compounds **253**–**258**, exhibited antibacterial activity against *Vibrio cholerae* SG24 (1), *V. cholerae* CO6, *V. cholerae* NB2, *V. cholerae* PC2, *S. flexneri* SDINT (MIC = 0.50–128 μg/mL). Compound **255** exhibited potent activity against *V. cholerae* SG24 (1), *V. cholerae* CO6, *V. cholerae* NB2, *V. cholerae* PC2, *S. flexneri* SDINT, with MIC values of 0.50, 16, 8, 0.50 and 8 μg/mL, respectively following in decreasing order of activity by compound **254** (MIC = 4–32 μg/mL), compound **257** (MIC = 8–32 μg/mL), compound **257** (MIC = 32–64 μg/mL) and compounds **256** and **258** (MIC = 64–128 μg/mL) [122].

Purpureone (**259**, Figure 14) was extracted from *Purpureocillium lilacinum*, residing inside the roots of *Rauvolfia macrophylla*. Compound **259** displayed antibacterial activity with the zone of inhibition of 10.6, 12.3, 13.0, 8.7, 12.3, and 10.0 mm against *B. cereus*, *L. monocytogenes*, *E. coli*, *K. pneumoniae*, *P. stuartii*, and *P. aeruginosa* (6 mm filter paper disks impregnated with 10 μL of compound) [123].

#### 2.2.3. *Fusarium*

**Figure 15:**
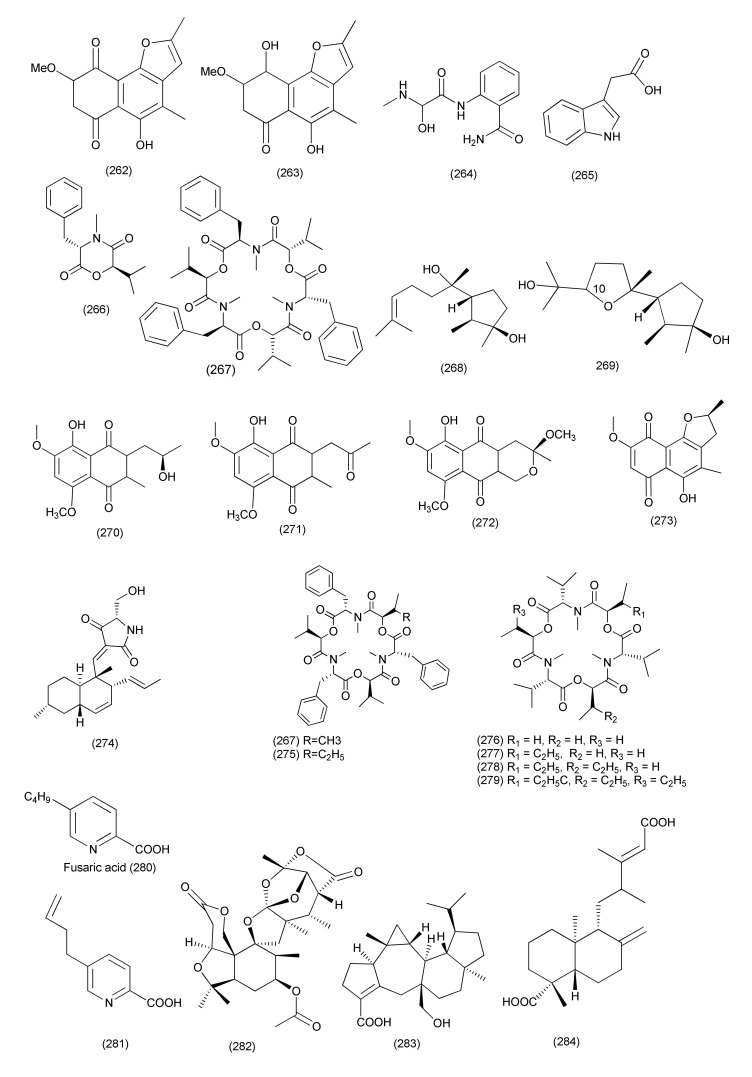
Structures of metabolites **262**–**284** isolated from Anamorphic Ascomycetes.

Secondary metabolites identified as 2-methoxy-6-methyl-7-acetonyl-8-hydroxy-1,4-maphthalenedione (**260**) 5,8-dihydroxy-7-acetonyl-1,4-naphthalenedione (**261**, Figure 14), anhydrojavanicin (**262**), and fusarnaphthoquinone B (**263**, Figure 15), were purified from *Neocosmospora* sp. MFLUCC 17-0253 associated with *Rhizophora apiculata*. All three compounds showed potent antibacterial against *Acidovorax citrulli* (responsible for bacterial fruit blotch (BFB) a bacterial disease of *Cucurbitaceae* crops) with MIC values of 0.0075 mg/mL (mixture of **260**, **261**), 0.004 mg/mL (**262**), 0.025 mg/mL (**263**). Compounds **260**–**263** significantly inhibited biofilm development of *Acidovorax citrulli*, thus demonstrating that these metabolites can be used for biological control of bacterial fruit blotch of watermelon and melon [124].

A new aminobenzamide derivative, namely fusaribenzamide A (**264**, Figure 15), was purified from *Fusarium* sp. of *Mentha longifolia*. Compound **264** displayed antibacterial activity against *S. aureus* and *E. coli* with MIC values of 62.8 and 56.4 μg/disc, respectively [125].

Two alkaloids, indol-3-acetic acid (**265**), bassiatin (**266**), a depsipeptide, beauvericin (**267**), two sesquiterpenoids, cyclonerodiol (**268**), epicyclonerodiol oxide (**269**), four 1,4-naphthoquinones, 5-O-methylsolaniol (**270**), 5-O-methyljavanicin (**271**), fusarubin methyl ether (**272**), and anhydrojavanicin (**273**, Figure 15) and a sesterterpene, fusaproliferin (**106**), were separated from the green Chinese onion-derived fungus *F. proliferatum* AF-04. Compounds **270**–**273** displayed good antibacterial activity against *B. megaterium* with MICs of 25 μg/mL each; compounds **265**, **267**, **269** displayed moderate activity with MICs of 50 μg/mL each and compound **268**, displayed activity with an MIC of 12.50 μg/mL. Compounds **266**, **270**–**272** displayed good antibacterial activity against *B. subtilis*, with MICs of 50 μg/mL each. Compounds **269** and **272** were found to be active against *E. coli* with MIC values of 50 μg/mL each and compounds **270**, **271**, **273** with MIC values of 25 μg/mL, respectively. Compounds **269**–**272** displayed antibacterial activity against *Clostridium perfringens* with MIC values of 50, 50, 12.5 and 50 μg/mL, respectively. Compounds **267**, **106**, **270**–**273** displayed anti-MRSA activity with MIC values of 50, 50, 12.5, 12.5, 12.5, and 25μg/mL, respectively. Compounds **270**–**273** displayed antibacterial activity against RN4220 (MICs of 50 μg/mL each). Compounds **272**, **273** showed inhibition against NewmanWT (MICs of 50 μg/mL each). Compound **266** displayed antibacterial activity against NewmanWT with a MIC value of 50 μg/mL each. [126].

*Fusarium* sp. TP-G1 an endophyte of *Dendrobium officinable*, was the source of the compounds trichosetin (**274**), beauvericin A (**275**), enniatin B (**276**), enniatin H (**277**), enniatin I (**278**), enniatin MK1688 (**279**), fusaric acid (**280**) and dehydrofusaric acid (**281**, Figure 15) and beauvericin (**267**). Compounds **267**, **274**, **275**, **277**–**279** displayed antibacterial activity against *S. aureus* and MRSA with IC_50_ values in the range of 2–32 μg/mL. Compounds **280**, **281** displayed antimicrobial activity against *Acinetobacter baumannii* with a MIC value of 64 μg/mL and 128 μg/mL, respectively. Compound **276** inhibited *S. aureus* and MRSA with IC_50_ value of 128 μg/mL each [127].

A new spiromeroterpenoid, namely fusariumin A (**282**), together with the previously reported terpenoids asperterpenoid A (**283**) and agathic acid (**284**, Figure 15), were purified from *Fusarium* sp. YD-2 associated with the plant *Santalum album*. Compound **282** showed antibacterial activity against pathogenic *S. aureus* and *P. aeruginosa* (MIC of 6.3 μg/mL), and compound **283** showed average activity against pathogenic *Salmonella enteritidis* and *Micrococcus luteus* (MICs of 25.2 and 6.3 μg/mL). Compound **284** showed moderate activities against *B. cereus* and *M. luteus*, with MIC values of and 12.5 and 25.4 μg/mL, respectively [128].

**Figure 16:**
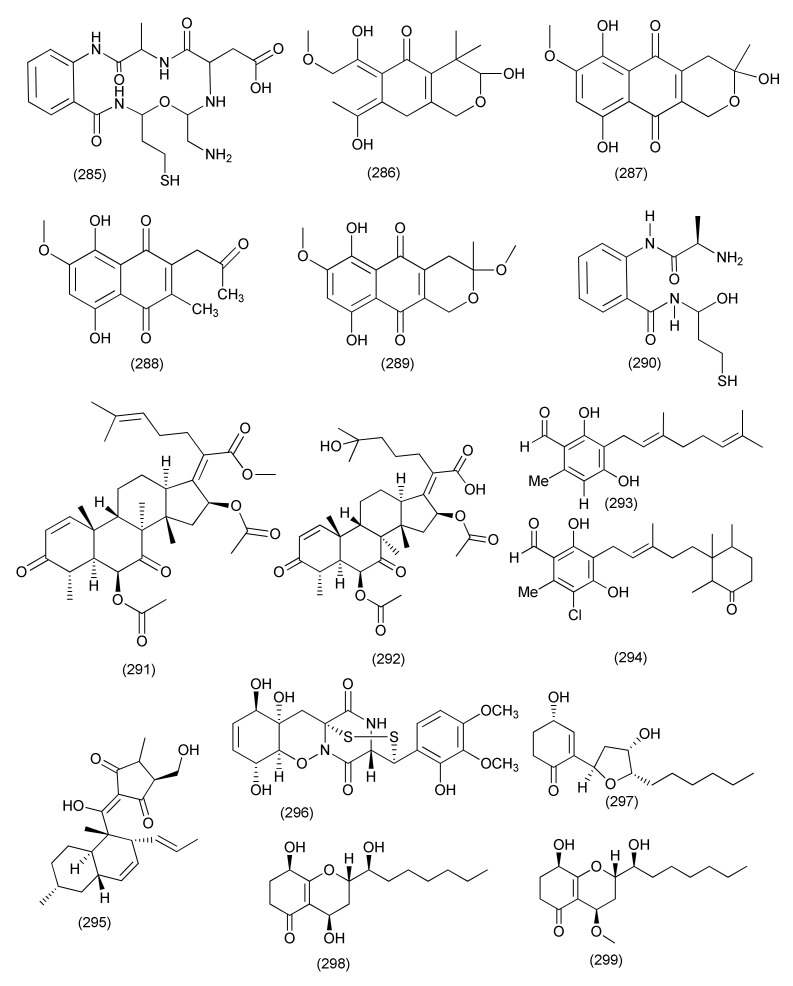
Structures of metabolites **285**–**299** isolated from Anamorphic Ascomycetes.

A new aminobenzamide derivative, namly fusarithioamide B (**285**, Figure 16), was separated from *Fusarium chlamydosporium* an endophyte of *Anvillea garcinii* and exhibited antibacterial activity against *E. coli*, *B. cereus*, and *S. aureus* (MIC values of 3.7, 2.5 and 3.1 µg/mL) [129].

The compounds 3,6,9-trihydroxy-7-methoxy4,4-dimethyl-3,4-dihydro-1*H*-benzo[g] isochromene-5,10-dione (**286**), fusarubin (**287**), 3-O-methylfusarubin (**288**) and javanicin (**289**, Figure 16) were extracted from *Fusarium solani* A2 residing inside the plant *Glycyrrhiza glabra*. Compounds **286**–**289** showed inhibition of *B. subtilis*, *B. cereus*, *E. coli*, *S. aureus*, *K. pneumonia*, *S. pyogenes*, and *Micrococcus luteus* (MICs in the range of < 1 to 256 μg/mL). Fusarubin (**287**) showed good activity against *M. tuberculosis* strain H37Rv with a MIC value of 8 μg/mL, whereas compounds **286**, **288**, **289** exhibited moderate activity with MIC values of 256, 64, 32 μg/mL, respectively [130].

A new benzamide derivative, fusarithioamide A (**290**, Figure 16) was characterized from *Fusarium chlamydosporium*, an endophyte of *Anvillea garcinii*. Compound **290** had antibacterial potential towards *B. cereus*, *S. aureus,* and *E. coli* with MIC values of 3.1, 4.4, and 6.9 μg/mL, respectively [131].

The polyketide javanicin (**289**, Figure 16) was purified from *Fusarium* sp. associated with *Rhoeo spathacea*, and displayed activity against *M. tuberculosis* with a MIC value of 25 μg/mL and *M. phlei* with a MIC value of 50 μg/mL [132].

Helvolic acid methyl ester (**291**, Figure 16), a new helvolic acid derivative, together with previously reported hydrohelvolic acid (**292**, Figure 16), and helvolic acid (**100**) were isolated from a *Fusarium* sp. residing inside the plant *Ficus carica*. Compound **291** was found to be active against *B. subtilis*, *S. aureus*, *E. coli* and *P. aeruginosa* (MIC between 3.13 to 12.5, μg/mL). Compound **100** displayed activity against *B. subtilis*, *S. aureus*, *E. coli* and *P. aeruginosa* (MICs between 3.13 to 6.25 μg/mL). Compound **292** displayed activity against *B. subtilis*, *S. aureus*, *E. coli* and *P. aeruginosa* with MIC values between 3.13 to 12.5 μg/mL [133].

The compounds colletorin B (**293**) and 4,5-dihydroascochlorin (**294**, Figure 16) were purified from an endophytic *Fusarium* sp. fungus. Compounds **293** and **294** exhibited potent antibacterial activity towards *B. megaterium*, with 5 and 10 mm zones of inhibition at a concentration of 10 μg/mL [134].

The tetramic acid derivative equisetin (**295**, Figure 16) was isolated from a *Fusarium* sp. associated with *Opuntia dillenii*, and displayed antibacterial activity against *B. subtilis* with a MIC value of 8 and MICs of 16 μg/mL against *S. aureus* and MRSA [135].

#### 2.2.4. *Trichoderma*

Pretrichodermamide A (**296**, Figure 16), a known compound, was isolated from *Trichoderma harzianum*, an endophyte of *Zingiber officinale* and displayed antimycobacterial activity towards *M. tuberculosis* with a MIC value of 25 μg/mL (50 μM) [136].

**Figure 17:**
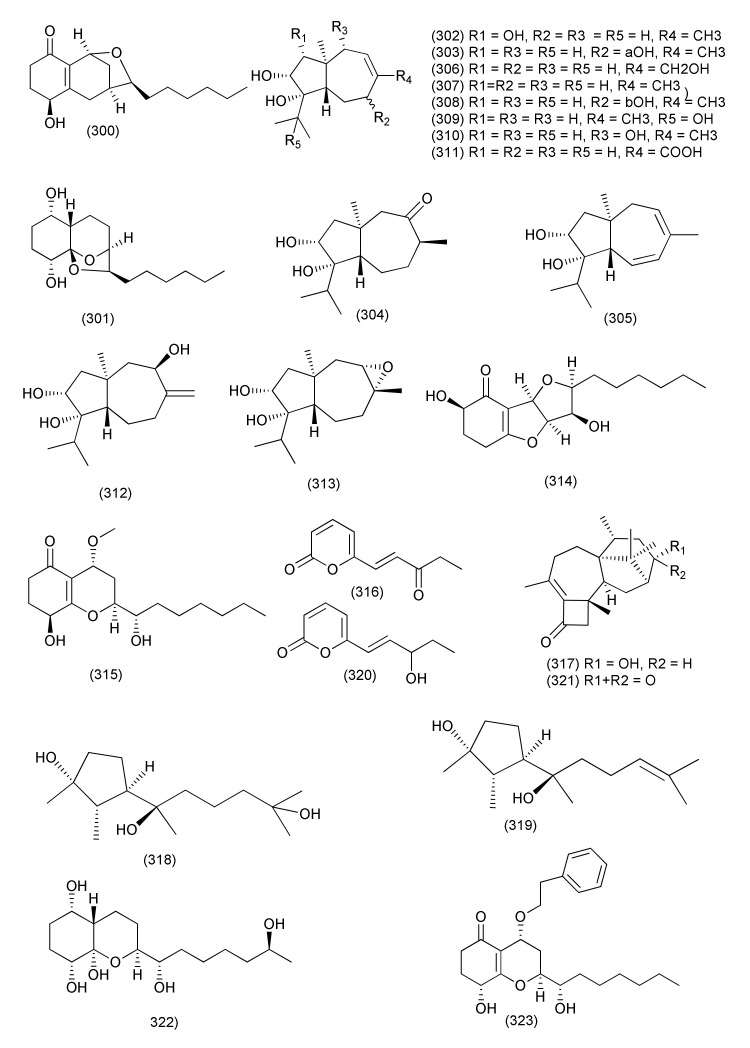
Structures of metabolites **300**–**323** isolated from Anamorphic Ascomycetes.

A new compound named koninginin W (**297**) and four known polyketides, namely koninginin D (**298**), 7-O-methylkoninginin D (**299**, Figure 16), koninginin T (**300**) and koninginin A (**301**, Figure 17) were isolated from the endophytic fungus *Trichoderma koningiopsis* YIM PH30002 of *Panax notoginseng*. Compounds **297**, **298**, **301**, showed the weak activity against *B. subtilis* with MICs of 128 μg/mL. Compounds **297** and **299**, showed weak activity against *S. typhimurium*, with MIC values of 64 and 128 μg/mL; Compounds **297** and **300**, showed the weak activity against *E. coli* with MICs of 128 μg/mL. [137].

Five new carotane sesquiterpenes, trichocarotins I–M (**302**–**306**), which have diverse substitution patterns, and seven known related analogues including CAF-603 (**307**), 7β-hydroxy CAF-603 (**308**), trichocarotins E–H (**309**–**312**), and trichocarane A (**313**, Figure 17) were purified from *Trichoderma virens* QA-8, an endophytic fungus associated with the inner root tissue of *Artemisia argyi*. Compounds **302**–**313** displayed antibacterial activity against *E. coli* EMBLC-1, with MIC values ranging from 0.5 to 32 µg/mL, while 7β-hydroxy CAF-603 (**308**) displayed potent activity against *Micrococcus luteus* QDIO-3 (MIC = 0.5 µg/mL) [138].

Three new polyketides, trichodermaketone E (**314**), 4-*epi*-7-O-methylkoninginin D (**315**), and trichopyranone A (**316**), two new terpenoids, 3-hydroxyharziandione (**317**) and 10,11-dihydro-11-hydroxycyclonerodiol (**318**), together with three related known congeners, cyclonerodiol (**319**), 6-(3-hydroxypent-1-en-1-yl)-2*H*-pyran-2-one (**320**), and harziandione (**321**, Figure 17) were isolated from the endophytic fungus *Trichoderma koningiopsis* QA-3 associated with the plant *Artemisia argyi*. Compounds **314**, **316**–**318**, **321** displayed potent activities against *E. coli*, with MIC values ranging from 0.5 to 64 μg/mL, while compounds **316**–**321** showed inhibitory activities against *M. luteus* with MIC values ranging from 1 to 16 μg/mL, compounds **314**, **315**, **317**–**321**, showed inhibitory activities against *P. aeruginosa* with MIC values ranging from 4 to 16 μg/mL, and compounds **314**, **318**–**321** showed activities against *V. parahaemolyticus* with MIC values ranging from 4 to 16 μg/mL. Among the compounds tested, compound **317** showed the strongest activity against *E. coli*, with a MIC value of 0.5 µg/mL and compound **320** showed the strongest activity against *M. luteus*, with a MIC value of 1 µg/mL, comparable to that of the positive control chloramphenicol [139].

**Figure 18:**
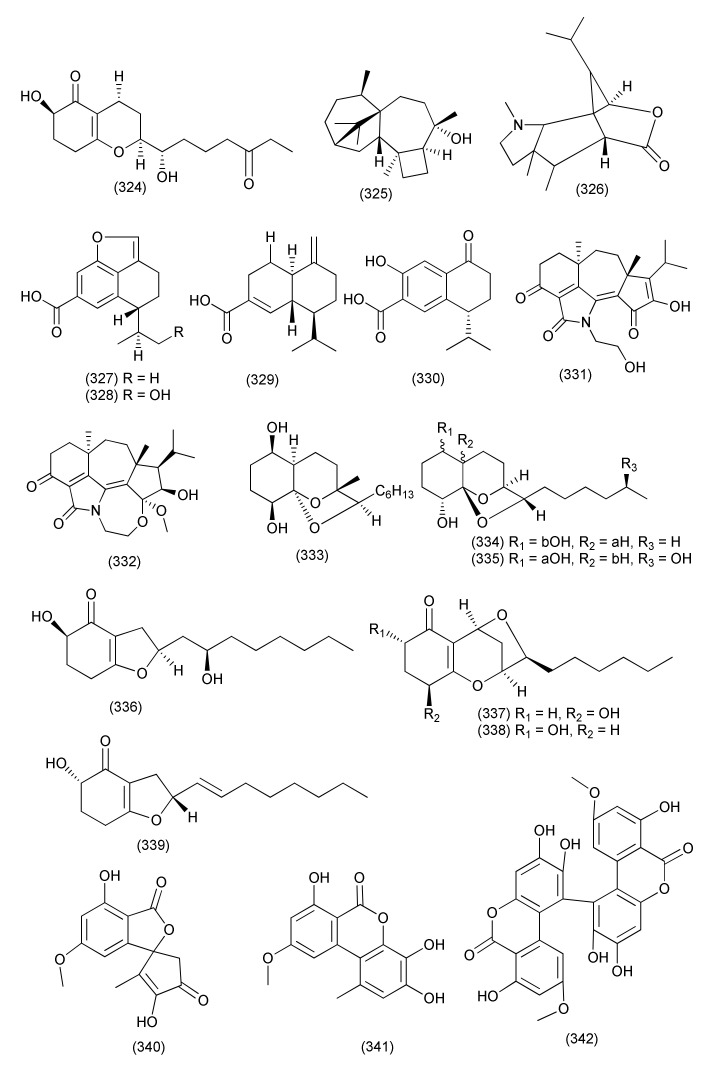
Structures of metabolites **324**–**342** isolated from Anamorphic Ascomycetes.

New highly oxygenated polyketides, 15-hydroxy-1,4,5,6-tetra-*epi*-koninginin G (**322**), koninginin U (**323**, Figure 17) and 14-ketokoninginin B (**324**, Figure 18), were isolated from *Trichoderma koningiopsis* QA-3, isolated from *Artemisia argyi*. Compound **322** displayed good activity against the aquatic pathogen *Vibrio alginolyticus,* with a MIC value of 1 μg/mL. Compounds **323**, **324** exhibited activity against aquatic bacteria *Vibrio harveyi* and *Edwardsiella tarda* with MICs of 4 and 2 µg/mL, respectively [140].

A new harziane diterpenoid with a 4/7/5/6 tetracyclic scaffold, harzianol I (**325**, Figure 18) was isolated from *Trichoderma atroviride* B7, an endophyte associated with the plant *Colquhounia coccinea* var. *mollis*. Compound **325** exhibited potent inhibitory activity against *S. aureus*, *B. subtilis*, and *M. luteus*, with EC_50_ values of 7.7, 7.7, and 9.9 μg/mL, respectively [141].

The compound dendrobine (**326**, Figure 18) was purified from *Trichoderma longibrachiatum* MD33, an endophyte of *Dendrobium nobile*. Compound **326** inhibited *Bacillus mycoides*, *B. subtilis*, and *Staphylococcus* spp., with zones of inhibition of 9, 12 and 8 mm, respectively [142].

Trichocadinins B-D and G (**327**–**330**, Figure 18), new cadinane-type sesquiterpene derivatives, were isolated from *Trichoderma virens* QA-8 residing inside the plant *Artemisia argyi*. Compounds **327**–**330** displayed antibacterial activity against *E. coli*, *Aeromonas hydrophilia* QDIO-1, *Edwardsiella tarda*, *E. ictarda*, *Micrococcus luteus*, *P. aeruginosa*, *Vibrio alginolyticus*, *V. anguillarum*, *V. harveyi*, *V. parahemolyticus*, and *V. vulnificus* (MICs in the range of 8–64 μg/mL). Compound **330** inhibited *Ed. tarda* and *V. anguillarum* with MIC values of 1 and 2 μg/mL, respectively [143].

New diterpenes koninginols A (**331**) and B (**332**, Figure 18) were isolated from *Trichoderma koningiopsis* A729, an endophyte of *Morinda officinalis*. Compounds **331**–**332** exhibited potent inhibition against *B. subtilis*, with MIC values of 10 and 2 μg/mL, respectively [144].

*Trichoderma koningiopsis* QA-3, isolated from the plant *Artemisia argyi*, produced five new polyketides: ent-koninginin A (**333**), 1,6-di-*epi-*koninginin A (**334**), 15-hydroxykoninginin A (**335**), 10-deacetylkoningiopisin D (**336**) and koninginin T (**337**) and two known analogs, koninginin L (**338**), trichoketide A (**339**, Figure 18). Compounds **333** and **339** inhibited the aquatic bacteria *E. tarda*, *V. anguillarum*, and *V. parahemolyticus*, and the human pathogen *E. coli* (MICs ranging from 8 to 64 μg/mL). Compound **333** also showed activity against the aquatic bacteria *M. luteus* and *P. aeruginosa* and agropathogens. Compounds **333**–**339** were found to be active against *E. coli* (each with MIC values of 64 μg/mL) and *E. tarda*, *V. alginolyticus*, and *V. anguillarum* (MICs ranging from 8 to 64 μg/mL) while compounds **333** and **339** also showed antimicrobial activity against *M luteus*, *V. parahemolyticus*, and *V. vulnificus* (MIC values ranging from 4 to 64 μg/mL). Compound **333** was also found active against *V. vulnificus* with a MIC of 4 μg/mL [145].

#### 2.2.5. *Alternaria*

A novel polyketide derivative, isotalaroflavone (**340**), along with the known compounds 4-hydroxyalternariol-9-methyl ether (**341**) and verrulactone A (**342**, Figure 18) were obtained from *Alternaria alternata* ZHJG5 that was isolated from the leaves of *Cercis chinensis* collected from Nanjing Botanical Garden (Nanjing, China). Compounds **340**–**342** were found to be active against *Xanthomonas oryzae* pv. *oryzae* (Xoo), *Xanthomonas oryzae* pv. *oryzicola* (Xoc) and *Ralstonia solanacearum* (Rs) with MICs ranging from 0.5 to 64 μg/mL. In addition, compound **340** showed a potent protective effect against rice bacterial leaf blight caused by Xoo with a protective efficacy of 75.1% at a concentration of 200 μg/mL [146].

**Figure 19:**
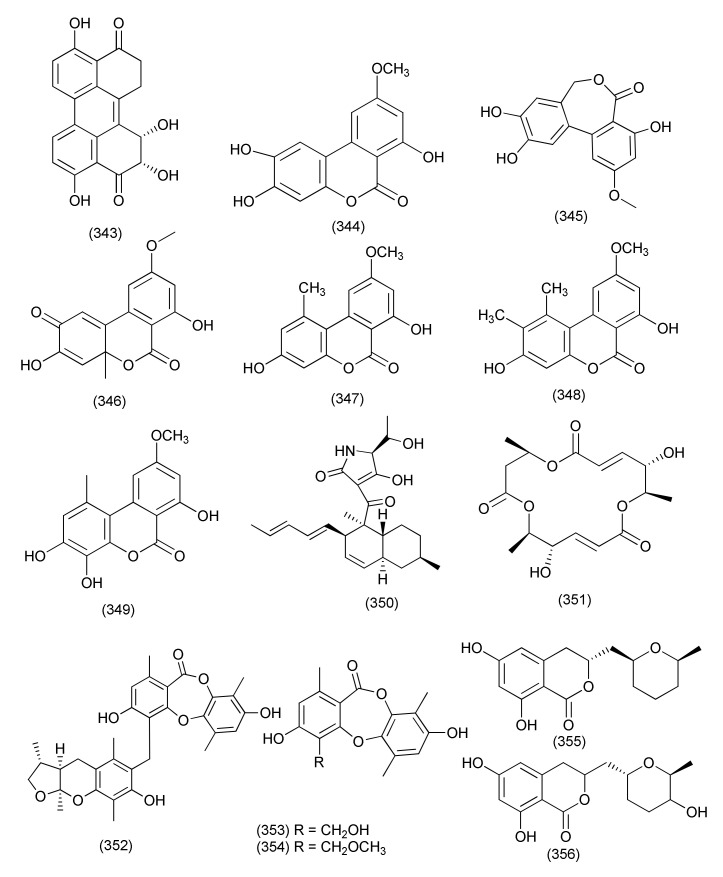
Structures of metabolites **343**–**356** isolated from Anamorphic Ascomycetes.

A new biphenyl compound altertoxin VII (**343**), and the related compounds altenuisol (**344**, Figure 19), alternariol (**44**), were purified from *Alternaria* sp. PfuH1 is associated with *Pogostemon cablin*. Compounds **44**, **343**, **344** showed activity against *S. agalactiae* with MIC values of 9.3, 17.3, and 85.3, μg/mL, respectively, and compound **343** also showed poor activity against *E. coli* with MIC value of 128 μg/mL [147].

Known metabolites altenuisol (**344**), alterlactone (**345**), and dehydroaltenusin (**346**, Figure 19) and alternariol (**44**), were isolated from *Alternaria alternata* ZHJG5 residing inside the leaves of *Cercis chinensis*. The compounds **44**, **344**, **345**, **346**, showed inhibitory activities on FabH of *X. oryzae* pv. *oryzae* (Xoo) with IC_50_ values ranging from 29.5 to 74.1 μM and also displayed a varying degree of antibacterial activities against *X. oryzae* pv. oryzae (Xoo) with MIC values ranging from 4 to 64 μg/mL. Molecular modeling was then used to picture how these compounds interact with XooFabH. Compounds **44**, and **343**, displayed significant bactericidal activity against rice bacterial leaf blight with a protective efficiency of 66.2 and 82.5% at concentration of 200 μg/mL, respectively [148].

The compound alternariol 9-Me ether (**347**, Figure 19) was purified from *Alternaria alternata* MGTMMP031 associated with *Vitex negundo*. Compound **347** exhibited potential activity against *B. cereus*, *Klebsiella pneumoniae* with a MIC at 30 µM/L. The compound inhibited the growth of *E. coli*, *Salmonella typhi*, *Proteus mirabilis*, *S. aureus* and *S. epidermidis* at a MIC of 35 µM/L [149].

An endophytic fungus, *Alternaria alternata*, associated with *Grewia asiatica* yielded a new structural isomer of alternariol, i.e., 3,7-dihydroxy-9-methoxy-2-methyl-6*H*-benzo[c]-chromen-6-one (**348**, Figure 19), along with alternariol (**44**). Compound **44** inhibited *S. aureus*, VRE, and MRSA with MIC values of 32, 32 and 8 μg/mL, respectively. Compound **348** also inhibited *S. aureus*, VRE, and MRSA with MIC values of 128, 128, and 64 μg/mL, respectively [150].

The compounds 4-hydroxyalternariol-9-methyl ether (**349**, Figure 19) altenuisol (**344**), and alternariol (**44**) were purified from *Alternaria* sp. Samif01, an endophytic fungus of *Salvia miltiorrhiza*. Compounds **44**, **344**, and **349** showed inhibition against *A. tumefaciens*, *B. subtilis*, *P. lachrymans*, *R. solanacearum*, *Staphylococcus hemolyticus* and *Xanthomonas vesicatorya* with MIC values in the range of 86.7–364.7 μM [151]. Previously alternariol 9-Me ether (**347**, Figure 19) was isolated the same fungus and was found active against *B. subtilis*, *S. haemolyticus*, *A. tumefaciens*, *P. lachrymans*, *R. solanacearum*, and *X. vesicatoria* with IC_50_ values ranging from 16.00 to 38.27 g/mL [152].

An endophytic fungus *Alternaria* sp. and *Pyrenochaeta* sp., purified from *Hydrastis canadensis* yielded altersetin (**350**) and macrosphelide A (**351**, Figure 19). Compounds **350** and **351** displayed antibacterial activity against *S. aureus* with MIC values of 0.23 and 75 μg/mL, respectively [153].

#### 2.2.6. *Simplicillium*

The fungal strain *Simplicillium lanosoniveum* associated with *Hevea brasiliensis*, yielded a new depsidone, simplicildone K (**352**), together with the known compounds botryorhodine C (**353**), and simplicildone A (**354**, Figure 19). Compounds **353** and **354** displayed activity against *S. aureus*, MRSA with equal MIC values of 32 μg/mL, whereas **352** exhibited 4-fold less activity against both strains (MIC values of 128 μg/mL) [154].

The compounds botryorhodine C (**353**), and simplicildone A (**354**, Figure 19), were purified from *Simplicillium* sp. PSU-H41 which is associated with the leaves of *Hevea brasiliensis*. Compounds **353** and **354** exhibited poor activity against *S. aureus* (MIC of 32 μg/mL each). Compound **353** was found to be active against MRSA with the same MIC value [155].

#### 2.2.7. *Cladosporium*

**Figure 20:**
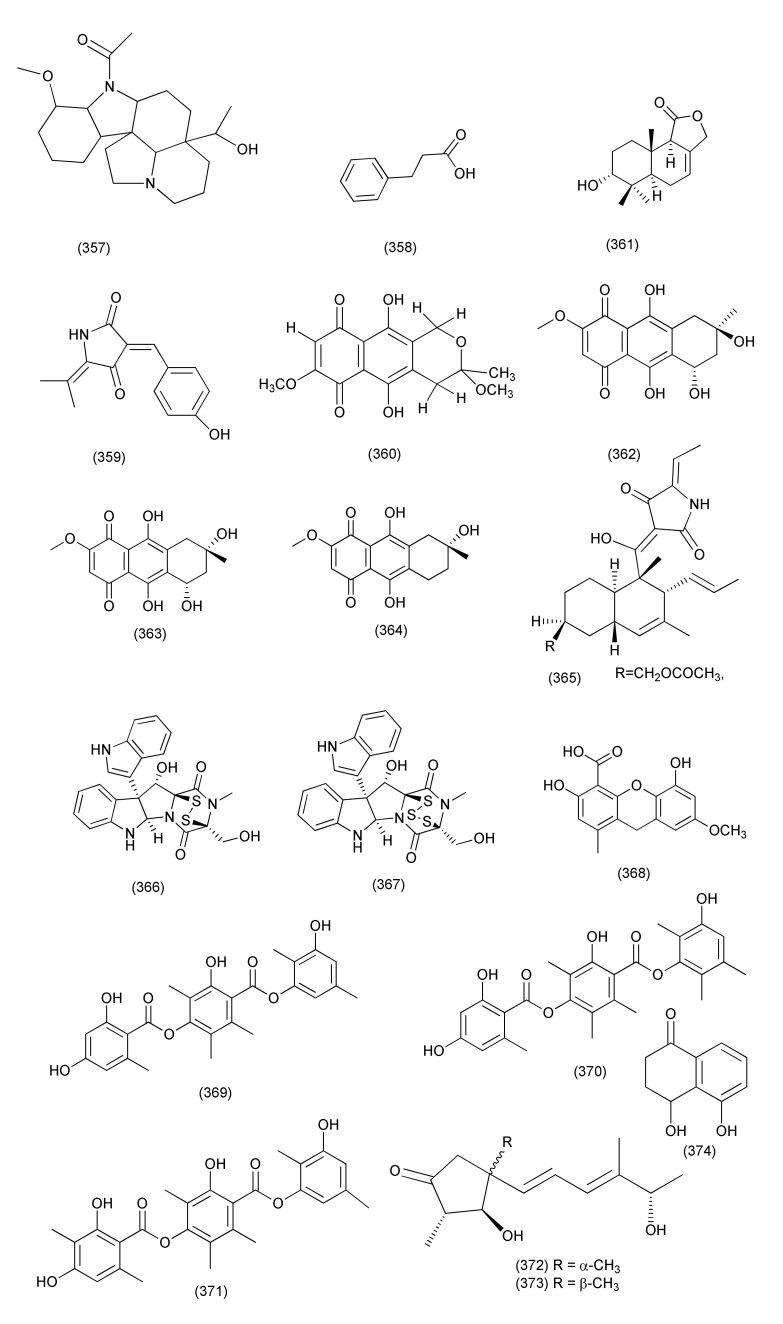
Structures of metabolites **357**–**374** isolated from Anamorphic Ascomycetes.

An endophytic fungus, *Cladosporium cladosporioides*, residing inside the leaves of *Zygophyllum mandavillei* yielded isocladosporin (**355**), 5′-hydroxyasperentin (**356**, Figure 19), 1-acetyl-17-methoxyaspidospermidin-20-ol (**357)**, and 3-phenylpropionic acid (**358**, Figure 20). Compounds **355**–**358** displayed antibacterial activity against *X. oryzae* and *Pseudomonas syringae* with MIC values in the range of 7.81 to 125 µg/mL [156].

A new hybrid polyketide, named cladosin L (**359**, Figure 20) was discovered in the endophytic fungus *Cladosporium sphaerospermum* WBS017 associated with the bulbs of *Fritillaria unibracteata* var. *wabuensis*. Compound **359** inhibited *S. aureus* ATCC 29213 and *S. aureus* ATCC 700699 with MICs of 50 and 25 mM, respectively [157].

A naphthoquinone Me ether of fusarubin (**360**, Figure 20), was purified from a *Cladosporium* sp. associated with the *Rauwolfia serpentina*. Compound **360** (40 μg/disk) displayed potent activity against *S. aureus*, *E. coli*, *P. aeruginosa* and *B. megaterium* with 27, 25, 24 and 22 mm zones of inhibition, respectively and the activities were compared with kanamycin (30 μg/disk) [158].

#### 2.2.8. *Pestalotiopsis*

The genus *Pestalotiopsis* is reported as an endophyte from rain forests in almost all parts of the world and is a prolific producer of chemically diverse bioactive compounds. One such compound is the new drimane sesquiterpenoid 11-dehydro-3a-hydroxyisodrimeninol (**361**, Figure 20), produced by *Pestalotiopsis* sp. M-23, an endophytic fungus of *Leucosceptrum canum*. Compound **361** displayed poor inhibitory effect against *B. subtilis* with IC_50_ value of 280.27 μM [159].

The compounds (1*S*,3*R*)-austrocortirubin (**362**), (1*S*,3*S*)-austrocortirubin (**363**), and 1-deoxyaustrocortirubin (**364**, Figure 20), were obtained from *Pestalotiopsis* sp., an endophyte of *Melaleuca quinquenervia*. Compounds **362–364** displayed with poor antibacterial activity (100 μM) against Gram-positive isolates [160].

A new tetramic acid analog, neopestalotin B (**365**, Figure 20), was extracted from *Neopestalotiopsis* sp. and inhibited *B. subtilis*, *S. aureus*, *S. pneumoniae*, with MIC values of 10, 20, and 20 μg/mL, respectively [161].

#### 2.2.9. *Phoma*

Two known thiodiketopiperazine derivatives **366** and **367** (Figure 20) were purified from *Phoma cucurbitacearum* (now known as *Stagonosporopsis cucurbitacearum*), an endophyte of *Glycyrrhiza glabra*. Compounds **366** and **367** were found to inhibit the battery of bacterial pathogens, including *S. aureus* and *Streptococcus pyogenes* with IC_50_ values of <10 μM. Both compounds potentially inhibited biofilm formation in *S. aureus* and *S. pyogenes* and acted synergistically with streptomycin and inhibited transcription/translation. It was also observed that the sea gene was overexpressed by several fold on treatment with compound **366** while its expression was not affected significantly with compound **367**. The expression of agrA gene was also not affected significantly in *S. aureus* with the treatment of either of the compounds [162].

Barceloneic acid C (**368**, Figure 20), purified from a *Phoma* sp. JS752 residing inside *Phragmites communis*. Compound (**368**) exhibited average antibacterial activities against *Listeria monocytogenes* and *Staphylococcus pseudintermedius*, (MIC of 1.02 μg/mL each) [163].

The polyketides thielavins T (**369**), U (**370**), and V (**371**, Figure 20) were purified from *Setophoma* sp., an endophytic fungus of *Psidium guajava*. Compounds **369**–**371** displayed antibacterial activity against pathogenic *S. aureus* with MIC values of 6.25, 50, and 25 μg/mL, respectively [164].

#### 2.2.10. *Colletotrichum*

**Figure 21:**
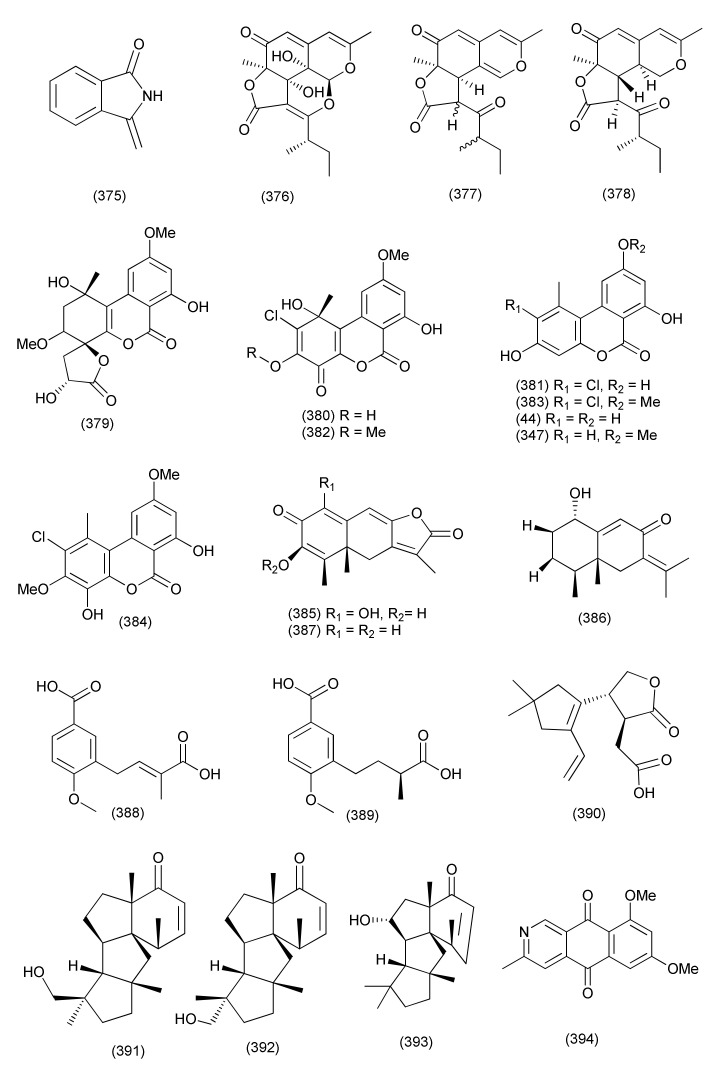
Structures of metabolites **375**–**378** isolated from Anamorphic Ascomycetes and **379**–**394** from Minor Anamorphic Ascomycetes.

Two new γ-butyrolactone derives., colletolides A and B (**372**, **373**), together with the already reported compounds sclerone (**374**, Figure 20), and 3-methyleneisoindolinon (**375**, Figure 21) were purified from *Colletotrichum gloeosporioides* B12, an endophyte of plant *Illigera rhodantha*. Compounds **372**, **373**, **375** were found to be active against *Xanthomonas oryzae* pv. *oryzae*, with the same MIC values of 128 μg/mL, while compound **374** was found active against *X. oryzae* pv. *oryzae* with MIC values of 64 μg/mL [165].

The new compounds colletotrichones A (**376**), B (**377**), and C (**378**, Figure 21) were purified from *Colletotrichum* sp. BS4 residing inside the leaves of *Buxus sinica*. Compound **376** inhibits *E. coli* and *B. subtilis* with MIC values 1.0 and 0.1 μg/mL, respectively. Compound **377** inhibited *S. aureus* with a MIC value of 5.0 μg/mL. Compound **378** has shown antibacterial activity against *E. coli* with a MIC value of 5.0 μg/mL [166].

#### 2.2.11. Minor Taxa of Anamorphic Ascomycetes

New dibenzo-α-pyrones, rhizopycnolide A (**379**), rhizopcnin C (**380**) and rhizopycnin D (**381**), together with known congeners TMC-264 (**382**), palmariol B (**383**) penicilliumolide D (**384**, Figure 21) alternariol 9-methyl ether (**347**) and alternariol (**44**) and were purified from *Rhizopycnis vagum* (now known as *Acrocalymma vagum*) isolated from *Nicotiana tabacum*. Compounds **380**, **384**, **44** inhibited *A. tumefaciens*, *B. subtilis*, *Pseudomonas lachrymans*, *R. solanacearum*, *Staphylococcus hemolyticus*, and *Xanthomonas vesicatoria*, with MICs in the 25−100 μg/mL range. Rhizopycnolide A (**379**) was active against *A. tumefaciens*, *B. subtilis*, and *P*. *lachrymans*, with MIC values of 100, 75, and 100 μg/mL, respectively. Rhizopycnin D (**381**) was found to be active against *A. tumefaciens*, *B. subtilis*, and *R. solanacearum*, with an equal MIC value of 50 μg/mL, and against *X. vesicatoria*, with a MIC value of 75 μg/mL. TMC-264 (**382**) was selectively active against *B. subtilis* (MIC value of 50 μg/mL). Compounds **383** and **347** inhibited *A. tumefaciens*, *B. subtilis*, *P. lachrymans*, *R. solanacearum*, and *X. vesicatoria*, with IC_50_ values in the range 16.7−34.3 μg/mL [167].

Rhizoperemophilane K (**385**), 1α-hydroxyhydroisofukinon (**386**) and 2-oxo-3-hydroxy-eremophila-1(10),3,7(11),8-tetraen-8,12-olide (**387**, Figure 21) were purified from *Rhizopycnis vagum* (now known as *Acrocalymma vagum*), an endophyte of *Nicotiana tabacum*. Compounds **385, 386** and **387** displayed inhibition against *A. tumefaciens*, *B. subtilis*, *P. lachrymans*, *Ralstonia solanacearum*, *S. haemolyticus*, and *X. vesicatoria*, with MIC values in the range of 32~128 μg/mL [168].

Rhizopycnis acids A (**388**) and B (**389**, Figure 21), were purified from *Rhizopycnis vagum* (now known as *Acrocalymma vagum*) an endophyte of *Nicotiana tabacum* from China Agricultural University (Beijing, China). Compound **388** inhibited *A. tumefaciens*, *B. subtilis*, *P. lachrymans*, *R. solanacearum*, *S. hemolyticus* and *X. vesicatoria* with MIC values of 20.82, 16.11, 23.48, 29.46, 21.11, and 24.31 µg/mL, respectively. Compound **389** also inhibited *A. tumefaciens*, *B. subtilis*, *P. lachrymans*, *R. solanacearum*, *S. haemolyticus,* and *X. vesicatoria* with MIC values of 70.89, 81.28, 21.23, 43.40, 67.61, and 34.86 µg/mL, respectively [169].

*Leptosphaeria* sp. XL026 associated with *Panax notoginseng* yielded a new sesquiterpenoids, leptosphin B (**390**), along with three known diterpenes, conidiogenone C (**391**), conidiogenone D (**392**) and conidiogenone G (**393**, Figure 21). The site of the collection was Shijiazhuang (Hebei Province, China). Compounds **390**–**393** showed average antibacterial activity against *B. cereus*, with MIC values of 12.5–6.25 μg/mL and compound **392** also showed antibacterial activity against *P. aeruginosa* with a MIC value of 12.5 μg/mL [170].

**Figure 22:**
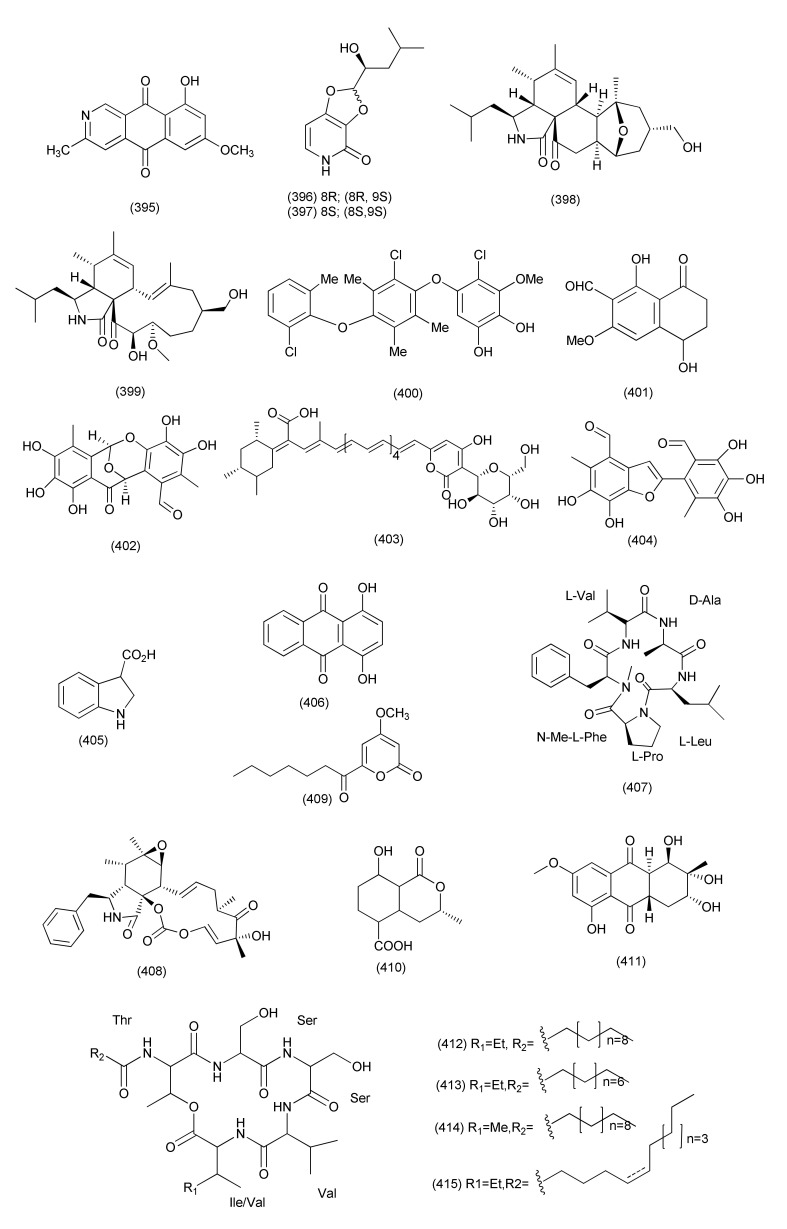
Structures of metabolites **395**–**415** isolated from Minor Anamorphic Ascomycetes.

Two 2-azaanthraquinones, scorpinone (**394**, Figure 21) and 5-deoxybostrycoidin (**395**, Figure 22), were purified from *Lophiostoma* sp. Eef-7 is associated with *Eucalyptus exserta*. Compounds **394** and **395** displayed poor antibacterial activity against *Ralstonia solanacearum* with 9.86 and 9.58 mm zones of inhibition when 64 µg was added (positive control was streptomycin sulfate with a 13.03 mm zone of inhibition at an added amount of 6.25 µg) [171].

Two new cytochalasan alkaloids, cytochrysins A and C (**396** and **397**, Figure 22), were isolated from *Cytospora chrysosperma*, an endophytic fungus isolated from *Hippophae rhamnoides*. Compound **396** showed significant antibacterial activity against multi-drug resistant *Enterococcus faecium* with MIC value of 25 μg/mL, and compound **397** was active against MRSA with a MIC value of 25 μg/mL [172].

Two known α-pyridones, (8*R*,9*S*)-dihydroisoflavipucine (**398**) and (8*S*,9*S*)-dihydroisoflavipucine (**399**, Figure 22) were isolated from *Lophiostoma* sp. Sigrf10 is associated with *Siraitia grosvenorii*. Compounds **398** and **399** were active against *B. subtilis*, *A. tumefaciens*, *R. solanacearum*, and *X. vesicatoria*, with IC_50_ values in the range of 35.68–44.85 µM [173].

Microsphaerol (**400**), a novel polychlorinated triphenyl diether was extracted from *Microsphaeropsis* sp and seimatorone (**401**, Figure 22), a new naphthalene derivative, was purified from the endophyte *Seimatosporium* sp. Compound **400** displayed potent antibacterial activity against *B. megaterium* and *E. coli*, with 8 and 9 mm zones of inhibition at 0.05 mg concentration (50 mL of 1 mg/mL). Compound **401** exhibited moderate antibacterial activity against *B. megaterium* and *E. coli*, with 3 and 7 (partial inhibition) mm zones of inhibition at a 0.05 mg concentration (50 mL of 1 mg/mL) [174].

Known compounds epicocconigrone A (**402**), epipyrone A (**403**), and epicoccolide B (**404**, Figure 22) were purified from *Epicoccum nigrum* MK214079 associated with *Salix* sp. Compounds **402**–**404** exhibited moderate activity against *S. aureus*, with MICs ranging from 25 to 50 μM [175].

The known compounds *p*-hydroxybenzaldehyde (**223**), indole-3-carboxylic acid (**405**) and quinizarin (**406**, Figure 22) and beauvericin (**267**), were isolated from *Epicoccum nigrum* associated with the *Entada abyssinica*. Compound **267** displayed activity against *S. aureus*, *B. cereus*, and *Salmonella typhimurium*, with MIC values of 3.12, 12.5, and 12.5 µg/mL. Compounnd (**223**) displayed activity against *S. aureus*, *B. cereus*, *P. aeruginosa*, and *E. coli* with MIC values of 50, 25, 50, and 25 µg/ml. Compound **405** was found to be active against *S. aureus* and *E. faecalis* (MICs of 6.25 and 50 µg/mL) while compound **406** displayed activity against *S. aureus*, *B. cereus* St (MICs of 50 µg/mL each) [176].

The endophytic fungus *Stemphylium lycopersici* from *S. tonkinensis* yielded xylapeptide B (**407**), cytochalasin E (**408**), 6-heptanoyl-4-methoxy-2*H*-pyran2-one (**409**) and (–)-5-carboxymellein (**410**, Figure 22). Compound **407** showed average inhibition against *B. subtilis* with a MIC value of 12.5 μg/mL, and against *S. aureus and E. coli* with MIC values of 25 μg/mL. Compound **408** inhibited *B. subtilis*, *S. aureus*, *B. anthracis*, *S. dysenteriae*, and *E. coli* with MIC values ranging from 12.5 to 25 μg/mL. Compound **409** inhibited *S. paratyphi* B with MIC value of 12.5 μg/mL. Compound **410** inhibited *B. subtilis*, *S. aureus*, *B. anthracis*, *S. dysenteriae*, *S. paratyphi*, *E. coli* and *S. paratyphi* B with MIC values ranging from 12.5 to 25 μg/mL [177].

A new tetrahydroanthraquinone derivative, dihydroaltersolanol C (**411**, Figure 22) was purified from *Stemphylium globuliferum* residing inside the plant *Juncus acutus*. Compound **411** exhibited moderate growth inhibition effects against *S. aureus* with a MIC of 49.7 μM [178].

An endophytic fungus *Lecanicillium* sp. (BSNB-SG3.7 Strain) associated with *Sandwithia guyanensis* yielded stephensiolides I (**412**), D (**413**), G (**414**), and stephensiolide F (**415**, Figure 22). Compounds **412**–**415** displayed anti-MRSA activity with MIC values of 4, 32, 16 and 32 μg/mL, respectively [179].

**Figure 23:**
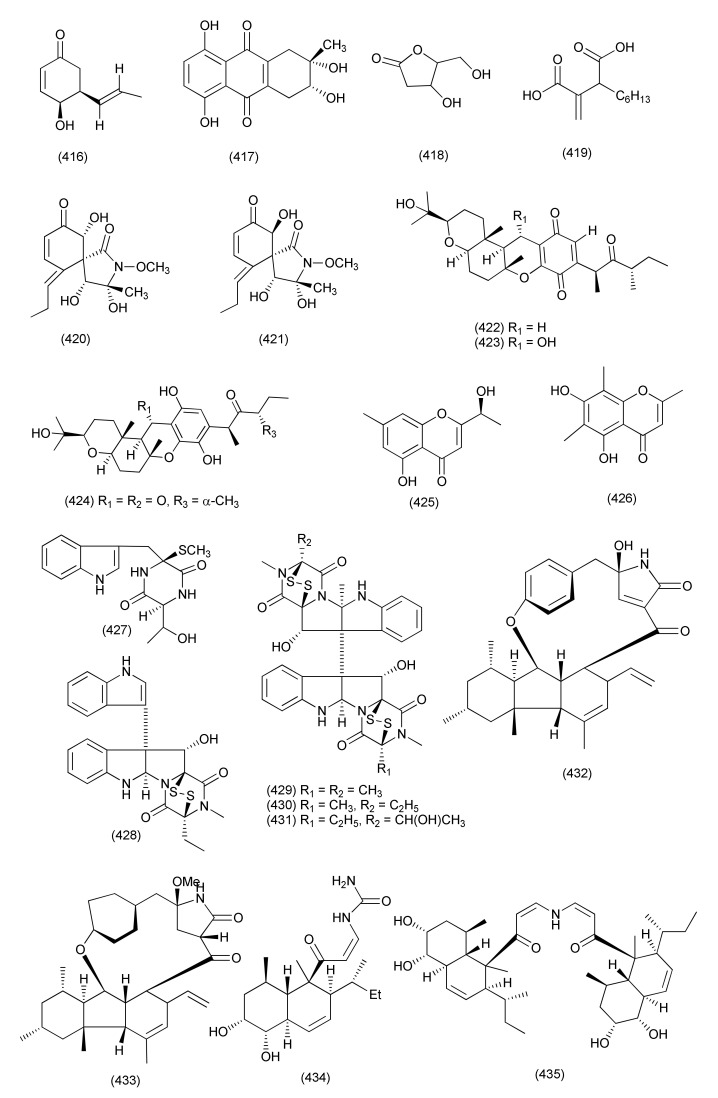
Structures of metabolites **416**–**435** isolated from Minor Anamorphic Ascomycetes.

The compound phomalactone (**416**, Figure 23) was isolated from the endophyte *Nigrospora sphaerica* associated with *Adiantum philippense*. Compound **416** displayed good antibacterial activity against *E. coli* and *X. campestris* with MIC values of 3.12 μg/mL and moderate activity against *S. typhi*, *B. subtilis*, *B. cereus*, and *K. pneumonia* with a MIC value of 6.25 μg/mL. A MIC of 12.5 μg/mL was found against *S. aureus*, and *S. epidermidis* [180].

A new naturally occurring compound, nigrosporone B (**417**, Figure 23), was purified from *Nigrospora* sp. BCC 47789 associated with the leaves of *Choerospondias axillaris.* Compound **417** exhibited antibacterial activity against *M. tuberculosis*, *B. cereus* and *E. faecium* with MIC values of 172.25, 21.53 and 10.78 μM, respectively [181].

Two bioactive compounds, 2′-deoxyribolactone (**418**) and hexylitaconic acid (**419**, Figure 23) were purified from *Curvularia sorghina* BRIP 15900 associated with the stem bark of *Rauwolfia macrophylla*. Compounds **418** and **419** inhibited *Staphylococcus warneri E. coli*, *Pseudomonas agarici* and *Micrococcus luteus*, with MICs ranging between 0.17 μg/mL and 0.58 μg/mL [182].

Known compounds, namely the triticones E (**420**) and F (**421**, Figure 23), were purified from *Curvularia lunata*, isolated from healthy capitula of *Paepalanthus chiquitensis*. Compounds **420** and **421** showed good antibacterial activity for *E. coli*, with MIC values of 62.5 μg/mL [183].

The known compounds cochlioquinones B (**422**), C (**423**), and isocochlioquinone C (**424**, Figure 23) were purified from *Bipolaris* sp. L1-2 which is associated with the leaves of *Lycium barbarum*. Compounds **422**–**424** showed antimicrobial activity against *B. subtilis*, *C. perfringens*, and *P. viridiflava*, with MICs of 26 μM [184].

A new previously undescribed chromone, (*S*)-5-hydroxyl-2-(1-hydroxyethyl)-7-methylchromone (**425**) and the known sativene-type sesquiterpenoid 5,7-dihydroxy-2,6,8-trimethylchromone (**426**, Figure 23), were purified from *Bipolaris eleusines* associated with potatoes from Yunnan Agricultural University (Kunming, Yunnan, China). Compounds **425** and **426** displayed poor inhibitory activities against *S. aureus* sub sp. *aureus* with the inhibition rates of 56.3 and 32 %, respectively, at the concentration of 128 μg/mL (penicillin G: 99.9% at 5 μg/mL) [185].

Two new diketopiperazines, bionectin D (**427**) and bionectin E (**428**) and the known compounds verticillin A (**429**) sch 52901 (**430**) and gliocladicillin C (**431**, Figure 23) were purified from *Bionectria* sp. Y1085, isolated from *Huperzia serrata*. Bionectin D (**427**) is a rare diketopiperazine with a single methylthio substitution at the α-carbon of a cyclized amino acid residue. Compounds **427**–**331** exhibited antibacterial activity against *E. coli*, *S. aureus,* and *S. typhimurium*, with MIC values ranging from 6.25–25 µg/mL [186].

Known compounds pyrrocidine A (**432**) and 19-O-methylpyrrocidine B (**433**, Figure 23) were extracted from the endophytic fungus, *Cylindrocarpon* sp., isolated from *Sapium ellipticum*. Compound **433** exhibited moderate antibacterial activity against *S. aureus* ATCC 25923 and ATCC 700699 with MIC values of 50 and 25 μM, respectively. Compound **432** showed strong to moderate inhibitory effects against *S. aureus* strain ATCC 25923 and ATCC 700699, *E. faecalis* strain ATCC 29212 and ATCC 51299, *E. faecium* strain ATCC 35667 and ATCC 700221 with MIC values ranging from 0.78 to 25 μM [187].

**Figure 24:**
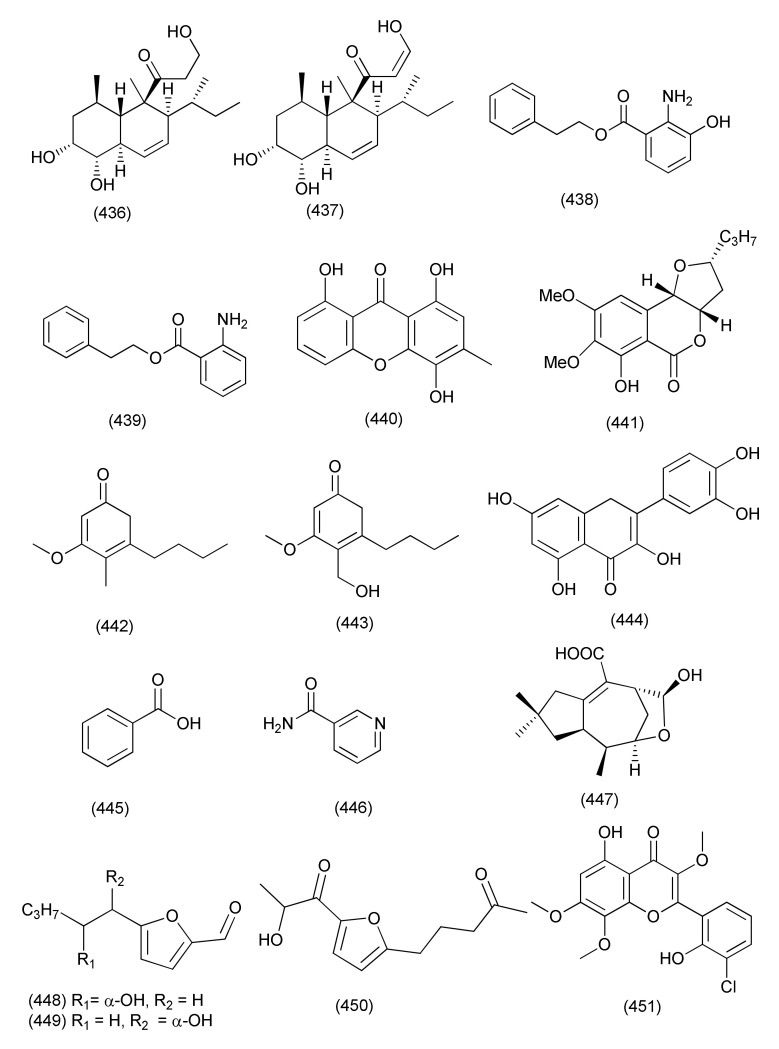
Structures of metabolites **436**–**443** isolated from Minor Anamorphic Ascomycetes, **444**–**450** from Basidiomycetes and **451** from Zygomycetes.

Two new decalin-containing compounds, eupenicinicols C (**434**), and D (**435**, Figure 23), along with two biosynthetically-related known metabolites, eujavanicol A (**436**), and eupenicinicol A (**437**, Figure 24) were obtained from *Eupenicillium* sp. LG41.9 (now considered as *Penicillium*) residing inside the roots of *Xanthium sibiricum* when treated with the HDAC inhibitor nicotinamide (15 mg/100 mL). Compound **435** exhibited pronounced efficacy against *S. aureus* with a MIC of 0.1 μg/mL, and compound **436**, was active against *E. coli* with a MIC of 5.0 μg/mL [188].

A new anthranilic acid derivative, 2-phenylethyl 3-hydroxyanthranilate (**438**) and 2-phenylethyl anthranilate (**439**, Figure 24) were extracted from *Dendrothyrium variisporum* extracted from the roots of *Globularia alypum*. Metabolite **438** was found to be active against *B. subtilis* and *M. luteus* (MICs of 8.33 and 16.66 μg/mL). Compound **439** showed potent activity against *B. subtilis* and *S. aureus* with MIC values of 66.67 μg/mL each [189].

Ravenelin (**440**, Figure 24) was extracted from *Exserohilum rostratum*, an endophyte of *Phanera splendens*, an endemic medicinal plant of the Amazon region. Ravenelin (**440**) displayed antibacterial activity against *B. subtilis* and *S. aureus* with MIC values of 7.5 and 484 μM, respectively (amoxicillin MIC against *B. subtilis* and *S. aureus* 1.3 and 21.4 μM; another positive control terramycin MIC against *B. subtilis* and *S. aureus* 16.3 and 16.3 μM, respectively) [190].

The compounds monocerin (**441**), annularin I (**442**), and annularin J (**443**, Figure 24) were purified from *Exserohilum rostratum* isolated from *Bauhinia guianensis*. Compound **441** displayed antibacterial activity with MIC values of 62.5 µg/mL against *P. aeruginosa*. Compound **442** exhibited antibacterial activity with MIC values of 62.50 and 31.25 µg/mL against *E. coli* and *B. subtilis*, respectively. Compound **443** displayed weak activity against *E. coli* and *B. subtilis* with MIC values of 62.50 µg/mL each [191].

### 2.3. Basidiomycetes

The compounds quercetin (**444**), carboxybenzene (**445**), and nicotinamide (**446**, Figure 24) were purified from *Psathyrella candolleana* residing inside the seeds of *Ginkgo biloba*. Compounds **444–446** have antibacterial activity against *S. aureus* (MIC 0.3906, 0.7812 and 6.25 μg/mL) [192].

A new tremulane sesquiterpene, irpexlacte A (**447**), and three new furan derivatives, irpexlactes B-D (**448**–**450**, Figure 24), were isolated from the endophytic fungus *Irpex lacteus* DR10-1 of the waterlogging-tolerant plant *Distylium chinense*. Compounds **447**–**450** showed moderate antibacterial activity against *P. aeruginosa* with MIC values ranging from 23.8 to 35.4 μM [193].

### 2.4. Zygomycetes

A flavonoid compound, chlorflavonin (**451**, Figure 24) was purified from the endophytic fungus *Mucor irregularis*, isolated from *Moringa stenopetala*. It has shown antibacterial activity (MIC_90_) against *M. tuberculosis* at a 1.56 μM concentration. Chlorflavonin also had shown synergistic effects with isoniazid and delamanid in combination treatment experiments. Various molecular and docking techniques have shown that chlorflavonin interacts with the acetohydroxyacid synthase catalytic subunit IlvB1 and inhibits their activity. Recently, Rehberg et al. [194] found the antimicrobial activity of chlorflavonin (**451**) to be higher in comparison to streptomycin treatment against macrophages infected with *M. tuberculosis*.

## 3. Volatile Organic Compounds (VOCs)

Volatile organic compounds (VOCs) are chemical entities which have low molecular weights and typically evaporate or get into the vapor phase at normal temperature and pressure. They generally possess a characteristic odor [195]. Several reviews have emphasized the production of biogenic VOCs as possible signal molecules in the course of interaction with a host or that play a role in the process of host integration. At times they are also identified as indicators of fungal growth [196,197,198]. Fungal VOCs largely comprise aliphatic as well as aromatic hydrocarbons, aldehydes, mono-, di- and sesquiterpenes, esters and ketones. Some of the interesting aspects of fungal volatiles is their possible role during interactions among the microbes i.e., with bacteria as well as fungi. However, the application of fungal VOCs as an arsenal to kill bacteria and fungi has not been extensively explored.

The discovery of the endophytic fungus *Muscodor albus* Cz 620 which exhibited potent antibiotic type activity, wiping out all the microbes in its vicinity was serendipitous. This was attributed due to the volatile cocktail produced by *Muscodor albus* Cz 620. This marked the beginning of the exploration of fungal endophytes with the potential to produce volatile antibiotics. The genus *Muscodor* has expanded in the last two decades owing to the addition of novel members that were largely based on the chemical signatures and genetic profiles. Presently there are ~22 known type species that have been documented [199]. Uniquely, all the species of *Muscodor* reported to date are sterile in nature and exhibit a characteristic spectrum of antibacterial as well as anti-fungal activities largely driven by the chemical composition of their volatile gas mixtures. It has also been shown that a single component of the volatile gas is unable to mimic the anti-microbial action suggesting it to be a synergistic action of the finely tuned composition of different VOCs [200]. The pharmaceutical importance of the VOCs produced by *Muscodor* species was exemplified by the anti-bacterial and anti-fungal potential of the VOCs emitted by the fungus. VOCs of *Muscodor albus* Cz620 inhibited *E. coli* and *Bacillus subtilis* while only *E. coli* was inhibited in the presence of volatiles of other isolates of *Muscodor albus* viz. KN-26, KN-27, GP-100, GP-115, TP-21, which inhibited only *E. coli* [201]. The volatiles of *M. albus* I-41.3s on the other hand inhibited *Bacillus subtilis*, *E. coli*, and *Salmonella typhi*. All the VOC emissions were predominantly bacteriostatic and not bactericidal [202].

*Muscodor crispans* (B-23) has a characteristic VOC spectrum which exhibited anti-mycobacterial activity i.e., against *Mycobacterium marianum* apart from *S. aureus* ATCC6538, *Salmonella cholereasus*, and *Yersinia pestis* [203]. *Muscodor fengyangensis* exclusively inhibited *E. coli* [204]. The volatiles produced by *Muscodor kashayum* has a potent bactericidal activity towards *E. coli, Pseudomonas aeruginosa, Salmonella typhi* and *S. aureus* [205]. Four isolates of *Muscodor* reported from Southeast Asia, viz. *M. oryzae*, *M. musae*, *M. suthepensis* and *M. equisetii*, exerted bactericidal activity against *Enterococcus faecalis*, *E. coli*, *Proteus mirabilis*, *S. aureus* and *Pseudomonas pneumoniae* [206]. The VOCs of *Muscodor* have also inspired development of a veterinary medicine formulation which is used as an anti-diarrhoeal product. The formulation is called Sx calf, that is currently being produced and marketed by Ecoplanet Environment LLC (Belgrade, MT, USA) [207]. Similarly, the volatiles of *Muscodor cinnamomi* was found to be effective against *Staphylococcal* spp., *Salmonella* sp., *E. coli, Klebsiella* spp., *Streptococcus* spp. and *Enterococcus* species which contaminate eggs thereby not only affecting their shelf life but also making them unfit for human consumption [208]. The volatile cocktail of *Muscodor crispans* (B-23) was found to kill the bacterial pathogen of citrus *Xanthomonas axonopodis* pv. *citri* [203].

The introspection of the spectrum of the volatile organic mixture from different *Muscodor* species has revealed the antibacterial spectrum of some commonly occurring entities such as isobutyric acid [209,210,211], β-bisabolol and azulene and its derivatives [212]. Thus, creating artificial mixtures and evaluating them for their anti-bacterial activities may prove to be very useful for preventing drug-resistant film-forming bacteria from causing infections in clinical as well as non-clinical settings. Hence the present study, opens avenues to explore higher numbers of fungal endophytes for their unique volatile signatures and assess them for anti-bacterial activities for developing interventions that could check the spread and infections caused by the drug-resistant bacteria by using them in volatile form or as gaseous sprays.

**Table 1:** Anti-bacterial metabolites reported from endophytic fungi.

Sr. No.	Fungus	Source	Locality	Compounds Isolated	Biological Target	Biological Activity (MIC/IC_50_/ID_50_)	Reference
**Ascomycetes**							
*Diaporthe*							
1	*Diaporthe* sp.	*Uncaria gambier*		(+)-1,1′-Bislunatin (**1**) and (+)-2,2′- epicytoskyrin A (**2**)	*Mycobacterium tuberculosis* strains H37Rv	MICs 0.422 and 0.844 μM	[18]
2	*Diaporthe* sp. GDG-118	*Sophora tonkinensis*	Hechi City, China	21-Acetoxycytochalasin J_3_ (**3**)	*Bacillus anthraci* and *E. coli*	inhibited at 12.5 μg/mL concentration	[19]
3	*Phomopsis fukushii*.			1-(3-Hydroxy-1-(hydroxymethyl)-2-methoxy-6-methylnaphthalen-7-yl) propan-2-one (**4**) and 1-(3-hydroxy-1- (hydroxymethyl)-6-methylnaphthalen-7-yl)propan-2-one (**5**)	MRSA	Zone of inhibition of 10.2 and 11.3 mm (6 mm strile filterpaper disc were impregnated with 20µL (50 µg) of each compound)	[20]
4	*Phomopsis fukushii*	*Paris polyphylla* var. *yunnanensis*	Kunming, Yunnan, China	3-Hydroxy-1-(1,8- dihydroxy- 3,6-dimethoxynaphthalen-2-yl)propan-1-one (**6**), 3-hydroxy-1-(1,3,8-trihydroxy-6-methoxynaphthalen-2-yl)propan-1-one (**7**) and 3-hydroxy-1-(1,8-dihydroxy3,5-dimethoxynaphthalen-2-yl) propan-1-one (**8**)	MRSA- ZR11	MIC, 8, 4, and 4 µg/mL,	[21]
5	*Phomopsis fukushii*	*Paris polyphylla* var. *yunnanensis*	Kunming, Yunnan, China	1-[2-Methoxy-4-(3-methoxy-5-methylphenoxy)-6-methylphenyl]-ethanone (**9**) and 1-[4-(3-(hydroxymethyl)-5-methoxyphenoxy)-2-methoxy-6-methylphenyl]-ethanone (**10**)	MRSA	Zone of inhibition 13.8 and 14.6 mm	[22]
6	*Phomopsis fukushii*	*Paris polyphylla* var. *yunnanensis*	Kunming, Yunnan, P. R. China	4-(3-Methoxy-5-methylphenoxy)-2-(2-hydroxyethyl)-6-methylphenol (**11**), 4-(3-Hydroxy-5-methylphenoxy)-2-(2-hydroxyethyl)-6-methylphenol (**12**) and 4-(3-methoxy-5-methylphenoxy)-2-(3-hydroxypropyl) -6-methylphenol (**13**)	MRSA	Zone of inhibition of 20.2, 17.9 and 15.2 mm (tested at 50µg/6 mm disc)	[23]
7	*Phomopsis fukushii*	*Paris polyphylla* var. *yunnanensis*	Kunming, Yunnan, China.	1-(4-(3-Methoxy-5-methylphenoxy)-2-methoxy-6-methylphenyl)-3-methylbut-3-en-2-one (**14**), 1-(4-(3-(hydroxymethyl)-5-methoxyphenoxy)-2-methoxy-6- methylphenyl)-3-methylbut-3-en-2-one (**15**), 1-(4-(3-hydroxy-5-(hydroxymethyl)phenoxy)-2-methoxy-6- methylphenyl)-3-methylbut-3-en-2-one (**16**)	MRSA	Zone of inhibition of 21.8, 16.8 and 15.6 mm, (50 µg/6 mm disc)	[24]
8	*Phomopsis* sp.	*-*	-	3-Hydroxy-6-hydroxymethyl-2,5-dimethylanthraquinone (**17**), 6-hydroxymethyl-3-methoxy-2,5-dimethylanthraquinone (**18**)	MRSA	IZD 14.2 and 14.8 mm	[25]
9	*Diaporthe* sp.	*Pteroceltis tatarinowii*	Mufu Mountain of Nanjing, China.	Diaporone A (**19**)	*B. subtilis*	MIC, 66.7 μM,	[26]
10	*Phomopsis prunorum* (F4-3).	*-*	-	(−)-1 and (+)- Phomoterpenes A and B (**20**) phomoisocoumarins C (**21**), D (**22**)	*X. citri* pv. *phaseoli* var. *fuscans*	MIC, 31.2, 62.4, 31.2, and 31.2 μg/mL,	[27]
					*Pseudomonas syringae* pv. *Lachrymans*	MIC, 31.2, 15.6, 31.2 and 15.6 μg/mL	
11	*Diporthe vochysiae* LGMF1583	*Vochysia divergens*	-	Vochysiamides A (**23**)	KPC (*Klebsiella pneumoniae* carbapenemase producing).	MIC, 1.0 μg/mL	[28]
				Vochysiamides B (**24**)	KPC, MSSA, MRSA	MIC, 0.08, 1.0, and 1.0 µg/mL	
12	*Phomopsis asparagi*	*Paris polyphylla* var. *yunnanensis*	Kunming, Yunnan, China	4-(3-Methoxy-5-methylphenoxy)-2-(2-hydroxyethyl)- 6-(hydroxymethyl)phenol (**25**), 4-(3-Hydroxy-5-methylphenoxy)-2-(2-hydroxyethyl)-6-(hydroxymethyl)phenol(**26**)	MRSA	Zone of inhibition of 10.8 and 11.4 mm	[29]
13	*Phomopsis* sp.	*Paris polyphylla* var. *yunnanensis*	ShiZhong, Yunnan, China	5-Methoxy-2-methyl-7-(3-methyl-2-oxobut-3-enyl)-1-naphthaldehyde (**27**), 2-(hydroxymethyl)-5-methoxy-7-(3-methyl-2-oxobut-3-enyl)-1-naphthaldehyde (**28**)	MRSA	Zone of inhibition of 14.5 and 15.2 mm	[30]
14	*Diaporthe terebinthifolii* LGMF907	*Schinus terebinthifolius*	Curitiba, Paraná, Brazil	Diaporthin (**29**)	*E. coli*, *Micrococcus luteus*, MRSA, and *S. aureus*	Zone of inhibition 1.73, 2.47, 9.50, and 9.0 mm tested at 100 μg/disk.	[31]
				Orthosporin (**30**)		Zone of inhibition of 1.03, 1.53C, 9.0, and 9.33 mm	
15	*Phomopsis*/*Diaporthe* sp. GJJM 16	*Vitex negundo*	Azhiyar, Pollachi, Tamilnadu, India	(2*Z*)-2-(1,4-dihydro-2-hydroxy-1-((*E*)-2-mercapto-1 (methylimino)ethyl) pyrimidine-4-ylimino)-1-(4,5-dihydro-5-methylfuran-3-yl)-3-methylbutane-1-one (**31**)	*S. aureus*, and *P. aeroginosa*	MIC of 1.25 μg/mL against each organism	[32]
16	*Phomopsis* sp. PSU-H188	*Hevea brasiliensis*	Trang Province, Thailand.	Diaporthalasin (**32**)	*S. aureus* ATCC25923, MRSA	MIC, 4 μg/mL each	[33]
				Cytosporone B (**33**)		MIC, 32 and 16 μg/mL	
				Cytosporone D (**34**)		MIC, 64 and 32 μg/mL	
17	*Diaporthe terebinthifolii* GG3F6	*Glycyrrhiza glabra*	Jammu, J & K, India	Diapolic acid A (**35**), B (**36**) xylarolide (**37**) phomolide G (**38**)	*Yersinia enterocolitica*	IC_50_, 78.4, 73.4, 72.1 and 69.2 μM	[34]
18	*Diaporthe* sp. F2934	leaves of *Siparuna gesnerioides*	Chagres National Park, a protected area of Panama	Phomosine A (**39**)	*S. aureus* (ATCC 25923), *Streptococcus oralis* (ATTC 35037), *Enterococcus faecalis* (ATCC 19433), *Enterococcus cloacae* (ATCC 13047), *Bordetella bronchiseptica* (CECT 440),	Zone of Inhibition 12, 9, 10, 11, 10 and 10 mm at 4 µg/mL concentration	[35]
				Phomosine C (**40**)		Zone of Inhibition 9, 6, 8, 8, 8 and 9 mm at 4 µg/mL concentration	
19	*Phomopsis* sp.,	*Garcinia kola* nuts	bought at Mokolo local market in Yaounde (Cameroon)	18-Methoxycytochalasin J (**41**), cytochalasins H (**42**) and J (**43**), alternariol (**44**)	*Shigella flexneri*	MIC, 128 μg/mL each	[36]
				18-Methoxycytochalasin J (**41**), cytochalasins H (**42**)	*S. aureus* ATCC 25923	MIC, 128 and 256 μg/mL	
20	*Diaporthe* sp. LG23	*Mahonia fortunei*	Shanghai, China	19-nor-Lanosta-5(10),6,8,24-tetraene-1α,3β,12β,22*S*-tetraol (**45**)	*S. aureus*, *E. coli*, *Bacillus subtilis*, *P. aeruginosa*, *Streptococcus pyogenes*	MIC, 5.0, 5.0, 2.0, 2.0 and 0.1 µg/mL	[37]
				3β,5α,9α-Trihydroxy-(22*E*,24*R*)-ergosta-7,22-dien-6-one (**46**), and chaxine C (**47**)	*B. subtilis*	MIC, 5.0 µg/mL each	
21	*Diaporthales* sp. E6927E	*Ficus sphenophyllum*	Ecuadorean dry forest near the Napo River, USA	Pyrrolocin A (**48**)	*S. aureus* and *E. faecalis*	MICs 4 and 5 µg/mL	[38]
	*Xylaria*						
22	*Xylaria ellisii*	Blueberry (*Vaccinium angustifolium*)		Ellisiiamide (**49**)	*Escherichia coli*	MIC, 100 μg/mL	[39]
23	*Xylaria* sp. GDG-102	*S. tonkinensis*	Hechi, Guangxi province, China	Xylareremophil (**50**)	*Micrococcus luteus* and *Proteus vulgaris*	MIC 25 μg/mL each	[40]
				Mairetolides B (**51**)	*M. luteus*	MIC, 50 μg/mL	
				Mairetolide G (**52**)	*P. vulgaris M. luteus*	MIC 25 and 50 μg/mL	
				Xylareremophil (**50**), mairetolides B (**51**), and G (**52**)	*Micrococcus lysodeikticus* and *Bacillus subtilis*	MIC 100 μg/mL	
24	*Xylaria* sp. (GDG-102)	Leaves of *S. tonkinensis*		6-Heptanoyl-4-methoxy-2H-pyran-2-one (**53**)	*E. coli* as well as *S. aureus*	MIC, 50 μg/mL	[41]
25	*Xylaria* sp. GDG-102	*S. tonkinensis*	Hechi, Guangxi province, China	Xylarphthalide A (**54**)	*B. subtilis* and *E. coli*,	MIC, 12.5 μg/mL each	[42]
					*B. megaterium*, *S. aureus*, *S. dysenteriae* and *S. paratyphi*	MIC, 25 μg/mL each	
				(−)-5-Carboxymellein (**55**)	*B. Subtilis*	MIC, 12.5 μg/mL	
					*B. anthracis*, *B. megaterium*, *S. aureus*, *E. coli*, *S. dysenteriae* and *S. paratyphi* B	MIC, 25 μg/mL	
				(−)-5-Methylmellein (**56**)	*B. subtilis* and *S. aureus*	MIC, 12.5 μg/mL	
					*B. megaterium*, *E. coli* and *S. dysenteriae*	25 μg/mL	
26	*Xylaria* sp.,	*Taxus mairei*.		3,7-Dimethyl-9-(-2,2,5,5-tetramethyl-1,3-dioxolan-4-yl) nona-1,6-dien-3-ol (**57**)	*B. subtilis* ATCC 9372, *B. pumilus* 7061 and *S. aureus* ATCC 25923	48.1, 31.6 and 47.1% inhibition.	[43]
				Nalgiovensin (**58**)	*S. aureus* ATCC 25923, *B. subtilis* ATCC 9372, *B. pumilus* ATCC 7061 and *E. coli* ATCC 25922	42.1, 36.8, 47.1 and 41.2% inhibition.	
	***Chaetomium***						
27	*C. globosum* 7s-1,	*Rhapis cochinchinensis*		Xanthoquinodin B9 (**59**), xanthoquinodin A1 (**60**), xanthoquinodin A3 (**61**)	*B. cereus*	MICs of 0.87, 0.44 and 0.22 μM,	[45]
				Xanthoquinodin B9 (**59**), xanthoquinodin A1 (**60**), xanthoquinodin A3 (**61**)	*S. aureus* and MRSA	MIC values ranging from 0.87 to 1.75 μM	
				3-Epipolythiodioxopiperazines, chetomin (**62**), chaetocochin C (**63**) and dethio-tetra(methylthio)chetomin (**64**)	*B. cereus* ATCC 11778, *S. aureus* ATCC 6538, and MRSA	MIC values ranging from 0.02 pM to 10.81 μM.	
				Chetomin (**62**)	*B. cereus*, *S. aureus* and MRSA	MICs, 0.35 μM, 10.74 and 0.02 pM	
				Compounds **59**–**64**	*E. coli* ATCC 25922, *P. aeruginosa* ATCC 27853, and *Salmonella typhimurium* ATCC 13311	MICs of 45.06 to >223.72 μM	
				Epipolythiodioxopiperazines (**62**–**64**)	*Mycobacterium tuberculosis*	MICs, 0.55, 4.06 and 8.11 μM,	
28	*Chaetomium* sp. SYP-F7950	*Panax notoginseng*	Wenshan, Yunnan, China	Chaetocochin C (**63**), chetomin A (**65**), and chetomin (**62**)	*S. aureus*, *B. subtilis*, *Enterococcus faecium*	MIC values ranging from 0.12 to 19.3 μg/mL	[46]
29	*Chaetomium* sp. HQ-1,	*Astragalus chinensis*	Tai’an, Shandong Province, China	Differanisole A (**66**)	*L. monocytogenes S. aureus* and MRSA,	MIC, 16, 128, 128 μg/mL	[47]
				2,6-Dichloro-4-propylphenol (**67**), 4,5-dimethylresorcinol (**68**)	*L. monocytogenes*	MICs of 64 and 32 μg/mL,	
30	*Chaetomium nigricolor* F5,	*Mahonia fortune*	Qingdao, People’s Republic of China	Chamiside A (**69**)	*S. aureus*	MIC of 25 μg/mL	[48]
31	*C. globosum*	*Salvia miltiorrhiza*	Shenyang, Liaoning province, China	Equisetin (**70**)	Multidrug-resistant *E. faecalis*, *E. faecium*, *S. aureus*, and *S. epidermidis*	MIC values of 3.13, 6.25, 3.13, and 6.25 μg/mL	[49]
32	*Chaetomium* sp. Eef-10,	*Eucalyptus exserta*	Guangdong Province, China	Mollicellins H (**71**)	*S. aureus* ATCC29213, *S. aureus* N50, MRSA,	IC_50_, 5.14, and 6.21 μg/mL	[50]
				Mollicellin O (**72**)	*S. aureus* ATCC29213 and *S. aureus* N50	IC_50_, 79.44 and 76.35 μg/mL	
				Mollicellin I (**73**)		IC_50_, 70.14 and 63.15 μg/mL	
33	*Chaetomium* sp. M336	*Huperzia serrata*	Xichou County, Yunnan Province, China	6-Formamidochetomin (**74**)	*E. coli*, *S. aureus*, *S. typhimurium* ATCC 6539 and *E. faecalis*	MIC, 0.78 μg/mL	[51]
34	*Chaetomium globosum*	*Nymphaea nouchali*	Udugampola in the Gampaha District, Sri Lanka	Chaetoglobosin A (**75**)	*B. subtilis*, *S. aureus*, and MRSA	MIC, 16, 32 and 32 μg/mL	[52]
				Chaetoglobosin B (**76**)		>64 μg/mL	
	***Talaromyces***						
35	*Talaromyces pinophilus* XL-1193	*Salvia miltiorrhiza*	Shenyang, Liaoning province, China	Pinophol A (**77**)	*Bacterium paratyphosum* B	MIC, 50μg/mL	[53]
36	*Talaromyces purpureogenus* XL-25	*Panax notoginseng*	Shijiazhuang, Hebei Province, China	Talaroconvolutin A (**78**)	*B. subtilis* *Micrococcus lysodeikticus*, *Vibrio parahaemolyticus*	MIC value of 1.56 μM	[54]
				Talaroconvolutin B (**79**)		MIC = 0.73 and 0.18 μM	
37	*Talaromyces purpureogenus*	*Panax notoginseng*		(1*S*,5*S*,7*S*,10*S*)-dihydroxyconfertifolin (**80**)	*E. coli*	MIC, 25 μM	[55]
38	*Talaromyces funiculosus* -Salicorn 58.			Talafun (**81**)	*E. coli*, *S. aureus*	MIC, 18 and 93 μM	[56]
				N-(2′-hydroxy-3′-octadecenoyl)-9-methyl-4,8-sphingadienin (**82**)	*Mycobacterium smegmatis*, *S. aureus*, *Micrococcus tetragenus*, and *E. coli*	MIC, 85, 90, 24, and 68, 93 μM	
				Chrodrimanin A (**83**)	*S. aureus*, *M. tetragenus*, *Mycobacterium phlei*, and *E. coli*	MIC, 67, 28, 47, and 26 μM	
				Chrodrimanin B (**84**)	*E.coli*	MIC, 43 μM.	
39	*Talaromyces* sp. LGT-2	*Tripterygium wilfordii*.		Alkaloids **85**–**90**	*E. coli*, *P. aeruginosa*, *S. aureus*, *Bnfillus licheniformis*, and *Streptococcus pneumoniae*	MICs in the range of 0.125 to 1.0 50 μg/mL	[57]
40	*Rhytidhysteron* sp. BZM-9	*Leptospermum brachyandrum*		Euphorbol (**91**)	MRSA	MIC, 62.5 ug/mL	[58]
41	*Stagonosporopsis oculihominis*	*Dendrobium huoshanense.*		Stagonosporopsin C (**92**)	*Staphylococcus aureus* subsp. *aureus* ATCC29213	MIC_50_, 41.3 μM	[59]
42	*Eutypella scoparia* SCBG-8.	*Leptospermum brachyandrum*	SCBG, Chinese Academy of Sciences, China	Eutyscoparols H (**93**), I (**94**), tetrahydroauroglaucin (**95**), flavoglaucin (**96**)	*Staphylococcus aureus* and MRSA	MICs in the range of 1.25 to 6.25 μg/mL	[60]
43	*Eutypella scoparia* SCBG-8	*Leptospermum brachyandrum*	SCBG, Chinese Academy of Sciences, Guangzhou 510650, China	Eutyscoparin G (**97**)	*S. aureus* and MRSA	MIC values of 6.3 μg/mL	[61]
44	*Sarocladium oryzae* DX-THL3,	*Oryza rufipogon* Griff.		Sarocladilactone A (**98**), sarocladilactone B (**99**), helvolic acid (**100**), helvolinic acid (**101**), 6- desacetoxy-helvolic acid (**102**), 1,2-dihydrohelvolic acid (**103**)	*S. aureus*	MIC values of 64, 4, 8, 1, 4 and 16 μg/mL	[62]
				Compound **101**	*B. subtilis*	MIC, 64 μg/mL	
				Compounds **99**, **101**, **103**	*E. coli*	MIC 64 μg/mL each	
45	*Paraphaeosphaeria sporulosa*	*Fragaria x ananassa*	Caserta province, Southern Italy	Cyclo(L-Pro-L-Phe) (**104**)	*Salmonella* strains, S1 and S2	MIC 71.3 and 78.6 μg/mL	[63]
46	*Aplosporella javeedii*	*Orychophragmus violaceus*	Beijing, China	Terpestacin (**105**), fusaproliferin (**106**), mutolide (**108**)	*M. tuberculosis* H37Rv	MICs of 100 μM	[64]
				6,7,9,10-Tetrahydromutolide (**107**)	*S. aureus*,	MICs of 100 μM	
47	*Pleosporales* sp. Sigrf05	roots of *Siraitia grosvenorii*	Guangxi Province of China	Pleospyrone E (**109**)	*B. subtilis*, *Agrobacterium tumefaciens*, *Ralstonia solanacearum*, and *Xanthomonas vesicatoria*	MIC 100.0µM each	[65]
48	*Aplosporella javeedii*	*Orychophragmus violaceus*	Beijing, China	Aplojaveediin A (**110**)	*Staphylococcus aureus* strain ATCC 29213, *S. aureus* strain ATCC 700699 and *Bacillus subtilis* (ATCC 169)	MICs 50, 50 and 25 μM,	[66]
				Aplojaveediin F (**111**)	*S. aureus* ATCC 29213 and ATCC 700699	MICs of 25 and 50 μM	
49	*Paecilomyces variotii*	*Lawsonia Alba*	University of Karachi, Pakistan	Lawsozaheer (**112**)	*S. aureus* (NCTC 6571)	84.26% inhibition at 150 μg/mL	[67]
50	*Preussia isomera* OSMAC strategy	*Panax notoginseng*	Wenshan, Yunnan Province, China	Setosol (**113**)	Multidrug-resistant *E. faecium*, methicinllin-resistant *S. aureus* and multidrug-resistant *E. faecalis*	MIC 25 μg/mL	[68]
	*Preussia isomera*. XL-1326,	*Panax notoginseng*		(+)- and (−)-Preuisolactone A (**114**, **115**)	*Micrococcus luteus* and *B. megaterium*	MIC, 10.2 and 163.4 μM	[69]
51	*Neurospora udagawae*	*Quercus macranthera*	Kaleybar region in northwestern Iran	Udagawanones A (**116**)	*S. aureus*	MIC, 66 μg/mL	[70]
52	*Xylomelasma* sp. Samif07	Salvia miltiorrhiza Bunge		2,6-Dimethyl-5-methoxy-7-hydroxychromone (**117**), 6-hydroxymethyleugenin (**118**), 6-methoxymethyleugenin (**119**), isoeugenitol (**120**), diaporthin (**29**), 8-hydroxy-6-methoxy-3-methylisocoumarin (**121**)	*Bacillus subtilis*, *Staphylococcus haemolyticus*, *A. tumefaciens*, *Erwinia carotovora*, and *Xanthomonas vesicatoria*	MIC values at the range of 25 ~ 100 μg/mL	[71]
				2,6-Dimethyl-5-methoxy-7-hydroxychromone (**117**), diaporthin (**29**)	*B. subtilis*, *E. carotovora*	MIC, 50 and 100 μg/mL	
				6-Hydroxymethyleugenin (**118**), 6-methoxymethyleugenin (**119**), isoeugenitol (**120**), diaporthin (**29**)	*S. haemolyticus* and *E. carotovora*	MIC, 75 μg/mL each	
				8-Hydroxy-6-methoxy-3-methylisocoumarin (**121**)	*B. subtilis*, *A. tumefaciens*, and *X. vesicatoria*,	MICs 25, 75, and 25 μg/mL,	
53	*Amphirosellinia nigrospora*JS-1675	*Pteris cretica*		(4*S*,5*S*,6*S*)-5,6-epoxy-4-hydroxy-3-methoxy-5-methylcyclohex-2-en-1-one (**122**)	*Acidovorax avenae* subsp. *cattlyae*, *Agrobacterium konjaci*, *A. tumefaciens*, *Burkholderia glumae*, *Clavibacter michiganensis* subsp. *michiganensis*, *Pectobacterium carotovorum* subsp. *carotovorum*, *Pectobacterium chrysanthemi*, *Ralstonia solanacearum*, *Xanthomonas arboricola* pv. *pruni*, *Xanthomonas axonopodis* pv. Citri, *Xanthomonas euvesicatoria*, *Xanthomonas oryzae* pv. *oryzae*	MICs ranging between 31.2 and 500 µg/ml	[72]
54	*Emericella* sp. XL029	*Panax notoginseng*		5-(Undeca-3′,5′,7′-trien-1′-yl)furan-2-ol (**123**) and 5-(undeca-3′,5′,7′-trien-1′-yl)furan-2-carbonate (**124**)	*B. subtilis*, *B. cereus*, *S. aureus*, *B. paratyphosum* B, *S. typhi*, *P. aeruginosa*, *E. coli*, and *E. aerogenes*	MIC values ranging from 6.3 to 50 μg/mL	[73]
56	*Emericella* sp. XL029	*Panax notoginseng*	Shijiazhuang, Hebei Province, China	14-Hydroxytajixanthone (**125**), 14- hydroxytajixanthone hydrate (**126**), 14- hydroxy-15-chlorotajixanthone hydrate (**127**), 14-methoxytajixanthone-25-acetate (**130**), questin (**132**), and carnemycin B (**133**)	*M. luteus*, *S. aureus*, *B. megaterium*, *B. anthracis*, and *B. paratyphosum* B	MIC, in the range of of 12.5 and 25μg/mL	[74]
				Epitajixanthone hydrate (**128**)	*M. luteus*, *S. aureus*, *B. megaterium*, and *B. paratyphosum* B	MIC 25 μg/mL	
				Tajixanthone hydrate (**129**), 15-chlorotajixanthone hydrate (**131**)	*S. aureus*, *B. megaterium*, and *B. paratyphosum* B	MICs 25 and 12.5 μg/mL,	
				14-Hydroxytajixanthone (**125**) Epitajixanthone hydrate (**128**), carnemycin B (**133**)	drug resistant *S. aureus*	MIC 50 μg/mL	
				Compounds **125**–**133**	*P. aeruginosa*, *E. coli*, and *E. aerogenes*	MIC 50 μg/mL	
57	*Byssochlamys spectabilis*	*Edgeworthia chrysantha*	Hangzhou Bay, Hangzhou, Zhejiang Province, China	Bysspectin C (**134**)	*E. coli* ATCC 25922 and *S. aureus* ATCC 25923	MIC, 32 and 64 µg/mL	[75]
58	*Poculum pseudosydowianum* (TNS-F-57853),	*Quercus crispula* var. *crispula*	Yoshiwa, Hatsukaichi, Hiroshima prefecture, Japan	Sydowianumols A (**135**), and B (**136**)	MRSA	MIC90 values of 12.5 μg/mL	[76]
59	*Lachnum palmae* exposure to a HDAC inhibitor SAHA	*Przewalskia tangutica*	Linzhou Country of the Tibet Autonomous Region, China	Palmaerones A-B, E-G (**137**, **138**, **140**, **141**, **142**)	*B. subtilis*	MICs, 35, 30, 10, 50, and 55 μg/mL	[77]
				Palmaerones A-C, E (**137**, **138**, **139**, **140**)	*S. aureus*	MICs 65, 55, 60, and 55, μg/mL	
60	*Nemania serpens*	*Vitis vinifera*	Canada’s Niagara region	Nemanifuranone A (**143**)	*E. coli*	MIC 200 μg/mL	[78]
					*S. aureus*, *B. subtilis* and *M. luteus*	>75% inhibition at a concentration of 100–200 μg/mL	
				Triterpenoid **144**	*S. cerevisiae*	(>25% inhibition) against at 200 μg/mL	
					*M. luteus*	(>75% inhibition) of at a concentration of 100 μg/mL	
61	*Paraconiothyrium variabile*	*Cephalotaxus harringtonia*		Variabilone (**145**)	*B. subtilis*	IC_50_ of 2.13 μg/mL after 24 h (0.36 μg/mL for kanamycin)	[79]
62	*Pyronema* sp. (A2-1 & D1-2)	*Taxus mairei*	Shennongjia National Nature Reserve, Hubei province, China.	Methyl 2-{(*E*)-2-[4-(formyloxy)phenyl] ethenyl}-4-methyl-3-oxopentanoate (**146**), (3*R*,6*R*)-4-methyl-6-(1-methylethyl)-3-phenylmethyl-perhydro-1,4-oxazine-2,5-dione (**147**), (3*R*,6*R*)-N-methyl-N-(1-hydroxy-2-methylpropyl)-phenylalanine (**148**), siccanol (**149**), fusaproliferin (**106**), and sambutoxin (**150**)	*Mycobacterium marinum* ATCCBAA-535,	IC_50_ of 64, 59, 57, 84, 43 and 32 μM, (positive control rifampin IC_50_ of 2.1 μM)	[80]
63	*Pulvinula* sp. 11120	*Cupressus arizonica*	Tucson, AZ, USA	Pulvinulin A (**151**), graminin C (**152**), cis-gregatin B (**153**), and graminin B (**154**)	*E. coli*	12, 18, 16 and14 mm zone of inhibition at 100 μg/mL	[81]
64	*Stelliosphaera formicum*	*Duroia hirsuta*	Yasuni’ National Park off the Napo River in Ecuador	Stelliosphaerols A (**155**) and B (**156**)	*S. aureus*	MIC values of 250 μg/mL	[82]
65	Unidentified Ascomycete	*Melilotus dentatus*		cis-4-Acetoxyoxymellein (**157**)	*E. coli* and *B. megaterium*	Zone of inhibition of 10 and 10 mm (Partial inhibition) at a concentration of 0.05 mg	[83]
				8-Deoxy-6-hydroxy-*cis*-4-acetoxyoxymellein (**158**)	*E. coli* and *B. megaterium*	Zone of inhibition of 9 and 9 mm (Partial inhibition) at a concentration of 0.05 mg	
	**Anamorphic Ascomycetes**						
	***Aspergillus***						
66	*Aspergillus* sp. FT1307	*Heliotropium* sp.		Aspochalasin P (**159**), alatinone (**160**), β-11-methoxy curvularine (**161**), 12-keto-10,11-dehydrocurvularine (**162**)	*S. aureus* ATCC12600, *B. subtilis* ATCC6633 and MRSA ATCC43300	MIC in the range of 40 to 80 μg/mL	[84]
67	*Aspergillus cristatus*	*Pinellia ternata*		Aspergillone A (**163**)	*B. subtilis* and *S. aureus*	MIC_50_, 8.5 and 32.2 μg/mL	[85]
68	*Aspergillus versicolor* strain Eich.5.2.2	*Eichhornia crassipes*	El-Kanater El-Khayriah in Egypt	22*S*-Aniduquinolone A (**164**), 22R-aniduquinolone A (**165**)	*S. aureus* (ATCC700699)	MIC, 0.4 μg/mL	[86]
69	*Aspergillus versicolor*	roots of *Pulicaria crispa*	Saudi Arabia	Aspergillether B (**166**)	*S. aureus*, *B. cereus*, and *E. coli*	MICs, 4.3, 3.7, and 3.9 μg/mL	[87]
70	*Aspergillus ochraceus* SX-C7 *eus* SX-C7	*Setaginella stauntoniana*		3-O-β-D-Glucopyranosyl stigmasta-5(6),24(28)-diene (**167**)	*Bacillus subtilis*	MIC, 2 μg/mL	[88]
71	*Aspergillus amstelodami* (MK215708)	Ammi majus	Egypt	Dihydroauroglaucin (**168**)	*E. coli*, *Streptococcus mutans*, *S. aureus*	MIC, 1.95, 1.95 and 3.9 μg/mL	[89]
					*S. aureus*, *E. coli*, *Streptococcus mutans*, *P. aeruginosa*	Minimum biofilm inhibitory concentration (MBIC) = 7.81, 7.81, 15.63 and 31.25 μg/mL	
72	*Aspergillus micronesiensis*	*Phyllanthus glaucus*	LuShan Mountain, Jiangxi Province, China	Cyschalasins A (**169**) and B (**170**)	MRSA	MIC_50_, 17.5 and 10.6 μg/mL: MIC90, 28.4 and 14.7 μg/mL	[90]
73	*A. niger*	*Acanthus montanus*	Kala Mountain neighborhood of Yaoundé, Africa	Methylsulochrin (**171**)	*S. aureus*, *Enterobacter cloacae* and *Enterobacter aerogenes*	MIC, 15.6, 7.8 and 7.8 μg/mL	[91]
74	*Aspergillus tubingensis*	stem of *Decaisnea insignis*	Qinling Mountain, Shaanxi Province, China	3-(5-Oxo-2,5-dihydrofuran-3-yl) propanoic acid (**172**)	*Streptococcus lactis*	MIC value of 32 μg/mL	[92]
75	*Aspergillus flavipes* Y-62	*Suaeda glauca*	Zhoushan coast, Zhejiang province, East China	Methyl 2-(4-hydroxybenzyl)-1,7-dihydroxy-6-(3-methylbut-2-enyl)-1*H*-indene-1-carboxylate (**173**)	MRSA	MIC, 128 μg/mL	[93]
					*K. pneumoniae* and *P. aeruginosa*	MIC, of 32 μg/mL each	
76	*Aspergillus* sp.	Rhizome of *Zingiber cassumunar*		4-Amino-1-(1,3-dihydroxy-1-(4-nitrophenyl)propan-2-yl)-1*H*-1,2,3-triazole-5(4H)one (**174**)	*Xanthomonas oryzae*, *Bacillus subtilis* and *E. coli*	Zone of inhibition 37, 30 and 27 mm	[5]
				3,6-Dibenzyl-3,6-dimethylpiperazine-2,5-dione (**175**)	*E. coli* and *X. oryzae*	Zone of inhibition 21 and 16 mm.	
77	*Aspergillus fumigatus*	*Edgeworthia chrysantha*	Hangzhou Bay (Hangzhou, China)	Pseurotin A (**176**), spirotryprostatin A (**177**)	*S. aureus*	MIC of 0.39 µg/mL each	[94]
				Spirotryprostatin A (**177**)	*E. coli*	MIC, 0.39 µg/mL	
78	*Aspergillus* sp.,	*Astragalus membranaceus*		Fumiquinazoline J (**178**), fumiquinazoline C (**180**), fumiquinazoline H (**181**), fumiquinazoline D (**182**)	*B. subtilis*, *S. aureus*, *E. coli* and *P. aeruginosa*	MICs in the range of 0.5–8 μg/mL	[95]
				Fumiquinazoline I (**179**), fumiquinazoline B (**183**)		MICs in the range of 4–16 μg/mL	
79	*Aspergillus fumigatiaffnis*	Tribulus terestris		(−)-Palitantin (**184**)	*E. faecalis* UW 2689 and *Streptococcus pneumoniae*	MIC, 64μg/mL	[96]
80	*Aspergillus* sp. TJ23	*Hypericum perforatum* (St John’ Wort)	Shennongjia areas of Hubei Province, China	Aspermerodione (**185**)	MRSA	MIC, 32 μg/mL/potential inhibitor of PBP2a	[97]
				Andiconin C (**186**)		marginal antimicrobial activity (>100μg/mL)	
81	*Aspergillus* sp. YXf3	*Ginkgo biloba*		Prenylterphenyllin D (**187**), prenylterphenyllin E (**188**), 2′-O-Methylprenylterphenyllin (**189**), prenylterphenyllin (**190**)	*X. oryzae* pv. *oryzicola* Swings and *E. amylovora*	MIC, 20 μg/mL each	[98]
				Prenylterphenyllin B (**191**)	*E. amylovora*	MIC, 10 μg/mL	
82	*Aspergillus* sp.	*Pinellia ternata*	Nanjing, Jiangsu Province, China	Aspergillussanone D (**192**)	*P. aeruginosa*, and *S. aureus*	MIC_50_, 38.47 and 29.91 μg/mL	[99]
				Aspergillussanone E (**193**)	*E. coli*	MIC_50_, 7.83 μg/mL	
				Aspergillussanone F (**194**)	*P. aeruginosa*, and *S. aureus*	MIC_50_, 26.56, 3.93 and 16.48 μg/mL	
				Aspergillussanone G (**195**)	*P. aeruginosa*, and *S. aureus*,	MIC_50_, 24.46 and 34.66 μg/mL	
				Aspergillussanone H (**196**)	*P. aeruginosa*, and *E. coli*,	MIC_50_, 8.59 and 5.87 μg/mL	
				Aspergillussanone I (**197**)	*P. aeruginosa*,	MIC_50_, 12.0 μg/mL	
				Aspergillussanone J (**198**)	*P. aeruginosa*, *E. coli* and *S. aureus*	MIC_50_, 28.50, 5.34 and 29.87 μg/mL	
				Aspergillussanone K (**199**)	*P. aeruginosa*, and *S. aureus*,	MIC_50_, 6.55 and 21.02 μg/mL	
				Aspergillussanone L (**200**)	*P. aeruginosa*, *S. aureus*, and *B. subtilis*	MIC_50_, 1.87, 2.77, and 4.80 μg/mL,	
				Compound **201**	*P. aeruginosa*, and *E. coli*,	MIC_50_, 19.07 and 1.88 μg/mL	
83	*Aspergillus terreus* JAS-2	*Achyranthus aspera*	Varanasi, India	Terrein (**202**)	*E. faecalis*	IC_50_, 20 μg/mL	[100]
					*S. aureus* and *Aeromonas hydrophila*	20 μg/mL	
84	*Aspergillus terreus*	roots of *Carthamus lanatus*	Al-Azhar University campus in Cairo, Egypt	(22*E*,24*R*)-Stigmasta-5,7,22-trien-3-*β*-ol (**203**)	MRSA	IC_50_, 2.29 µM	[101]
85	*Aspergillus flavus*	*Cephalotaxus fortunei*	Taibai Mountains, Shaanxi Province, China	5-Hydroxymethylfuran-3-carboxylic acid (**204**), 5-acetoxymethylfuran-3-carboxylic acid (**205**)	*S. aureus*	MIC, 31.3 and 15.6 μg/mL	[102]
86	*Aspergillus allahabadii* BCC45335	root of *Cinnamomum subavenium*	Khao Yai National Park, Nakhon Ratchasima Province, Thailand	Allahabadolactone B (**206**), (22*E*)-5α,8α-epidioxyergosta-6,22-dien-3β-ol (**207**)	*B. cereus*	IC_50_, 12.50 and 3.13 µg/mL.	[103]
87	*Aspergillus tubingensis*	*Lycium ruthenicum*		6-Isovaleryl-4-methoxypyran-2-one (**208**), asperpyrone A (**210**), campyrone A (**211**)	*E. coli*, *Pseudomonas aeruginosa*, *Streptococcus lactis* and *S. aureus*	MIC values ranging from 62.5 to 500 μg/mL	[104]
				Rubrofusarin B (**209**)	*E. coli*	MIC, 1.95 μg/mL	
88	*Aspergillus tamarii* FR02	roots of *Ficus carica*	Qinling Mountain in China’s Shaanxi province	Malformin E (**212**)	*B. subtilis*, *S. aureus*, *P. aeruginosa*, and *E. coli*	MIC, 0.91, 0.45, 1.82, and 0.91 μM	[105]
89	*Aspergillus terreus*	Roots of *Carthamus lanatus*	Al-Azhar University campus, Egypt	(22*E*,24*R*)-Stigmasta-5,7,22-trien-3-*β*-ol (**203**)	MRSA	IC_50_, 0.96μg/mL	[106]
				Aspernolide F (**213**)		IC_50_ 6.39μg/mL	
90	*Aspergillus* sp. (SbD5)	Leaves of *Andrographis paniculata*	Indralaya, Ogan Ilir, South Sumatra.	1-(3,8-Dihydroxy-4,6,6-trimethyl-6*H*-benzochromen-2-yloxy)propane-2-one (**214**), 5-hydroxy-4-(hydroxymethyl)-2*H*-pyran-2-one (**215**), (5-hydroxy-2-oxo-2*H*-pyran-4-yl)methyl acetate (**216**)	*S. aureus*, *E. coli*, *S. dysenteriae* and *Salmonella typhi*	Zone of inhibition diameters ranging from 8.1 to 12.1 mm at a concentration 500 μg/mL.	[107]
91	*Aspergillus* sp. IFB-YXS	*Ginkgo biloba*		Xanthoascin (**217**)	*X. oryzae* pv. *oryzicola*, *Swings*, *E.amylovora*, *P. syringae* pv. *Lachrymans* and *C. michiganense* subsp. *sepedonicus*	MICs, 20, 10, 5.0 and 0.31 µg/mL	[108]
				Prenylterphenyllin B (**218**)	*X. oryzae* pv.*oryzicola Swings*, *E.amylovora*, *P. syringae* pv. *Lachrymans*,	MICs of 20 µg/mL each	
				Prenylcandidusin (**219**)	*X. oryzae* pv.*oryzae Swings X. oryzae* pv. *oryzicola* Swings	MIC values of 10 and 20 µg/mL	
	***Penicillium***						
92	*Penicillium ochrochloron* SWUKD4.1850	*Kadsura angustifolia*		4-O-Desmethylaigialomycin B (**220**), penochrochlactones C (**221**) and D (**222**)	*Staphylococcus aureus*, *Bacillus subtilis*, *Escherichia coli*, and *Pseudomonas aeruginosa*	MIC values between 9.7 and 32.0 μg/mL	[109]
93	*Penicillium brefeldianum*	*Syzygium zeylanicum*		*p*-Hydroxybenzaldehyde (**223**),	*S. typhi*, *E. coli*, and *B. subtilis*	MIC values of 64 g/mL	[110]
94	*Penicillium vulpinum* GDGJ-91	*Sophorae tonkinensis*	Baise, Guangxi Province, China	10-Demethylated andrastone A (**224**), andrastin A (**227**)	*Bacillus megaterium*	MIC value of 6.25 μg/mL	[111]
				Citreohybridone E (**225**), citreohybridonol (**226**), citreohybridone B (**228**)	*B. megaterium*	MIC values of 25, 12.5 and 25 μg/mL	
				Citreohybridonol (**226**)	*B. paratyphosus* B, *E. coli* and *S. aureus*	MIC, 6.25, 25 and 25 μg/mL	
				10-Demethylated andrastone A (**224**), citreohybridone E (**225**), andrastin A (**227**), andrastin B (**228**)	*B. paratyphosus* B	MIC, 12.5 or 25 μg/mL.	
95	*Penicillium nothofagi* P-6,	*Abies beshanzuensis*	Baishanzu Mountain in Lishui, Zhejiang Province of China	Chromenopyridin A (**229**), viridicatol (**230**)	*S. aureus* ATCC29213	MIC, 62.5 and 15.6 μg/mL	[112]
96	*Penicillium restrictum* (strain G85)	*Silybum marianum*	Horizon Herbs, LLC (Williams, OR, USA).	ω-Hydroxyemodin (**231**)	Clinical isolates of MRSA	Quorum-sensing inhibition in both in vitro and in vivo models	[113]
97	*Penicillium vulpinum*	*S. tonkinensis*	Baise, Guangxi Province, China	(−)-3-Carboxypropyl-7-hydroxyphthalimide (**232**)	*Shigella dysenteriae* and *Enterobacter areogenes*	MIC, 12.5 μg/mL each	[114]
					*B. subtilis*	MIC, 25 μg/mL	
					*B. megaterium* and *Micrococcus lysodeikticus*	MIC, 50 μg/mL	
				(−)-3-Carboxypropyl-7-hydroxyphthalide methyl ester (**233**)	E. areogenes	MIC, 12.5 μg/mL	
					*B. subtilis*, *B. megaterium* and *M. lysodeikticus*	MIC, 100 μg/mL.	
98	*Penicillium sumatrense* GZWMJZ-313	Leaf of *Garcinia multiflora*	Libo, Guizhou Province of China	Citridone E (**234**), (–)-dehydrocurvularin (**235**)	*S. aureus*, *P. aeruginosa*, *Clostridium perfringens*, and *E. coli*	MIC values ranging from 32 to 64 μg/mL	[115]
99	*Penicillium ochrochloronthe*	Roots of Taxus media	Qingfeng Mountain, Chongqing, China	3,4,6-Trisubstituted α-pyrone derivatives, namely 6-(2′*R*-hydroxy-3′*E*,5′*E*-diene-1′-heptyl)-4-hydroxy-3-methyl-2*H*-pyran-2-one (**236**), 6-(2′S-hydroxy-5′*E*-ene-1′-heptyl)-4-hydroxy-3-methyl-2*H*-pyran2-one (**237**), 6-(2′S-hydroxy-1′-heptyl)-4 -hydroxy-3-methyl-2*H*-pyran-2-one (**238**), trichodermic acid (**239**)	*B. subtilis*, *Micrococcus luteus*, *S. aureus*, *B. megaterium*, *Salmonella enterica*, *Proteusbacillm vulgaris*, *Salmonella typhi*, *P. aeruginosa*, *E. coli* and *Enterobacter aerogenes*	MIC values ranging from 25 to 50 μg/mL	[116]
100	*Penicillium janthinellum* SYPF 7899	*Panax notoginseng*	Wenshan region, Yunnan province, China	Brasiliamide J-a (**240**), brasiliamide J-b (**241**)	*B. subtilis* and *S. aureus*	MIC, 15 and 18 μg/mL,	[117]
				Peniciolidone (**242**), austin (**243**)	*B. subtilis*	MIC, 35 and 50 μg/mL	
					*S. aureus*	MIC 39, and 60 μg/mL	
101	*Penicillium cataractum* SYPF 7131	*Ginkgo biloba*		Penicimenolidyu A (**244**), penicimenolidyu B (**245**) and rasfonin (**246**)	*S. aureus*	MIC 65, 59 and 10 μg/mL	[118]
102	*Penicillium* sp.,	Tubers of *Pinellia ternata*	suburb of Nanjing, Jiangsu, China.	3′-Methoxycitreovirone (**247**), citreovirone (**249**)	*E. coli* and *S. aureus*	MIC = 62.6 and 76.6 μg/mL	[119]
				Helvolic acid (**100**)	*S. aureus*, *P. aeruginosa*, *B. subtilis* and *E. coli*	MIC = 5.8, 4.6, 42.2 and 75.0 μg/mL	
				*cis-*bis-(Methylthio)-silvatin (**248**), trypacidin A (**250**)	*S. aureus*	MIC values of 43.4 and 76.0 μg/mL	
				Trypacidin A (**250**)	*B. subtilis*	MIC = 54.1 μg/mL	
103	*Penicillium* sp. R22	*Nerium indicum*	Qinling Mountain, Shaanxi Province, China	Viridicatol (**251**)	*S. aureus*	MIC value of 15.6 μg/mL	[120]
104	*Penicillium* sp. (NO. 24)	*Tapiscia sinensis*	Shennongjia National Forest Park China	Penicitroamide (**252**)	*Erwinia carotovora* subsp. Carotovora	MIC_50_ at 45 μg/mL	[121]
105	*Penicillium* sp. CAM64	Leaves of *Garcinia nobilis*	Mount Etinde, Southwest region Cameroon	Penialidin A (**253**)	*Vibrio cholerae* SG24 (1), *V. cholerae* CO6, *V. cholerae* NB2, *V. cholerae* PC2, *S. flexneri* SDINT,	MIC, 8–32 μg/mL	[122]
				Penialidin B (**254**)		MIC, 4–32 μg/mL	
				Penialidin C (**255**)		MIC, 0.50, 16, 8, 0.50 and 8 μg/mL	
				Citromycetin (**256**), brefelfin A (**258**)		MIC, 64–128 μg/mL	
				*p*-Hydroxyphenylglyoxalaldoxime (**257**)		MIC, 32–64 μg/mL	
106	*Purpureocillium lilacinum*	roots of *Rauvolfia macrophylla*	Mount Kalla in the Center Region of Cameroon	Purpureone (**259**)	*B. cereus*, *L. monocytogenes*, *E. coli* ATCC 8739, *K. pneumoniae* ATCC 1296, *P. stuartii* ATCC 29916, *P. aeruginosa* ATCC PA01	Zone of inhibition of 10.6, 12.3, 13.0, 8.7, 12.3, and 10.0, mm against (10 μL/6 mm Filter paper disks).	[123]
	***Fusarium***						
	*Neocosmospora* sp. MFLUCC 17-0253	*Rhizophora apiculate.*		Mixture of 2-methoxy-6-methyl-7-acetonyl-8-hydroxy-1,4-naphthalenedione (**260**), and 5,8-dihydroxy-7-acetonyl-1,4-naphthalenedione (**261**)	*Acidovorax citrulli*	MIC value of 0.0075 mg/mL	[124]
				Anhydrojavanicin (**262**)		0.004 mg/mL	
				Fusarnaphthoquinone (**263**)		0.025 mg/mL	
107	*Fusarium* sp.	*Mentha longifolia*	Al Madinah Al Munawwarah, Saudi Arabia.	Fusaribenzamide A (**264**)	*S. aureus* and *E. coli*	MICs, 62.8 and 56.4 μg/disc	[125]
108	*F. proliferatum* AF-04	Green Chinese onion		5-O-Methylsolaniol (**270**), 5-O-methyljavanicin (**271**), methyl ether fusarubin (**272**), anhydrojavanicin (**273**)	*B. megaterium*	MICs 25 μg/mL each.	[126]
				5-O-Methylsolaniol (**270**), 5-O-methyljavanicin (**271**), methyl ether fusarubin (**272**)	*B. subtilis*	MICs, 50 μg/mL each.	
				Indol-3-acetic acid (**265**), beauvericin (**267**), epicyclonerodiol oxide (**269**)	*B. megaterium*	MICs 50 μg/mL each	
				Cyclonerodiol (**268**)	*B. megaterium*	MIC 12.50 μg/mL.	
				*epi*-Cyclonerodiol oxide (**269**), methyl ether fusarubin (**272**)	*E. coli*	MIC 50 μg/mL	
				5-O-Methylsolaniol (**270**), 5-O-methyljavanicin (**271**), anhydrojavanicin (**273**)	*E. coli*	MIC 25 μg/mL	
				*epi*-Cyclonerodiol oxide (**269**), 1,4-naphthoquinones, 5-O-methylsolaniol (**270**), 5-O-methyljavanicin (**271**), methyl ether fusarubin (**272**)	*Clostridium perfringens*	MICs 50, 50, 12.5 and 50 μg/mL	
				Beauvericin (**267**), fusaproliferin (**106**), 5-O-methylsolaniol (**270**), 5-O-methyljavanicin (**271**), methyl ether fusarubin (**272**), anhydrojavanicin (**273**)	MRSA	MIC value of 50, 50, 12.5, 12.5, 12.5, and 25 μg/mL respectively.	
				5-O-Methyljavanicin (**271**), methyl ether fusarubin (**272**), anhydrojavanicin (**273**)	RN4220	MIC value of 50 μg/mL each.	
				Methyl ether fusarubin (**272**), anhydrojavanicin (**273**)	NewmanWT	MIC value of 50 μg/mL each.	
				Bassiatin (**266**)	NewmanWT	MIC, 50 μg/mL	
109	*Fusarium* sp. TP-G1	*Dendrobium officinable*	Chongqing Academy of Chinese Materia Medica in China	Trichosetin (**274**), beauvericin (**267**), beauvericin A (**275**), enniatin H (**277**), enniatin I (**278**), enniatin MK1688 (**279**)	*S. aureus* and MRSA	IC_50_ values in the range of 2–32 μg/mL	[127]
				Enniatin B (**276**)	*S. aureus* and MRSA	IC_50_, 128 μg/mL each	
				Fusaric acid (**280**), dehydrofusaric acid (**281**)	*Acinetobacter baumannii*	MIC, 64 and 128 μg/mL	
	*Fusarium* sp. YD-2	*Santalum album*	Dongguan, Guangdong Province, China	Fusariumin A (**282**)	*S. aureus* and *P. aeruginosa*	MIC, 6.3 μg/mL	[128]
				Asperterpenoid A (**283**)	*Salmonella enteritidis* and *Micrococcus luteus*	MIC, 25.2 and 6.3 μg/mL	
				Agathic acid (**284**)	*B. cereus* and *M. luteus*	MIC, 12.5 and 25.4 μg/mL	
110	*Fusarium chlamydosporium*	Leaves of *Anvillea garcinii*	Al-Azhar University campus, Egypt	Fusarithioamide B (**285**)	*E. coli*, *B. cereus*, and *S. aureus*	MIC value of 3.7, 2.5 and 3.1 µg/mL	[129]
111	*Fusarium solani* A2	*Glycyrrhiza glabra*	Kashmir Himalayas of Jammu and Kashmir State, India	3,6,9-Trihydroxy-7-methoxy-4,4-dimethyl-3,4-dihydro-1*H*-benzo[g]-isochromene-5,10-dione (**286**), fusarubin (**287**), 3-O-methylfusarubin (**288**), javanicin (**289**)	*S. aureus* (MTCC 96), *K. pneumonia* (MTCC 109), *S. pyogenes* (MTCC 442), *B. subtilis* (MTCC 121), *B. cereus* (IIIM 25), *Micrococcus luteus* (MTCC 2470) and *E. coli* (MTCC 730)	MIC values in the range of <1 to 256 μg/mL.	[130]
				Fusarubin (**287**)	*Mycobacterium tuberculosis* strain H37Rv	MIC, 8 μg/mL,	
				3,6,9-Trihydroxy-7-methoxy-4,4-dimethyl-3,4-dihydro-1*H*-benzo[g]-isochromene-5,10-dione (**286**), 3-O-methylfusarubin (**288**), javanicin (**289**)		MIC values of 256, 64, 32 μg/mL	
112	*Fusarium chlamydosporium*	*Anvillea garcinii*	Al-Azhar University, Saudi Arabia	Fusarithioamide A (**290**)	*B. cereus*, *S. aureus*, and *E. coli*	MICs values of 3.1, 4.4, and 6.9 μg/mL	[131]
113	*Fusarium* sp.	*Rhoeo spathacea*	Pondok Cabe, Banten, Indonesia.	Javanicin (**289**)	*M. tuberculosis* and *M. phlei*	MIC 25 and 50 μg/mL	[132]
114	*Fusarium* sp.	*Ficus carica*	Qinling Mountain, Shaanxi Province, China	Helvolic acid Me ester (**291**)	*B. subtilis*, *S. aureus*, *E. coli* and *P. aeruginosa*	MIC, 6.25, 12.5, 6.25, and 3.13 μg/mL	[133]
				Helvolic acid (**100**)		MICs 6.25, 6.25, 6.25, and 3.13 μg/mL	
				hydrohelvolic acid (**292**)		MICs 6.25, 12.5, 6.25, and 3.13 μg/mL	
115	*Fusarium* sp.	*-*	-	Colletorin B (**293**), 4,5-dihydroascochlorin (294)	*B. megaterium*	5 and 10 mm zone of inhibition at 10 μg/mL concentration of	[134]
116	*Fusarium* sp.	*Opuntia dillenii*	South-Eastern arid zone of Sri Lanka	Equisetin (**295**)	*B. subtilis*	MIC, 8 μg/mL	[135]
					*S. aureus* and MRSA.	MIC, 16 μg/mL	
117	*Trichoderma harzianum*	*Zingiber officinale*	Banyumas, Central Java, Indonesia	Pretrichodermamide A (**296**)	*M. tuberculosis*	MIC, 25 μg/mL (50 μM)	[136]
118	*Trichoderma koningiopsis* YIM PH30002	*Panax notoginseng*		Koninginin W (**297**), koninginin D (**298**), 7-O- and koninginin A (**301**)	*B. subtilis*	MIC of 128 μg/mL.	[137]
				Koninginin W (**297**), 7-O-methylkoninginin D (299)	*S. typhimurium*	MIC, 64 and 128 μg/mL;	
				Koninginin W (**297**), koninginin (**300**)	*E. coli*	MIC of 128 μg/mL.	
119	*Trichoderma virens* QA-8	*Artemisia argyi*		Trichocarotins I–M (**302**–**306**), CAF-603 (**307**), 7*β*-hydroxy CAF-603 (**308**), trichocarotins E–H (**309**–**312**), and trichocarane A (**313**)	*E. coli* EMBLC-1,	MIC values ranging from 0.5 to 32 µg/mL MIC = 0.5 µg/mL	[138]
				7β-Hydroxy CAF-603 (**308**)	*Micrococcus luteus* QDIO-3		
120	*Trichoderma koningiopsis* QA-3	*Artemisia argyi.*		Trichodermaketone E (**314**), trichopyranone A (**316**), 3-hydroxyharziandione (**317**) and 10,11-dihydro-11-hydroxycyclonerodiol (**318**), harziandione (**321**)	*E. coli*	MIC values ranging from 0.5 to 64 μg/mL	[139]
				Trichopyranone A (**316**), 3-hydroxyharziandione (**317**), 10,11-dihydro-11-hydroxycyclonerodiol (**318**), cyclonerodiol (**319**), 6-(3-hydroxypent-1-en-1-yl)-2H-pyran-2-one (**320**), harziandione (**321**)	*M. luteus*	MIC values ranging from 1 to 16 μg/mL	
				Trichodermaketone E (**314**), 4-epi-7-O-methylkoninginin D (**315**), 3-hydroxyharziandione (**317**), 10,11-dihydro-11-hydroxycyclonerodiol (**318**), cyclonerodiol (**319**), 6-(3-hydroxypent-1-en-1-yl)-2H-pyran-2-one (**320**), harziandione (**321**)	*P. aeruginosa*	with MIC values ranging from 4 to 16 μg/mL	
				Trichodermaketone E (**314**), 10,11-dihydro-11-hydroxycyclonerodiol (**318**), cyclonerodiol (**319**), 6-(3-hydroxypent-1-en-1-yl)-2H-pyran-2-one (**320**), harziandione (**321**)	*V. parahaemolyticus*	MIC values ranging from 4 to 16 μg/mL.	
				3-Hydroxyharziandione (**317**)	*E. coli*	MIC value of 0.5 µg/mL	
				6-(3-Hydroxypent-1-en-1-yl)-2*H*-pyran-2-one (**320**)	*M. luteus*	MIC value of 1 µg/mL	
121	*Trichoderma koningiopsis* QA-3	*Artemisia argyi*	Qichun of the Hubei Province, China	15-Hydroxy-1,4,5,6-tetra-*epi-*koninginin G (**322**)	*Vibrio alginolyticus*	MIC, 1 μg/mL	[140]
				Koninginin U (**323**), 14-ketokoninginin B (**324**)	*Vibrio harveyi* and *Edwardsiella tarda*	MICs 4 and 2 µg/mL	
122	*Trichoderma atroviride* B7	*Colquhounia coccinea* var. *mollis*	Kunming Botanical Garden, Yunnan, China	Harzianol I (**325**)	*S. aureus*, *B. subtilis*, and *M. luteus*	EC_50_ 7.7, 7.7, and 9.9 μg/mL	[141]
123	*Trichoderma longibrachiatum* MD33	*Dendrobium nobile*	Jinshishi, Chishui, China	Dendrobine (**326**)	*Bacillus mycoides*, *B. subtilis*, and *Staphylococcus*	Zone of inhibition of 9, 12 and 8 mm	[142]
124	*Trichoderma virens* QA-8,	*Artemisia argyi*	Qichun of Hubei Province in central China	Trichocadinins B-D and G (**327**–**330**)	*E. coli* EMBLC-1, *Aeromonas hydrophilia* QDIO-1, *Edwardsiella tarda* QDIO-2, *E. ictarda* QDIO-10, *Micrococcus luteus* QDIO-3, *P. aeruginosa* QDIO-4, *Vibrio alginolyticus* QDIO-5, *V. anguillarum* QDIO-6, *V. harveyi* QDIO-7, *V. parahemolyticus* QDIO-8, and *V. vulnificus* QDIO-9	MIC in the range of 8–64 μg/mL	[143]
				Trichocadinin G (**330**)	*Ed. tarda* and *V. anguillarum*	MIC values of 1 and 2 μg/mL	
125	*Trichoderma koningiopsis* A729	*Morinda officinalis*		Koninginols A-B (**331**–**332**)	*B. subtilis*	MIC values of 10 and 2 μg/mL	[144]
126	*Trichoderma koningiopsis* QA-3	*Artemisia argyi*	Qichun	Ent-koninginin A (**333**)	*V. vulnificus*	MIC, 4 μg/mL	[145]
				Ent-koninginin A (**333**), trichoketide A (**339**)	*E. coli*, *E. tarda*, *V. anguillarum*, and *V. parahemolyticus*	MICs ranging from 8 to 64 μg/mL	
				Ent-koninginin A (**333**), 1,6-di-*epi-*koninginin A (**334**), 15-hydroxykoninginin A (**335**), 10-deacetylkoningiopisin D (**336**), koninginin T (**337**), koninginin L (**338**), trichoketide A (**339**)	*E. coli*	MIC, 64 μg/mL each	
					*E. tarda*, *V. alginolyticus*, and *V. anguillarum*	MIC values ranging from 4 to 64 μg/mL	
	***Alternaria***						
127	*Alternaria alternata* ZHJG5	*Cercis chinensis*		Isotalaroflavone (**340**), 4-hydroxyalternariol-9-methyl ether (**341**), verrulactone A (**342**)	*Xanthomonas oryzae* pv. *Oryzae*, *Xanthomonas oryzae* pv. *oryzicola* and *Ralstonia solanacearum* (Rs)	MIC ranging from 0.5 to 64 μg/mL.	[146]
128	*Alternaria* sp. PfuH1	*Pogostemon cablin* (Pacholi).		Alternariol (**44**), altertoxin VII (**343**), altenuisol (**344**)	*S. agalactiae*	MIC, 9.3, 17.3 and 85.3 μg/mL	[147]
				Altenuisol (**344**)	*E. coli*	MIC, 128 μg/mL	
129	*Alternaria alternata* ZHJG5	*Cercis chinensis*		Alternariol (**44**), altenuisol (**344**), alterlactone (**345**), Dehydroaltenusin (**346**)	FabH of *Xanthomonas oryzae* pv. *oryzae* (Xoo)	IC_50_ values from 29.5 to 74.1 μM	[148]
					*Xanthomonas oryzae* pv. Oryzae	MIC values from 4 to 64 μg/mL.	
				Alternariol (**44**), alterlactone (**345**)	Rice bacterial leaf blight	a protective efficiency of 66.2 and 82.5% at the concentration of 200 μg/mL	
130	*Alternaria alternata* MGTMMP031	*Vitex negundo*	Madurai, Tamil Nadu, India	Alternariol Me ether (**347**)	*B. cereus*, *Klebsiella pneumoniae*	MIC, 30 µM/L	[149]
					*E. coli*, *Salmonella typhi*, *Proteus mirabilis*, *S. aureus* and *S. epidermidis*	MIC, 35 µM/L	
131	*Alternaria alternata*	*Grewia asiatica*		3,7-Dihydroxy-9-methoxy-2-methyl-6*H*-benzo[c]chromen-6-one (**348**)	*S. aureus* (ATCC 29213), VRE, and MRSA	MIC, 32, 32 and 8 μg/mL	[150]
				Alternariol (**44**)	*S. aureus* (ATCC 29213), VRE, and MRSA	MIC, 128, 128, and 64 μg/mL	
132	*Alternaria* sp. Samif01	*Salvia miltiorrhiza*	Beijing Medicinal Plant Garden, Beijing, China	Altenuisol (**344**), 4-hydroxyalternariol-9-methyl ether (**349**) and alternariol (**44**)	*A. tumefaciens*, *B. subtilis*, *Pseudomonas lachrymans*, *Ralstonia solanacearum*, *Staphylococcus hemolyticus* and *Xanthomonas vesicatorya*	MIC values in the range of 86.7–364.7 μM	[151]
133	*Alternaria* sp. Samif01	*Salvia miltiorrhiza*	Beijing, China	Alternariol 9-Me ether (**347**)	*Bacillus subtilis* ATCC 11562 and *Staphylococcus haemolyticus* ATCC 29970, *A. tumefaciens* ATCC 11158, *Pseudomonas lachrymans* ATCC 11921, *Ralstonia solanacearum* ATCC 11696, and Xanthomonas vesicatoria ATCC 11633	IC_50_ values varying from 16.00 to 38.27 g/mL	[152]
134	*Alternaria* sp. and *Pyrenochaeta* sp.,	*Hydrastis canadensis*	William Burch in Hendersonville, North Carolina	Altersetin (**350**), macrosphelide A (**351**)	*S. aureus*	MIC, 0.23, and 75 μg/mL	[153]
135	*Simplicillium lanosoniveum*	*Hevea brasiliensis*	Songkhla Province, Thailand	Simplicildones K (**352**)	*S. aureus* ATCC25923, MRSA	MIC, 128μg/mL	[154]
				Botryorhodine C (**353**), simplicildones A (**354**)	*S. aureus* ATCC25923, MRSA	MIC, 32 μg/mL each	
136	*Simplicillium* sp. PSU-H41	*Hevea brasiliensis*	Songkhla Province, Thailand	Botryorhodine C (**353**), simplicildone A (**354**)	*S. aureus*	MIC, 32 μg/mL each	[155]
				Botryorhodine C (**353**)	MRSA	MIC, 32 μg/mL	
	***Cladosporium***						
137	*Cladosporium cladosporioides*	*Zygophyllum mandavillei*	Al-Ahsa, Saudi Arabia	Isocladosporin (**355**), 5′- hydroxyasperentin (**356**), 1-acetyl-17-methoxyaspidospermidin-20-ol (**357**), and 3-phenylpropionic acid (**358**)	*Xanthomonas oryzae* and *Pseudomonas syringae*	MIC values in the range of 7.81 to 125 µg/mL	[156]
138	*Cladosporium sphaerospermum* WBS017	*Fritillaria unibracteata* var. *wabuensis*	Western Sichuan Plateau of China	Cladosin L (**359**)	*S. aureus* ATCC 29213 and *S. aureus* ATCC 700699	MICs, 50 and 25 mM,	[157]
139	*Cladosporium* sp.	*Rauwolfia serpentina*		Me ether of fusarubin (**360**)	*S. aureus*, *E. coli*, *P. aeruginosa* and *B. megaterium*	Zone of inhibition of 27, 25, 24 and 22 mm (40μg/disk)	[158]
	***Pestalotiopsis***						
140	*Pestalotiopsis* sp. M-23	*Leucosceptrum canum*	Kunming Botanical Garden, China	11-Dehydro-3a-hydroxyisodrimeninol (**361**)	*B*. *subtilis*	IC_50_, 280.27 µM	[159]
141	*Pestalotiopsis* sp.	*Melaleuca quinquenervia*	Toohey Forest, Queensland, Australia	(1*S*,3*R*)-austrocortirubin (**362**), (1*S*,3*S*)-austrocortirubin (**363**), 1-deoxyaustrocortirubin (**364**)	Gram-pos.	100 μM	[160]
142	*Neopestalotiopsis* sp.			Neopestalotins B (**365**)	*B. subtilis*, *S. aureus*, *S. pneumoniae*	MIC, 10, 20, and 20 μg/mL	[161]
	***Phoma***						
143	*Phoma cucurbitacearum*	*Glycyrrhiza glabra*	Jammu (J&K).	Thiodiketopiperazine derivatives (**366**) and (**367**)	*S. aureus* and *Streptococcus pyogenes*	IC_50_, 10 μM	[162]
144	*Phoma* sp. JS752	*Phragmites communis*	Seochun, South Korea	Barceloneic acid C (**368**)	*Listeria monocytogenes* and *Staphylococcus pseudintermedius*	MIC, 1.02 μg/mL each	[163]
145	*Setophoma* sp.,	*Psidium guajava* fruits		Thielavins T (**369**), U (**370**) and V (**371**)	*S. aureus* ATCC 25923	MIC, 6.25, 50, and 25 μg/mL	[164]
	***Colletotrichum***						
146	*Colletotrichum gloeosporioides* B12	*Illigera rhodantha*	Qionghai City, Hainan Province, China	Colletolides A (**372**) and B (**373**), and 3-methyleneisoindolinon (**374**)	*Xanthomonas oryzae* pv. *oryzae*,	MIC, 128 μg/mL each	[165]
				Sclerone (**375**)	*X. oryzae* pv. *oryzae*	MIC, 64 μg/mL	
147	*Colletotrichum* sp. BS4	*Buxus sinica*	Guangzhou, Guangdong Province, China	Colletotrichones A (**376**)	*E. coli* and *B. subtilis*	MIC, 1.0 and 0.1 μg/mL	[166]
				Colletotrichone B (**377**)	*S. aureus* (DSM 799)	MIC, 5.0 μg/mL	
				Colletotrichone C (**378**)	*E. coli*	MIC, 5.0 μg/mL	
	**Minor Taxa of Anamorphic Ascomycetes**						
148	*Rhizopycnis vagum* Nitaf22 (synonym *Acrocalymma vagum)*	*Nicotiana tabacum*	Agricultural University Beijing China	Rhizopycnolide A (**379**)	*A. tumefaciens*, *B. subtilis*, and *P. lachrymans*	MICs 100, 75, and 100 μg/mL	[167]
				Rhizopycnin C (**380**), penicilliumolide D (**384**), alternariol (**44**)	*A. tumefaciens*, *B. subtilis*, *Pseudomonas lachrymans*, *Ralstonia solanacearum*, *Staphylococcus hemolyticus*, and *Xanthomonas vesicatoria*,	MICs in the range 25–100 μg/mL	
				Rhizopycnin D (**381**)	*A. tumefaciens*, *B. subtilis*, and *R. solanacearum*,	MIC 50 μg/mL each,	
					*X. vesicatoria*	MIC, 75 μg/mL.	
				Palmariol B (**383**), Alternariol 9-methyl ether (**347**)	*A. tumefaciens*, *B. subtilis*, *P. lachrymans*, *R. solanacearum*, and *X. vesicatoria*,	IC_50_ values in the range 16.7−34.3 μg/mL	
				TMC-264 (**382**)	*B. subtilis*	MIC 50 μg/mL	
149	*Rhizopycnis vagum* Nitaf22 (synonym *Acrocalymma vagum*)	*Nicotiana tabacum*	China Agricultural University, Beijing	Rhizoperemophilane K (**385**), 1α-hydroxyhydroisofukinon (**386**), 2-oxo-3-hydroxyeremophila-1(10),3,7(11), 8-tetraen-8,12-olide (**387**)	*A. tumefaciens*, *B. subtilis*, *P. lachrymans*, *R. solanacearum*, *S. haemolyticus*, and *X. vesicatoria*,	MIC, 32~128 μg/mL	[168]
150	*Rhizopycnis vagum* Nitaf22 (synonym *Acrocalymma vagum*)	*Nicotiana tabacum*	China Agricultural University (CAU), Beijing 100101, China	Rhizopycnis acid A (**388**)	*A. tumefaciens*, *B. subtilis*, *P. lachrymans*, *R. solanacearum*, *S. hemolyticus* and *X. vesicatoria*	MICs, 20.82, 16.11, 23.48, 29.46, 21.11, and 24.31 µg/mL	[169]
				Rhizopycnis acid B (**389**)		MICs, 70.89, 81.28, 21.23, 43.40, 67.61, and 34.86 µg/mL	
151	*Leptosphaeria* sp. XL026	*Panax notoginseng*	Shijiazhuang, Hebei province, China	Leptosphin B (**390**), conidiogenone C (**391**), conidiogenone D (**392**), conidiogenone G (**393**)	*B. cereus*	MICs 12.5–6.25 μg/mL	[170]
				Conidiogenone D (**392**)	*P. aeruginosa*	MIC, 12.5 μg/mL	
152	*Lophiostoma* sp. Eef-7	*Eucalyptus exserta.*		Scorpinone (**394**), 5-deoxybostrycoidin (**395**)	*Ralstonia solanacearum*	Zone of inhibition of 9.86 and 9.58 mm at 64 µg concentration	[171]
	*Lophiostoma* sp. Sigrf10	*Siraitia grosvenorii*	Guangxi Province of China	(8*R*,9*S*)-dihydroisoflavipucine (**396**), (8*S*,9*S*)-dihydroisoflavipucine (**397**)	*B. subtilis*, *A. tumefaciens*, *Ralstonia solanacearum*, and *Xanthomonas vesicatoria*	IC_50_ in the range of 35.68–44.85 µM	[172]
153	*Cytospora chrysosperma*	Hippophae rhamnoides		Cytochrysin A (**398**)	Enterococcus faecium	MIC, 25 μg/mL	[173]
				Cytochrysin C (**399**)	MRSA	MIC, 25 μg/mL	
154	*Microsphaeropsis* sp. *Seimatosporium* sp.	*Salsola oppositifolia*	Gomera, Spain	Microsphaerol (**400**)	*B. megaterium* and *E. coli*,	Zone of inhibition 8 and 9 mm at 0.05 mg concentration	[174]
				Seimatorone (**401**)	*B. megaterium* and *E. coli*,	Zone of inhibition 3 and 7 (partial) mm at a 0.05 mg concentration	
155	*Epicoccum nigrum* MK214079	*Salix* sp.	*Caucasus mountains* Lago-Naki, Russia	Epicocconigrone A (**402**), epipyrone A (**403**), and epicoccolide B (**404**)	*S. aureus* ATCC 29213	MIC values ranging from 25 to 50 μM	[175]
156	*Epicoccum nigrum*	*Entada abyssinica*	Balatchi (Mbouda), in the West region of Cameroon	p-Hydroxybenzaldehyde (**223**)	*S. aureus*, *B. cereus*, *P. aeruginosa*, and *E. coli*	MICs 50, 25, 50, and 25 µg/mL	[176]
				Beauvericin (**267**)	*S. aureus*, *B. cereus*, and *Salmonella typhimurium*	MICs 3.12, 12.5, and 12.5 µg/mL	
				Indole-3-carboxylic acid (**405**)	*S. aureus* and *E. faecalis*	MIC values of 6.25 and 50 µg/mL	
				Quinizarin (**406**)	*S. aureus*, *B. cereus* St	MIC values of 50 µg/mL each	
157	*Stemphylium lycopersici*	*S. tonkinensis*		Xylapeptide B (**407**)	*B. subtilis*, *S. aureus* and *E. coli*	MIC, 12.5, 25 and 25 μg/mL	[177]
				Cytochalasin E (**408**)	*B. subtilis*, *S. aureus*, *B. anthracis*, *S. dysenteriae*, and *E. coli*	MIC 12.5 to 25 μg/mL	
				6-Heptanoyl-4-methoxy-2H-pyran2-one (**409**)	*S. paratyphi* B	MIC, 12.5 μg/mL	
				(–)-5-Carboxymellein (**410**)	*B. subtilis*, *S. aureus*, *B. anthracis*, *S. dysenteriae*, *S. paratyphi*, *E. coli* and *S. paratyphi B*	MIC values from 12.5 to 25 μg/mL	
158	*Stemphylium globuliferum*,	*Juncus acutus*	Egypt	Dihydroaltersolanol C (**411**)	*S. aureus*	MICs of 49.7 μM	[178]
159	*Lecanicillium* sp. (BSNB-SG3.7 Strain)	*Sandwithia guyanensis*	St Elie, France.	Stephensiolides I (**412**), D (**413**), G (**414**), stephensiolide F (**415**)	MRSA	MICs 4, 32, 16 and 32 μg/mL	[179]
160	*Nigrospora sphaerica*	*Adiantum philippense*	Western Ghats region near Virajpete, India	Phomalactone (**416**)	*E. coli* and *X. campestris*	MIC 3.12 μg/mL	[180]
					*S. typhi*, *B. subtilis*, *B. cereus*, and *K. pneumonia*	MIC value of 6.25 μg/mL	
					*S. aureus*, *S. epidermidis*, and *C*. *albicans*	MIC of 12.5 μg/mL	
161	*Nigrospora* sp. BCC 47789	*Choerospondias axillaris*	Khao Yai National Park, Nakhon Ratchasima Province, Thailand	Nigrosporone B (**417**)	*M. tuberculosis*, *B. cereus* and *E. faecium*	MICs 172.25, 21.53 and 10.78 μM	[181]
162	*Curvularia sorghina* BRIP 15900)	*Rauwolfia macrophylla*	Mount Kalla in Cameroon	2′-Deoxyribolactone (**419**), hexylitaconic acid (**419**)	*E. coli*, *Micrococcus luteus*, *Pseudomonas agarici* and *Staphylococcus warneri*	MIC ranging between 0.17 μg/mL and 0.58 μg/mL	[182]
163	*Curvularia lunata*	*Paepalanthus chiquitensis*	Serra do Cipó, in Minas Gerais State, Brazil	Triticones E (**420**), F (**421**)	*E. coli*,	MIC 62.5 μg/mL	[183]
164	*Bipolaris* sp. L1-2	*Lycium barbarum*	Ningxia Province, China	Cochlioquinones B (**422**), C (**423**), isocochlioquinones (**424**)	*B. subtilis*, *C. perfringens*, and *P. viridiflava*	MICs 26 μM	[184]
165	*Bipolaris eleusines*	Potatoes	nursery of Yunnan Agricultural University, Kunming, Yunnan China	(S)-5-Hydroxy-2-(1-hydroxyethyl)-7-methylchromone (**425**), 5,7-dihydroxyl-2,6,8-trimethylchromone (**426**)	*Staphylococcus aureus* subsp. *Aureus*	inhibition rates of 56.3 and 32 %, at the concentration of 128 μg/mL	[185]
166	*Bionectria* sp. Y1085,	*Huperzia serrata*	Xichou County, Yunnan Province, China	Bionectin D (**427**), bionectin E (**428**), verticillin A (**430**), sch 52901 (**429**), gliocladicillin C (**431**)	*E. coli*, *S. aureus*, and *S. typhimurium* ATCC 6539,	MIC values ranging from 6.25–25 µg/mL	[186]
167	*Cylindrocarpon* sp.,	*Sapium ellipticum*	Haut Plateaux region, Cameroon	Pyrrocidine A (**432**)	*S. aureus*, ATCC 25923, *S. aueus* ATCC 700699, *S. aueus* ATCC 700699, *E. faecalis* ATCC 29212, *E. faecalis* ATCC 51299, *E. faecium* ATCC 35667, *E. faecium* ATCC 700221	MIC values ranging from 0.78 to 25 μM	[187]
				19-O-Methylpyrrocidine B (**433**)	*S. aureus* ATCC25923 and ATCC700699	MIC, 50 and 25 μM,	
168	*Eupenicillium* sp. LG41.9 treated with HDAC inhibitor, nicotinamide (15 mg/100 mL)	*Xanthium sibiricum*	Taian, Shandong Province, China	Eupenicinicol C (**434**)			[188]
				Eupenicinicol D (**435**),	*S. aureus*	MIC 0.1 μg/mL,	
				Eujavanicol A (**436**)	*E. coli*	MIC 5.0 μg/mL	
				Eupenicinicol A (**437**)			
169	*Dendrothyrium variisporum*	*Globularia alypum*	Ain Touta, Batna 05000, Algeria	2-Phenylethyl 3-hydroxyanthranilate (**438**)	*B. subtilis* and *M. luteus*	MICs 8.33 and 16.66 μg/mL	[189]
				2-Phenylethyl anthranilate (**439**)	*B. subtilis* and *M. luteus*	66.67 μg/mL each	
170	*Exserohilum rostratum*	*Phanera splendens* (Kunth) Vaz		Ravenelin (**440**)	*Bacillus subtilis* and *Staphylococcus aureus*	MICs, 7.5 and 484 μM	[190]
171	*Exserohilum rostratum*	*Bauhinia guianensis*		Monocerin (**441**)	*P. aeruginosa*	MIC, 62.5 µg/mL	[191]
				Annularin I (**442**)	*E. coli* and *B. subtilis*	MIC, 62.50 and 31.25 µg/mL	
				Annularin J (**443**)	*E. coli* and *B. subtilis*	MIC, 62.50 µg/mL each	
	**Basidiomycete**						
172	*Psathyrella candolleana*	*Ginkgo biloba*		Quercetin (**444**), carboxybenzene (**445**), and nicotinamide (**446**)	*S. aureus*	MIC 0.3906, 0.7812 and 6.25 μg/mL	[192]
173	*Irpex lacteus* DR10-1	*Distylium chinense*	Banan district of Chongqing in the TGR area, China	Irpexlacte A (**447**), irpexlacte B-D (**448**–**450**)	*P. aeruginosa*	MIC values ranging from 23.8 to 35.4 μM	[193]
	**Zygomycetes**						
174	*Mucor irregularis*			Chlorflavonin (**451**)			[194]

## 4. Methods Used for Activation of Silent Biosynthetic Genes

It has been reported that fungi have various unexpressed gene clusters related to bioactive secondary metabolites, which do not express in mass multiplications of the axenic form [213,214]. The expression of such gene clusters directly or indirectly depends on the surrounding environment of the microorganism. In axenic form, various induction or activation signals are or may be absent for some bioactive molecule production in the culture, which are usually present in natural habitats [215]. Such biosynthetic gene clusters (BGC) are part of the heterochromatin of fungal chromosomes, which do not express at laboratory conditions [216].

To induce such silent biosynthetic gene clusters two major approaches have been reported, including pleiotropic- and pathway-specific approaches, which include various techniques like knocking down, mutation induction [217], co-culture methods [218], heterologous expression [219,220], interspecies crosstalk [221], one strain many compounds (OSMAC) [222] and epigenetic manipulation [223]. Changes in media composition and physical factors like pH, temperature, light, salt concentration, metal and elicitor also support the induction of silent BGC and improve production of secondary metabolites in microbes. The generation of various types of stresses significantly affects the metabolic activities of growing culture and microbes to release compounds for their survival under stress conditions. Changes in physical conditions or stresses impacted gene regulation by upregulating or downregulating the gene expression [126,224]. Nowadays, high throughput elicitor screening technique (HiTES) is also employed to save time in exposing culture against various types of elicitors. In this technique selected culture is grown in 96 well plates with various elicitors in each well and after the incubation period metabolites are identified by mass spectrometry or assay system.

The mutation is one of the other approaches to induce silent biosynthetic gene clusters (BGC). Mutation in RNA polymerase genes and ribosomal proteins changes the transcription and translational process and upregulates the expression of biosynthetic gene clusters. Some of the genes related to biosynthetic gene clusters are silent from decades and overexpression of *adpA*, a global regulatory gene, induced the expression of silent lucensomycin in *Streptomyces cyanogenus* S136 [225]. Cloning is another type of molecular technique used to express the silent BGC incompatible strains. In the cloning method, isolation of high-quality DNA, fragmentation, library construction and development of suitable expression vectors for large sequences of BGC is a challenging task and many groups are working on this aspect [226]. In addition to this, use of bioinformatics also helps in direct cloning of silent BGCs and their expression for secondary metabolites production. Development of various bioinformatics tools such as PRISM3, BiG-SCAPE and anti-SMASH etc facilitated the scientist to identify bioactive gene clusters in unknown strains without time consumption used in identification of active BGC sites [227]. The CRISPR-Cas system is also a excellent tool for cloning system or genome editing that provides better expression of silent BGC in comparison to conventional molecular techniques [228]. Similarly, promoter engineering, transcriptional regulation engineering and ribosome engineering also support the activation of silent BGC through molecular approaches [229]. Recent use of Cpf1 nuclease in genome editing was also found to be a suitable tool for induction of silent BGC [230].

### 4.1. Epigenetic Modification

On the other hand, epigenetic modification played a great role to induce the silent genes related to bioactive molecules, which are actively produced under symbiotic interactions. Epigenetics refers to the study of DNA sequences that do not changes in mutation but change in gene function [231]. The epigenetic regulations such as methylation, demethylation, acetylation, deacetylation and phosphorylation of histones also regulate the transcription of biosynthetic genes of fungi and are helpful in silencing or expression of such genes related to the production of secondary metabolites [232]. The importance of epigenetic regulation in secondary metabolite production by fungi has been shown in a few reports published [231,233,234,235,236]. Modification or alteration in DNA or chromatin changes the expression level of the selected genes, which directly impacted the biosynthesis of the metabolites in the strain.

### 4.2. The Co-Culture Strategy

The co-culture is another method to induce the silent biosynthetic gene clusters by interspecies cross-talking of microorganisms. In this method, various combinations of inducers with producer microbial strains are screened for the production of novel molecules. In co-culture technique real-time bioactivity screening can also be measured by the growth of pathogen as co-culture [218]. Recently, Kim et al. [237] reviewed the co-culture interactions of fungi with various actinomycetes for induction of silent biosynthetic gene clusters and reported upregulation and production of novel antibiotics and bioactive compounds. Co-culturing of microbes provides the habitat type environment to producers and helps to promote silent BGCs by producing signal molecules. Exchange of chemical signals of growing organisms is helpful in the induction of defense molecules and other silent BGC, and usually results in the production of new natural products or secondary metabolites in the culture [238].

Another concept has also been introduced to elicit the production of silent secondary metabolites by scaffold technique. In this technique, two types of scaffold named cotton and talc powder are introduced in the medium which physically interacts with the grown culture and elicit chemical signaling of the culture and activate the production of silent BGC. The addition of scaffold in the medium supports the grown culture in formation of biofilm and provides a mimic architecture of natural habitat [239,240]. The addition of scaffold in medium affects the morphology of growing culture and sporulation pattern like an agglomeration of spores, oxygen diffusion in comparison to non-scaffold containing medium and then facilitates more metabolites production [241].

### 4.3. OSMAC

In the OSMAC technique different cultivation approaches are applied to induce silent bioactive gene clusters to promote more production of secondary metabolites including media variations, variation in media composition, co-cultivation with other strains and variations in cultivations strategy [222,242]. Variation in growth conditions also supports the induction of silent biosynthetic gene clusters and the production of novel compounds. Scherlach and Hertweck [243] and Scherlach et al. [244] reported the production of novel aspoquinolone and aspernidine alkaloid compounds from *Aspergillus nidulans* by variation in growth conditions.

## 5. Conclusions

Increasing resistance among microbial pathogens against existing antibiotics has been a major concern during the past several decades. Scientists are exploring new sources of novel antibiotics and other bioactive compounds that can curb pathogenic infections and overcome antimicrobial resistance. Endophytic fungi have been reported to secrete a wide spectrum of bioactive compounds to counter pathogens. In the current review, we have reported 453 new bioactive compounds, including volatile compounds, isolated during the period of 2015-21 from various endophytic fungi belonging to the Ascomycetes, Basidiomycetes, and Zygomycetes classes. Newly reported bioactive compounds have shown activity against various pathogenic bacteria and shown scaffold similarity with alkaloids, benzopyranones, chinones, cytochalasins, mullein, peptides, phenols, quinones, flavonoids, steroids, terpenoids, sesquiterpene, tetralones, xanthones, and others. The lowest in vitro activity in terms of minimum inhibitory concentrations (MICs) in the 0.1–1 µg/mL range against various pathogens was reported for the compounds vochysiamides A (**23**) and B (**24**), colletotrichone A (**376**), 15-hydroxy-1,4,5,6-tetra-*epi*-koninginin G (**322**), trichocadinin G (**330**) and eupenicinicol D (**435**). Compounds like fusarubin (**287**), chetomin (**62**), chaetocochin C (**63**), and dethiotetra(methylthio)chetomin (**64**), pretrichodermamide A (**296**), terpestacin (**105**), fusaproliferin (**106**), mutolide (**108**), isoeugenitol (**120**) and nigrosporone B (**417**) were reported to have significant in vitro anti-mycobacterial activity and could be developed as potential drugs against resistant mycobacterial infections. The production of such bioactive compounds and their activity is also affected by the surrounding environment and conditions. Various techniques related to induction of silent gene clusters such as epigenetic modifications, co-culture, OSMAC and mutation have been reported

In most of cases only in vitro data against a limited number of bacteria is reported and there is a great need for extensive in vitro studies including their mode of action, kill curve studies, mutation induction frequency, resistance occurrence frequency studies, in vitro cytotoxicity and initial in vivo evaluation followed by formulation studies. Moreover, there is also a need to perform extensive in vitro efficacy testing studies using panels of references strains and clinical strains to establish MIC_90_ and MIC_50_ values. Generation of comparative efficacy data with benchmark clinical compounds is very important from a further development perspective. These extensive studies also help to generate data for understanding the scope of work when we consider such potent molecules for semisynthetic work. The exact studies to be performed during screening and further shortlisting of semi-synthetic molecules can be extracted from this initial extensive work.

Still, more research is required to investigate a new generation of antibiotics which can control the increasing resistance of infectious microorganisms in a sustainable manner. The success of this exploration depends upon screening more and more endophytic fungi and ways of their isolation, fermentation and scale-up.

## Data Availability

Not Applicable.
